# The International Headache Congress – IHS and EHF joint congress 2021: late breaking abstracts: Virtual. 8-12 September 2021

**DOI:** 10.1186/s10194-021-01319-2

**Published:** 2021-11-18

## AL061 Trigeminal nerve: functional brainstem somatotopy during nociception

### L. M. Sturm, H. Basedau, J. Mehnert, A. May

#### Institute of Systems Neurosciences, Hamburg, Hamburg, Germany

##### **Correspondence:** L. M. Sturm

Objective

The trigeminal nerve plays a crucial role in the pathogenesis of primary headache disorders. Its three branches (V1, V2, V3) distribute peripherally in U-shaped dermatomes and enter the brainstem in somatotopic order from ventral (V1) to dorsal (V3). Besides the peripheral dermatomes (V1-V3), animal studies suggest an alternative dermatome in the central presentation within the spinal trigeminal nucleus (STN). Fibers of perioral regions are represented more rostrally and those of the periauricular regions more caudally in the STN ("onion-shaped pattern"). In our current study we investigated the somatotopic arrangement of the trigeminal nerve within the brainstem in human beings.

Methods

A 3-Tesla functional magnetic resonance imaging (fMRI) study was conducted in 26 healthy volunteers. Four areas on the left hemiface were stimulated with nociceptive electrical stimulation. Stimulated areas were chosen based on their representation within in the brainstem to ensure adequate spatial resolution between each other within the STN.

Results

We found distinct blood oxygen level-dependent (BOLD)-signal activation for each stimulus site within the spinal trigeminal nucleus.

Conclusion

Our results provide non-invasive, functional evidence in humans to support the long hypothesized somatotopic arrangement along the rostro-caudal axis within the brainstem with an onion -shaped pattern in the face.

## AL062 Galcanezumab changes somatosensory perception exclusively in the face, whereas heat pain threshold before treatment predicts efficacy

### K. P. Peng, H. Basedau, T. Oppermann, A. May

#### University Medical Center Hamburg-Eppendorf, Department of Systems Neuroscience, Hamburg, Germany

##### **Correspondence:** K. P. Peng

**Background:** Antibodies to the calcitonin gene-related peptide and its receptor (CGRP-mAb), are emerging migraine treatments. We hypothesized that the CGRP-mAb, galcanezumab, modulates peripheral pain processing and that possible differences of sensory thresholds discriminate clinical responses.

**Methods:** Twenty-six migraine patients were recruited. Quantitative sensory tests over the right V1 dermatome and forearm were tested before and 2-3 weeks after galcanezumab administration. A clinical responder was defined as having at least a 30% reduction in headache frequency after 3 months of treatment. Predictors for clinical response were calculated using binary logistical regression models.

**Results:** Heat pain threshold (HPT) and mechanical pain threshold (MPT) increased significantly (p<0.05) after galcanezumab exclusively in the V1 dermatome, but not on the forearm. These changes over time (i.e., between 1st and 2nd measurement) did not differ regarding clinical response. Baseline HPT (before galcanezumab) predicted a clinical response and inversely correlated with the baseline headache frequency.

**Conclusion:** Galcanezumab modulates pain thresholds specifically in the V1 dermatome, but this modulation is independent of clinical response. Instead, baseline (heat) pain threshold predicts clinical response, suggesting that clinical response may be associated with individual susceptibility and disease activity.

## AL063 The PACAP pathway is independent of CGRP in mouse models of migraine: A possible new drug target

### C. Ernstsen^1^, S. Christensen^1^, R. Rasmussen^1^, B. Nielsen^1^, I. Jansen-Olesen^1^, J. Olesen^1^, D. M. Kristensen^1,2^

#### ^1^Danish Headache Center, Department of Neurology, Glostrup, Denmark; ^2^University of Rennes, Inserm, EHESP, Irset (Institut de Recherche en Santé, Environnement et Travail), Rennes, France

##### **Correspondence:** C. Ernstsen

**Objective:** Calcitonin gene-related peptide (CGRP) antagonizing drugs signify a major advance in migraine treatment. Yet, ≥50% of patients do not benefit from monoclonal antibodies against CGRP or its receptor. We test the hypothesis that a closely related peptide, pituitary adenylate cyclase-activating peptide (PACAP-38), works independently of CGRP and thus might represent a new alternative drug target. **Methods:** We used mouse models of provoked migraine-like pain based upon multiple stimulations and subsequent measurement of tactile sensitivity responses. Genetically modified mice lacking either functional CGRP receptors (*Ramp1* knockout) or TRPA1 channels (*Trpa1* kockout) were used together with CGRP-targeting antibodies and chemical inhibitors. *Ex vivo* myograph studies were used to measure dilatory responses to CGRP and PACAP-38 in mouse carotid arteries. **Results:** PACAP-38 provoked significant hypersensitivity and dilated the carotid arteries independently of CGRP. In contrast, glyceryl trinitrate (GTN)-induced hypersensitivity is CGRP dependent. Contrary to other migraine-inducing substances like GTN, cilostazol, and levcromakalim, PACAP-38-induced hypersensitivity worked only partially through KATP channel inhibition. **Conclusion:** These findings establish the PACAP-38 pathway as distinct from the migraine provoking agents CGRP and GTN. PACAP antagonism may therefore be a novel therapeutic target, of particular interest in patients unresponsive to CGRP antagonists.

## AL064 Impact on the pain syndrome of the degree of invasion of pituitary adenoma into the cavernous sinus according to the knosp scale (before and after transphenoidal endoscopic resection)

### M. Kurnukhina^1^, E. Semina^2^, V. Cherebillo^1^

#### ^1^First Pavlov State Medical University of St. Petersburg, Neurosurgery, St. Petersburg, Russian Federation; ^2^First Pavlov State Medical University of St. Petersburg, Medical Faculty, St. Petersburg, Russian Federation

##### **Correspondence:** M. Kurnukhina

**Summary.**Pain syndrome is one of the most frequent complaints of patients with pituitary adenoma before and after surgery. Correlation between the severity of pain syndrome and the degree of invasion of pituitary adenoma into the cavernous sinus has not been studied in the literature before.

**Purpose**.Assessment of the impact the degree of invasion into the cavernous sinus according to the Knosp Scale(KS) on the pain syndrome.

**Methods**.A clinical study of 200 patients was conducted. All the studied patients underwent transsphenoidal endoscopic resection of pituitary adenoma.The analysis of changes in pain syndrome was carried out before and after surgery. The subjects were aged 18-64 years (median 42,4 years). We used scale VAS, EORTC QLQ-C 30 questionnaire(pain scale), KS.

**Results**.We found a significant decrease in headache complaints after surgical treatment (diffuse headaches-74,8 % and 8,6%,headache of a certain localization-9.8% and 1,4% - before and after surgery, accordingly)(p<0,05).Patients Grade III-IV KS noted a decrease in the severity of pain syndrome from 7±2,2 to 1,2±0,8;9,2±3,4 to 2±1,4 points on the VAS scale (p<0,05) according, after surgery.According to the EORTC QLQ-C 30 in the late postoperative period, patients Grade IV KS had a more pronounced pain syndrome (r=0.38;p<0,05)

**Conclusion**.Patients Grade III-IV KS are more likely to report pain syndrome. Transsphenoidal resection leads to a significant decrease in the severity of the pain syndrome

## AL065 Nummular headache endophenotyping

### O. I. Ogunlaja^1^, P. J. Goadsby^1,2^

#### ^1^NIHR-Wellcome Trust King’s Clinical Research Facility, London, United Kingdom; ^2^University of California, Department of Neurology, Los Angeles, CA, United States

##### **Correspondence:** O. I. Ogunlaja

**Objectives**: Nummular headache (NH) is an uncommon primary headache disorder characterised by a small, round or elliptical, well circumscribed area of intermittent or continuous cranial pain. We set out to determine in how many cases the presence of associated features questioned the diagnosis.

**Methods**: We audited cases of patients seen at King"s College Hospital headache clinic who met ICHD 3 criteria for the diagnosis of NH, as well as those with headache in a nummular distribution. Three met ICHD 3 criteria for NH and two had a diagnosis of migraine.

**Results:** The mean age of onset in patients with NH was 35 (range, 12-54), while the two patients with a nummular type distribution of pain were age 34 and 59 at headache onset. Location varied and multifocal areas of nummular pain were present in three of the patients: 2 with NH, 1 with migraine. Notably, in all three patients with NH, the pain was episodic while in those with migraine it was chronic and persistent. Of the three patients who met diagnostic criteria for NH, one had onset in pregnancy, and this resolved following delivery. The second, had onset 6 months after puberty and the condition markedly improved at age 18 while they were on testosterone for gender transition.

**Conclusion:** The pathophysiology of NH is uncertain. Some cases overlap phenotypically with other primary headache disorders and may represent an endophenotype other disorders.

## AL066 Interictal pontine metabolism in migraine without aura patients: a 3 tesla proton magnetic resonance spectroscopy study

### S. Younis^1^, A. Hougaard^1^, C. E. Christensen^1^, M. B. Vestergaard^2^, O. B. Paulson^3^, H. B. W. Larsson^2^, M. Ashina^1^

#### ^1^Danish Headache Center, Neurology, Glostrup, Denmark; ^2^Functional Imaging Unit, Clinical Physiology, Glostrup, Denmark; ^3^Neurobiology Research Unit, Neurology, Copenhagen, Denmark

##### **Correspondence:** S. Younis

Objective: In the pons, glutamatergic mechanisms are involved in regulating inhibitory descending pain modulation, serotoninergic neurotransmission as well as modulating the sensory transmission of the trigeminovascular system. Migraine involves altered pontine activation and structural changes, and biochemical, genetic and clinical evidence suggests that altered interictal pontine glutamate levels may be an important pathophysiological feature of migraine abetting to attack initiation.

Methods: Thirty-four migraine without aura patients were scanned outside attacks using a proton magnetic resonance spectroscopy protocol optimized for the pons at 3T. The measurements were performed on two separate days to increase accuracy and compared to similar repeated measurements in 16 healthy controls.

Results: We found that interictal glutamate levels in the pons of migraine patients were not different from healthy controls (p=0.098), while total creatine levels were markedly increased in patients (9%, p=0.009). There was no correlation of glutamate or total creatine levels to migraine frequency, days since the last attack, or usual pain intensity of attacks.

Conclusion: Migraine is not associated with altered interictal pontine glutamate levels. However, the novel finding of increased total creatine levels suggests that disequilibrium in the pontine energy metabolism could be an important feature of migraine pathophysiology.

## AL067 Predictors of health-related quality of life in patients with cluster headache during the active periods using the time trade-off method

### S. K. Kim^1^, S. Kim^1^, M. K. Chu^2^, B. K. Kim^3^, P. W. Chung^4^, M. J. Lee^5^, Y. Choi^6^, J. W. Park^7^, B. S. Kim^8^, K. Oh^9^, H. S. Moon^4^, T. J. Song^10^, J. Y. Ahn^11^, J. H. Sohn^12^, K. S. Lee^13^, K. Y. Park^14^, J. M. Chung^15^, C. S. Chung^5^, S. J. Cho^16^

#### ^1^Gyeongsang National University College of Medicine, Department of Neurology, Jinju, South Korea; ^2^Severance Hospital, Seoul, South Korea; ^3^Eulji University, Seoul, South Korea; ^4^Kangbuk Samsung Hospital, Seoul, South Korea; ^5^Neuroscience Center, Samsung Medical Center, Seoul, South Korea; ^6^Presbyterian Medical Center, Jeonju, South Korea; ^7^Uijeongbu St. Mary’s Hospital, Uijeongbu, South Korea; ^8^Bundang Jesaeng General Hospital, Seoul, South Korea; ^9^Korea University College of Medicine, Seoul, South Korea; ^10^Ewha Womans University School of Medicine, Seoul, South Korea; ^11^Seoul Medical Center, Seoul, South Korea; ^12^Chuncheon Sacred Heart Hospital, Chuncheon, South Korea; ^13^Seoul St. Mary’s Hospital, Seoul, South Korea; ^14^Chung-Ang University Hospital, Seoul, South Korea; ^15^Inje University College of Medicine, Seoul, South Korea; ^16^Dongtan Sacred Heart Hospital, Hwaseong, South Korea

##### **Correspondence:** S. K. Kim

*Background and Objective:* The objective of this study was to identify the factors affecting QoL in Cluster headache (CH) patients during the active periods. *Methods:* The CH patients were enrolled from September 2016 to February 2021 in 16 headache clinics in Korea. The questionnaire conducted questions about their QoL using EQ-5D-3L with the time trade-off (TTO) method. The age-sex matched control groups consisted of patients with migraine were recruited. *Results:* A total of 425 CH patients during an active period were included. The CH patients had lower median scores of EQ-5D than healthy control and migraine patients (0.88 ± 0.43 vs. 0.99 ± 0.33 and 0.99 ± 0.43, *p < 0.000*). Fifty-eight (13.6%) CH patients scored moderate to severe QoL impairment by the TTO method. The QoL states in CH patients were associated with current smoking, the severity, frequency, and duration of pain, Generalized Anxiety Disorder-7 (GAD-7), Patient Health Questionnaire-9 (PHQ-9), Headache impact test-6, and Allodynia Symptom Checklist-12 scores. Multivariate logistic regression analyses revealed that the QoL states in CH patients were negatively correlated with the daily frequency of headache, duration of active periods, GAD-7, and PHQ-9 scores. *Conclusions:* Our results show that CH patients have a poorer QoL during active periods than the healthy control and migraine patients. Their QoL might be is associated with daily headache frequency, duration of active periods, anxiety, and depression.

## AL068 HMGB1, NLRP3, IL-6 and ACE2 Levels Are Elevated in COVID-19 with Headache: A Window to the Infection-Related Headache Mechanism

### H. Bolay^1^, O. Karadaş^2^, B. Öztürk^2^, R. Sonkaya^2^, B. Taşdelen^3^, T. Bulut^1^, Ö. Gülbahar^1^, A. Özge^3^, B. Baykan^4^

#### ^1^Gazi University, Medical Biochemistry, Ankara, Turkey; ^2^University of Health Sciences, Neurology, Ankara, Turkey; ^3^Mersin University, Neurology, Mersin, Turkey; ^4^Istanbul University, Neurology, Istanbul, Turkey

##### **Correspondence:** H. Bolay

AIM: Pathogenesis of COVID-19 -related headache is unknown, we investigated key systemic circulating inflammatory molecules and their clinical relations with headache. METHODS: This cross-sectional study enrolled 88 COVID-19 patients, hospitalized on a regular ward during the second wave of the pandemic. Clinical characteristics of COVID-19 patients were recorded, and laboratory tests were studied. RESULTS: The mean ages of 48 COVID-19 patients with headache and 40 COVID-19 patients without headache were comparable. COVID-19 patients with headache had significantly higher serum levels of HMGB1, NLRP3, ACE2, and IL-6 than COVID-19 patients without headache, whereas CGRP and IL-10 levels were similar. Ang II level was significantly decreased in the headache group. COVID-19 patients with headache showed an increased frequency of pulmonary involvement and COVID-19 was more frequently associated with weight loss, nausea, and diarrhea in patients with headache. Serum NLRP3 levels were correlated with headache duration and hospital stay, while headache response to paracetamol was negatively correlated with HMGB1 and positively associated with IL-10 levels. CONCLUSION: Stronger inflammatory response is associated with COVID-19 headache. Increased levels of the circulating inflammatory/nociceptive molecules like HMGB1, NLRP3, and IL-6 may play a role in the potential induction of the trigeminal system and headache secondary to SARS-CoV-2 infection.

## AL069 Galcanezumab has central effects in the migraine brain

### H. Basedau, L. M. Sturm, J. Mehnert, K. P. Peng, M. Schellong, A. May

#### University Medical Center Eppendorf, Hamburg, Department of Systems Neuroscience, Hamburg, Germany

##### **Correspondence:** H. Basedau

Monoclonal antibodies (mAb) targeting calcitonin gene-related peptides (CGRP) are a novel treatment for migraine prevention. Based on a previous functional magnetic resonance imaging (fMRI) study with the CGRP receptor mAb (erenumab), we hypothesised that galcanezumab, CGRP ligand mAb, would also alter trigeminal central pain processing and that responders to galcanezumab treatment would show specific (hypothalamic) modulation in contrast to non-responders. This study was pre-registered in the Open Science Framework.

We conducted an fMRI study in 26 migraine patients with an established trigeminal nociceptive paradigm with gaseous ammonia, in the same way as the previous erenumab study, and studied the patients before and 2-3 weeks after the administration of galcanezumab. We have found that galcanezumab reduces hypothalamic activation, this was also prominent in responders against non-responders. Erenumab and galcanezumab show different changes to trigemino-nociceptive central responses. The activity of the spinal trigeminal nucleus (STN) followed by trigemino-nociceptive stimulation before treatment covaries with the response to galcanezumab. Furthermore the connectivity between the STN and the hypothalamus is altered after galcanezumab administration.

Our results suggest that despite the impermeability of the blood-brain barrier to CGRP-mAb, treatment with mAb induces specific effects in the brain that may be part of its mechanism of efficacy in migraine treatment.

## AL070 International Consortium for Cluster Headache Genetics: two initiatives report first genome-wide association hits

### B. S. Winsvold^1,2,3^, A. V. E. Harder^4,5^, C. Fourier^6^, R. Noordam^7^, E. O'Connor^8^, C. Ran^6^, J. Vandrovcova^8^, J. A. Zwart^1,2,9^, H. Houlden^8^, G. M. Terwindt^4^, M. Matharu^10^, A. van den Maagdenberg^4,5^, A. C. Belin^6^, C. Int. Consortium for Cluster Headache Genetics^1^

#### ^1^Oslo University Hospital, Department of Research and Innovation, Division of Clinical Neuroscience, Oslo, Norway; ^2^Norwegian University of Science and Technology (NTNU), K. G. Jebsen Center for Genetic Epidemiology, Department of Public Health and Nursing, Faculty of Medicine and Health Sciences, Trondheim, Norway; ^3^Oslo University Hospital, Department of Neurology, Oslo, Norway; ^4^Leiden University Medical Center, Department of Neurology, Leiden, Netherlands; ^5^Leiden University Medical Center, Department of Human Genetics, Leiden, Netherlands; ^6^Karolinska Institutet, Department of Neuroscience, Stockholm, Sweden; ^7^Leiden University Medical Center, Department of Internal Medicine, Section of Gerontology and Geriatrics, Leiden, Netherlands; ^8^University College London, Department of Neuromuscular Diseases, Institute of Neurology, London, United Kingdom; ^9^University of Oslo, Institute of Clinical Medicine, Faculty of Medicine, Oslo, Norway; ^10^UCL Queen Square Institute of Neurology, Headache and Facial Pain Group, London, United Kingdom

##### **Correspondence:** B. S. Winsvold

**Objective:** To identify genetic risk variants for cluster headache (CH).

**Methods:** Two parallel genome-wide association studies (GWAS) of CH were conducted based on 1443 CH cases and 6748 controls from Sweden and UK, and 984 CH cases and 3257 controls from the Netherlands and Norway, respectively. Subsequently, the studies were combined in a first attempt to meta-analyse the data of the two initiatives.

**Results:** The two studies independently identified four genetic risk loci (p < 5 × 10-8) on chromosome (chr) 1 near the gene *DUSP10*, chr 2 near *MERTK*, chr 2 near *SATB2* and chr 6 near *FHL5*, with odds ratios around 1.5 (range 1.30 - 1.61). A first meta-analysis of the two studies suggested three additional loci on chr 7 near *ASZ1*, chr 10 near *PLC1* and on chr 19 near *KIR3DX1*.

**Conclusion:** The discovery of four risk loci for CH provides robust evidence that the disease has a genetic basis. Effect sizes are larger than those typically seen in GWAS of complex traits. The identification of the *FHL5*-locus, an established risk locus for migraine, besides loci specific to CH, may suggest a partly overlapping genetic basis for both disorders. Downstream analyses to obtain insight into disease mechanisms will require larger sample sizes. To this end, we have established the International Consortium for Cluster Headache Genetics, aiming to gather interested researchers and available samples for a large-scale GWAS meta-analysis of CH.

## AL071 Neurovascular contact plays no role in trigeminal neuralgia secondary to multiple sclerosis

### N. Noory^1^, E. A. Smilkov^2^, J. L. Frederiksen^3^, T. B. Heinskou^1^, A. S. S. Andersen^1^, L. Bendtsen^1^, S. Maarbjerg^1^

#### ^1^Danish Headache Center, Department of Neurology, Glostrup, Denmark; ^2^Rigshospitalet-Glostrup, Department of Radiology, Glostrup, Denmark; ^3^Rigshospitalet-Glostrup, Department of Neurology, Glostrup, Denmark

##### **Correspondence:** S. Maarbjerg

**Objective and Background**: A demyelinating plaque and neurovascular contact with morphological changes have both been suggested to contribute to the etiology of trigeminal neuralgia secondary to multiple sclerosis (TN-MS). The aim of this study was to confirm or refute whether neurovascular contact with morphological changes is involved in the etiology of TN-MS.

**Methods**: We prospectively enrolled consecutive TN-MS patients from the Danish Headache Center. Clinical characteristics were collected systematically. MRI scans were done using a 3.0 Tesla imager and were evaluated by the same experienced blinded neuroradiologist.

**Results:** Sixty-three patients were included. There was a low prevalence of neurovascular contact with morphological changes on both the symptomatic side (6 (14%)) and the asymptomatic side (4 (9%)), p = 0.157. Demyelinating brainstem plaques were more prevalent on the symptomatic side compared to the asymptomatic side (31 (58%) vs. 12 (22%), p < 0.001). A demyelinating plaque was highly associated with the symptomatic side (odds ratio =10.6, p = 0.002).

**Conclusions:** The primary cause of TN-MS is demyelination along the intrapontine trigeminal afferents. As opposed to classical trigeminal neuralgia, neurovascular contact does not play a role in the etiology of TN-MS. Microvascular decompression should generally not be offered to patients with TN-MS.

## AL072 Voice change and/or throat swelling as cranial autonomic symptoms in the primary headache disorders

### N. Karsan^1^, P. J. Goadsby^1,2^

#### ^1^King’s College, Headache Group, London, United Kingdom; ^2^University of California, Los Angeles, CA, United States

##### **Correspondence:** N. Karsan

**Objective**: We aimed to audit the reporting of voice change and throat swelling as potential cranial autonomic symptoms (CAS) associated with headache. **Methods:** Clinic letters of patients seen between 2016-May 2021 containing "voice' (*n*=65) or "throat" (*n*=151) were selected; those mentioning voice change and/or throat swelling as CAS were included for analysis (*n*=64). Patient age, headache diagnosis, pain site, preventive use and CAS phenotype were collated and analysed in IBM SPSS 27. **Results:** Subjects were 72% female, age range 23-83 (median 49, IQR 21). Headache diagnoses were chronic migraine (50%), chronic cluster headache (11%), undifferentiated continuous lateralised headache (9%), SUNCT/SUNA (8%), hemicrania continua (8%), episodic migraine (8%), episodic cluster headache (3%) and trigeminal neuropathies (3%). Most (89%) described pain in the trigeminal distribution; 25% involving all three divisions and 67% including V3. Throat swelling was reported by 54, voice change by 17 and both by 7. Between 1-11 CAS were reported (median 6, IQR 3); the most common were lacrimation (*n*=47), facial swelling (*n*=45) and rhinorrhoea (*n*=37). There was significant agreement between the co-reporting of throat swelling (**Σ**21= 7.59, *P*=0.013) and voice change (**Σ**21= 6.49, *P*=0.02) with aural fullness. **Conclusion:** Voice change and throat swelling may be parasympathetically-mediated CAS. They may be co-associated and associated with aural fullness, suggesting a broadly somatotopic endophenotype.

## AL073 Safety and efficacy of occipital nerve stimulation for attack prevention in medically intractable chronic cluster headache (ICON): a randomised, double-blind, multicentre, phase 3, electrical dose-controlled trial

### P. Wilbrink^1^, I. de Coo^2^, P. Doesborg^2^, W. Mulleners^3^, O. Teernstra^4^, E. Bartels^2^, K. Burger^5^, F. Wille^6^, R. Dongen^7^, E. Kurt^8^, G. Spincemaille^4^, J. Haan^2^, E. Zwet^2^, F. Huygen^9^, M. Ferrari^2^

#### ^1^Zuyderland Medical Centre, Neurology, Heerlen, Netherlands; ^2^Leiden University Medical Center, Neurology, Leiden, Netherlands; ^3^Canisius Wilhelmina Hospital, Neurology, Nijmegen, Netherlands; ^4^Maastricht University Medical Centre, Maastricht, Netherlands; ^5^Alrijne Hospital, Anaesthesiology, Leiderdorp, Netherlands; ^6^Diakonessenhuis Hospital, Anaesthesiology, Zeist, Netherlands; ^7^Radboud University Medical Centre, Anaesthesiology, Nijmegen, Netherlands; ^8^Radboud University Medical Centre, Neurosurgery, Nijmegen, Netherlands; ^9^Erasmus University Medical Center, Anaesthesiology, Rotterdam, Netherlands

##### **Correspondence:** P. Wilbrink

**Background** Occipital nerve stimulation (ONS) has shown promising results in small uncontrolled trials in patients with medically intractable chronic cluster headache (MICCH). We aimed to establish whether ONS could serve as an effective treatment for such patients.

**Methods** The ONS in MICCH (ICON) study is a multicentre, randomised, double-blind, clinical trial. After 12 weeks baseline, patients were randomly allocated (1:1 ratio) to 24 weeks of ONS at either 100% or 30% of the individually determined range between paraesthesia threshold and near-discomfort (double-blind phase). In weeks 25–48, participants received individually optimised open-label ONS. The primary outcome was the weekly mean attack frequency in weeks 21–24 compared with baseline across all patients and, if a decrease was shown, to show a group-wise difference. (ClinicalTrials.gov NCT01151631).

**Findings** We enrolled 150 patients and randomised 131: 65 for 100% and 66 for 30% ONS. Median (IQR) weekly MAF in the total population was decreased to 7.38 (2.50, 18.50; p<0.0001) in weeks 21-24, a median change of -5.2 (IQR -11.18, -0.18; p<0.0001) attacks per week, without difference between treatment arms. In the blinded phase, there were 129 (100% ONS) and 95 (30% ONS) adverse events (AEs).

**Interpretation** In patients with MICCH ONS substantially reduced attack frequency and was safe. Future research should focus on optimising stimulation protocols and disentangling the underlying mechanism of action.

## AL074 Acute Treatment with Rimegepant 75 mg Confers Long Term Improvements in Median Time to 30% and 50% Reductions in Monthly Migraine Days – Post Hoc Results from an Open Label Safety Study (BHV3000-201)

### G. L'Italien^1^, E. Popoff^2^, K. Johnston^2^, D. McGrath^1^, C. M. Conway^1^, L. Powell^2^, L. Harris^1^, N. Kowalczyk^1^, R. Croop^1^, V. Coric^1^

#### ^1^Biohaven Pharmaceuticals, New Haven, CT, United States; ^2^Broadstreet Health Economics & Outcomes Research, Vancouver, Canada

##### **Correspondence:** E. Popoff

**OBJECTIVE:** The objective of this study was to describe long-term reductions in monthly migraine days (MMD) associated with rimegepant 75 mg oral tablet, when taken as needed (PRN) as an acute treatment.

**METHODS:** Eligible subjects were a subset of the BHV3000-201 trial (NCT03266588): adults with ≥1 year migraine history and ≥6 MMD at baseline, treated with rimegepant 75 mg PRN up to once daily, for up to 52 weeks. Kaplan-Meier analyses were used to assess median time to ≥30% and ≥50% reduction in MMDs from baseline. Cluster analyses were used to identify 3 clusters based on MMD over the study period.

**RESULTS:** Among 1,044 subjects, the median time to ≥30% reduction in MMDs was 12 weeks (IQR; 4-40 weeks); median time to ≥50% reduction was 32 weeks (IQR; 12-NR weeks). MMD reduction was observed over time regardless of baseline migraine frequency, including low-frequency (baseline MMD 8.7), moderate frequency (baseline MMD 11.5) and high-frequency (baseline MMD 14.8) cluster groups (Figure 1). Higher baseline MMD were associated with a longer time to achieving ≥30% or ≥50% MMD reduction.

**CONCLUSIONS:** In subjects presenting with ≥6 MMD, PRN acute treatment over 52 weeks with oral rimegepant 75 mg conferred clinically significant migraine reductions. There was no evidence of medication-related MMD increases with long-term PRN rimegepant use. These findings are consistent with preventive benefits of rimegepant 75 mg shown in a placebo-controlled study.

**Fig. 1 (Abstract AL074).**
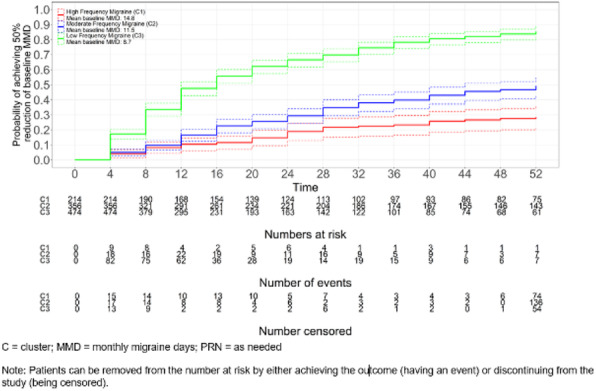
Time to ≥50% reduction in MMDs from baseline by mean baseline MMD group

## AL076 Efficacy Results from an Open-Label Safety Study of Galcanezumab in Patients with Episodic or Chronic Cluster Headache

### C. Gaul^1^, R. Riesenberg^2^, C. Stroud^3^, Y. Dong^3^, T. Myers Oakes^3^

#### ^1^Headache Center, Frankfurt a. M., Germany; ^2^Atlanta Center for Medical Research, Atlanta, GA, United States; ^3^Eli Lilly and Company, Indianapolis, IN, United States

##### **Correspondence:** C. Gaul, R. Riesenberg

**Objective** To evaluate the effect of galcanezumab (GMB) on 2 patient reported outcomes in patients with cluster headache (CH).

**Methods** This was a Phase 3b multicenter, single-arm, open-label safety study, open only to individuals who completed one of the parent Phase 3 studies (CGAM [chronic CH] or CGAL [episodic CH]). Participants received GMB 300mg subcutaneously up to once monthly. Efficacy-related objectives assessed the effect of GMB on the PGI-I, the EQ-5D-5L Health State Index scores and EQ-VAS Current Health score. Spearman correlation coefficients between PGI-1 and EQ-5D-5L scores mean change from baseline at 1 month (m) were also calculated.

**Results** Of the 165 participants enrolled, 164 received at least 1 dose of GMB and 163 had ≥ 1 post baseline assessment. Findings are summarized in the table. Majority of participants (≥80%) reported their CH status as very much/much/a little better in PGI-I at 1, 6, and 12 m. The US Health State Index and VAS generally improved from baseline to 1, 6, and 12m. Correlations between mean change in PGI-I and mean change value in EQ-5D-5L Health State Index Scores and VAS Current Health score at 1m were associated and ranged from -0.28 to -0.37.

**Conclusion** Majority of participants reported their CH condition to be very much/much/a little better, as measured using the PGI-I, at 1, 6, and 12m. EQ-5D-5L Health State Index scores (US and UK) and the EQ-VAS Current Health score improved from baseline to 1,6, and 12m.

**Fig. 1 (abstract AL076).**
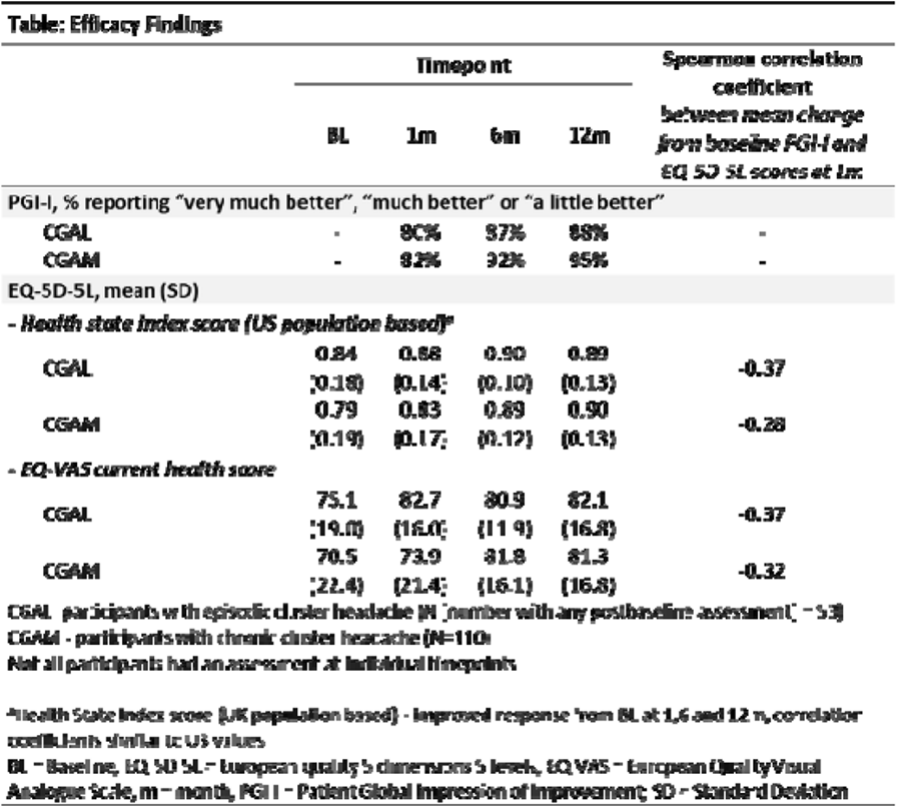
See text for description

## AL077 Evaluation of the long-term safety and tolerability of oral atogepant 60 mg once daily for preventive treatment of migraine: a phase 3, 40-week, multicenter extension to the advance trial

### B. Klein^1^, R. Miceli^2^, L. Severt^2^, P. McAllister^3^, L. Mechtler^4^, J. McVige^4^, M. Diamond^5^, M. J. Marmura^6^, H. Guo^2^, M. Finnegan^2^, J. M. Trugman^2^

#### ^1^Abington Neurological Associates, Ltd., Abington, PA, United States; ^2^AbbVie, Madison, NJ, United States; ^3^New England Institute for Neurology & Headache, Stamford, CT, United States; ^4^DENT Neurologic Institute, Amherst, NY, United States; ^5^Diamond Headache Clinic, Chicago, IL, United States; ^6^Thomas Jefferson University, Department of Neurology, Philadelphia, PA, United States

##### **Correspondence:** L. Mechtler

**Background and Objective:** A phase 3 trial, ADVANCE (NCT03777059), demonstrated that atogepant, an oral, CGRP receptor antagonist dosed once daily, results in a clinically meaningful reduction in mean monthly migraine days. This open-label extension for ADVANCE trial completers evaluated the long-term safety and tolerability of atogepant over 40 weeks.

**Methods:** Participants in this trial (NCT03939312) rolled over from the lead-in ADVANCE trial and were treated with atogepant 60 mg once daily for 40-weeks, with a 4-week safety follow-up period. Only safety data were collected.

**Results:** 685 participants took at least one dose of study drug, 74.6% completed the 40-week treatment period; mean age of 41.8 years, 88.2% female, 84.4% white, and mean BMI of 30.58 kg/m2. Mean (SD) treatment duration was 233.6 (89.32) days. Overall, 62.5% of participants experienced a treatment-emergent adverse event (TEAE), with 8.8% considered treatment-related by the investigator; serious adverse events (SAEs) occurred in 3.4% of participants, none were treatment-related. Table 1 reports the most frequent AEs leading to discontinuation; Table 2 reports the most frequent TEAEs observed. No deaths and no hepatic safety issues were observed.

**Conclusion:** These safety results are consistent with the known safety profile of atogepant from previous trials and support the long-term safety and tolerability of once daily dosing of atogepant 60 mg.

**Fig. 1 (abstract AL077).**
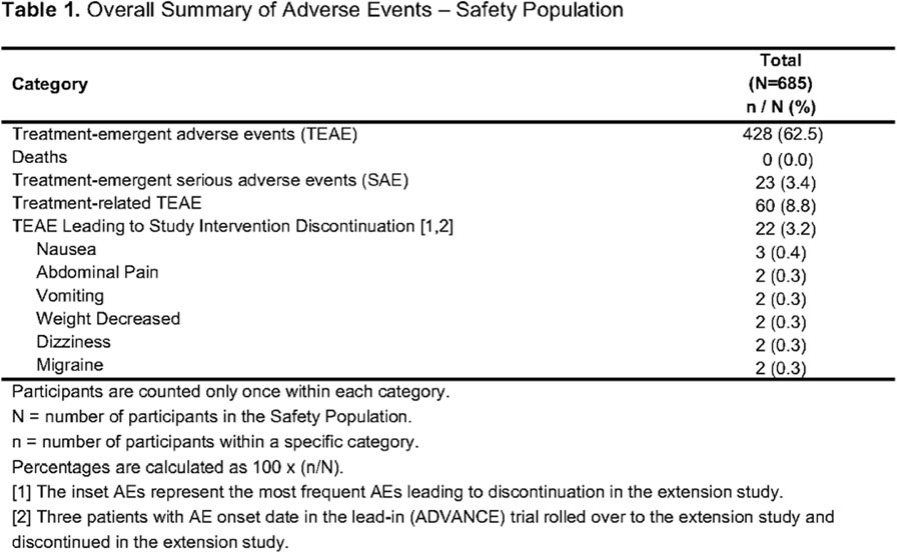
See text for description

**Fig. 2 (abstract AL077).**
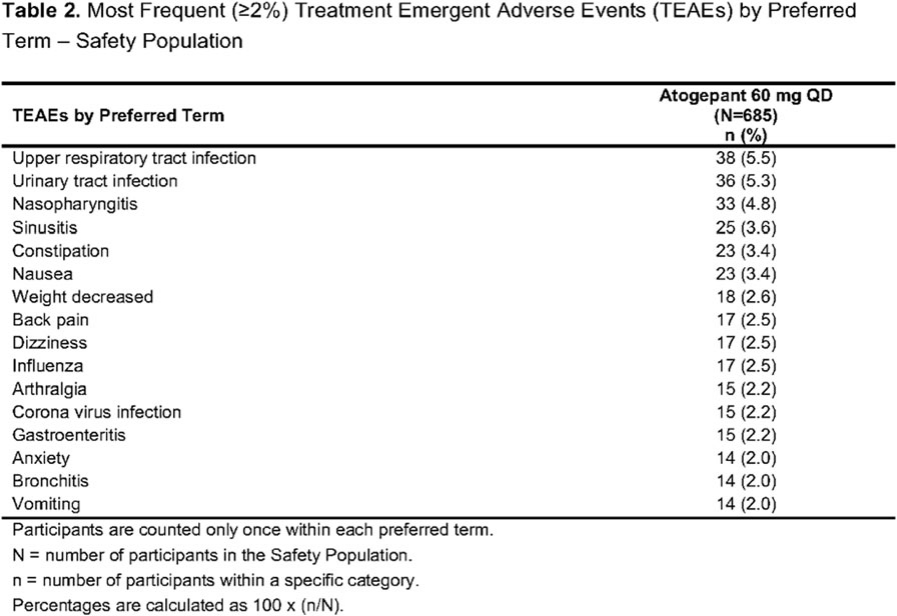
See text for description

## AL078 Real-world effectiveness data of erenumab-treated migraine patients in Switzerland: The SQUARE study

### E. Schäfer^1^, M. Arzt^1^, I. Meyer^1^, A. Gantenbein^2^

#### ^1^Novartis Pharma Schweiz AG, Neuroscience, Rotkreuz, Switzerland; ^2^Zurzach Care, Neurology, Bad Zurzach, Switzerland

##### **Correspondence:** A. Gantenbein

**Objective:**

To present real-world effectiveness data of erenumab from the SQUARE (Swiss QUality of life and healthcare impact Assessment in a Real-world Erenumab treated migraine population) study.

**Methods:**

SQUARE is a non-interventional study which provides clinical effectiveness data on erenumab in a post-marketing setting from both migraine care specialist centers and general neurologists in Switzerland. Migraine patients receiving Aimovig® in accordance with the Swiss label were included if willing and able to participate, with the exception of patients with prior use of any medications targeting the calcitonin gene-related peptide (CGRP) pathway or recent use of investigational drugs. Patients were observed over a period of 24 months.

**Results:**

A total of 173 adult patients were included in 19 centers. Here, we will present real-world effectiveness data of erenumab at month 6 compared to baseline, including scores of Headache Impact Test (HIT-6), modified (monthly) migraine disability assessment test (mMIDAS), and impact of migraine on partners and adolescent children (IMPAC).

**Conclusion:**

These are among the first prospectively collected data on erenumab under routine medical care. Results from the SQUARE study will provide insights into the effectiveness of a CGRP receptor-based therapy in a real-world scenario.

## P0405 An atypical case of Epicrania fugax with coronal pain radiation

### A. Cordeiro, E. Silva, M. Grunho, L. Pereira

#### Hospital Garcia de Orta, Department of Neurology, Almada, Portugal

##### **Correspondence:** A. Cordeiro

Objective: Epicrania fugax (EF) is a primary headache consisting of brief stabbing head pain, following a linear or zigzag trajectory across the scalp, through the territories of different nerves. Although rarely, some cases of coronal radiation of pain have been described.

Methods: Clinical case.

Results: A 48-year-old woman with a history of migraine without aura presented to the emergency department with new onset headache: stabbing, severe (10/10 on the visual analog scale), describing a linear trajectory on the coronal plane from the right temporal to the left temporal scalp, lasting 1-40 seconds, multiple times a day, preventing sleep. Neurological examination revealed right hemicrania hypoesthesia. Laboratory tests were unremarkable, and brain magnetic resonance imaging exhibited dilated Virchow-Robin perivascular spaces (PVS) in the left hemi-midbrain. Pregabalin 25mg twice a day was started with immediate pain relief and complete resolution by the sixth day.

Conclusion: We believe this to be a case of atypical EF with coronal radiation, raising awareness that patients can present with linear pain of different trajectories across the scalp. Despite the presence of dilated PVS, they are unlike to be causal due to their nature, lateralization and absent relation with the trigeminal nucleus and other relevant pain matrix structures. Early diagnosis of these atypical cases is essential to provide the proper treatment.

## P0406 Clustering personality traits in chronic cluster headache patients: a data driven approach

### A. Telesca^1^, A. Proietti Cecchini^1^, M. Consonni^1^, S. Piacentini^1^, S. Usai^1^, G. Lauria Pinter^1,2^, L. Grazzi^1^

#### ^1^IRCCS Foundation “Carlo Besta” Neurological Institute, Neuroalgology, Milan, Italy; ^2^University of Milan, Biomedical and Clinical Sciences “Luigi Sacco”, Milan, Italy

##### **Correspondence:** A. Telesca

Chronic Cluster headache (CH) patients are usually comorbid to mood spectrum disorders, psychopathological symptoms and personality disorders, but the role of psychopathological aspects is still insufficiently explored. Our aim is to verify, by a data driven approach, if CH patients may be classified on the basis of personality trait disorders. We applied hierarchical cluster analysis (HCA) to classify 60 patients suffering from CH, based on values of the clinical personality pattern scales of the Millon Clinical Multiaxial Inventory-III (MCMI-III). Subgroup comparison on demographical data and clinical features where subsequently performed. The outcome of HCA revealed the existence of 3 groups of patients with different personality traits. Two groups had unique patterns. Group 1 (n = 20) had distinctive **avoidant**, **borderline** and **schizotypal** personality traits. Group 2 (n = 15) scored high on the **obsessive-compulsive** and **histrionic** personality traits. Group 3 (n = 25) did not show relevant patterns. Clinically, Group 2 had lower education level and higher level of **post-traumatic stress symptoms** and **dysthymia** than the other groups. Our data-driven approach revealed different personality profiles in chronic CH with specific psychopathological features. This might help to identify the most appropriate therapeutic strategies and predict the evolution of the disease.

## P0407 SUNA syndrome: a case of transformation from trigeminal neuralgia

### I. Velichko, M. Barabanova, G. Muzlaev

#### Kuban State Medical University, Neurology and Neurosurgery, Krasnodar, Russian Federation

##### **Correspondence:** I. Velichko

**Background and objective**: Short-lasting unilateral neuralgiform headache attacks with cranial autonomic symptoms (SUNA) is a trigeminal autonomic cephalalgia presenting as unilateral periorbital pain with autonomic symptoms. We are reporting a patient with SUNA syndrome who transformed from trigeminal neuralgia.

**Methods**: Case report.

**Results**: The 33-year-old man with no medical history or family history of headache first became ill 2 years ago. Initially, there were attacks of severe stabbing pain as "electric shock" in the right supraorbital and zygomatic areas, both spontaneously and caused by light touch in the same areas. Attacks lasting 10-15 sec occurred 3-10 times a day. Idiopathic trigeminal neuralgia was diagnosed by a neurologist, and carbamazepine significantly reduced the pain. After 8 months, the attacks recurred; the pain was strictly in the right orbital periorbital area, lasting 60-120 sec and a frequency of about 12-13 per day. The attacks were accompanied by intense unilateral сonjunctival injection. There was moderate interictal pain. Clinical and diagnostic examinations did not reveal any abnormalities. Carbamazepine, gabapentin, topiramate were not beneficial. Lamotrigine (100 mg/day) significantly reduced the frequency and intensity of the pain.

**Conclusion**: This observation suggests that SUNA is probably more closely linked to trigeminal neuralgia than to other unilateral headache syndromes.

**Disclosure of Interest**: The authors declare no conflicts of interest.

## P0408 Safety and Tolerability Findings from an Open-Label Safety Study of Galcanezumab in Patients with Episodic or Chronic Cluster Headache

### R. Riesenberg^1^, C. Gaul^2^, C. Stroud^3^, T. Myers Oakes^3^, Y. Dong^3^, M. Bangs^3^, R. Wenzel^3^, J. Martinez^3^

#### ^1^Atlanta Center for Medical Research, Atlanta, GA, United States; ^2^Headache Center, Frankfurt a. M., Germany; ^3^Eli Lilly and Company, Indianapolis, IN, United States

##### **Correspondence:** R. Riesenberg

**Objective** To evaluate the safety of open-label galcanezumab (GMB) within the context of medical practice in eligible participants with cluster headache (CH).

**Methods** This was a Phase 3b multicenter, single-arm, open-label safety study, for individuals who completed one of the parent Phase 3 studies (CGAM [chronic CH] or CGAL [episodic CH]). Participants received GMB 300mg subcutaneously up to once monthly. Safety was assessed through serious adverse events (AEs), treatment-emergent AEs (TEAEs), suicidality and immunogenicity findings.

**Results** Of the 183 participants who entered the study, 165 enrolled of whom 164 received at least 1 dose of GMB. The majority were male (75%) and white (85%); the mean age was 48 years (range 23-66). The most common reasons for study discontinuation were study termination by the sponsor (71%), lack of efficacy (12%), and withdrawal by the participant (9%). The mean duration of exposure to GMB 300 mg was 475 days (range 28-1211). AE findings are summarized in the table. Two participants (1.2%) experienced suicidal ideation but not suicidal behavior; one occurred during the GMB-treatment time, the other while off treatment. One patient died by suicide, not related to study treatment.

**Conclusion** GMB 300 mg was well tolerated. The safety profile of GMB 300 mg in this study was consistent with that observed in previous Phase 3 studies.

**Fig. 1 (abstract P0408).**
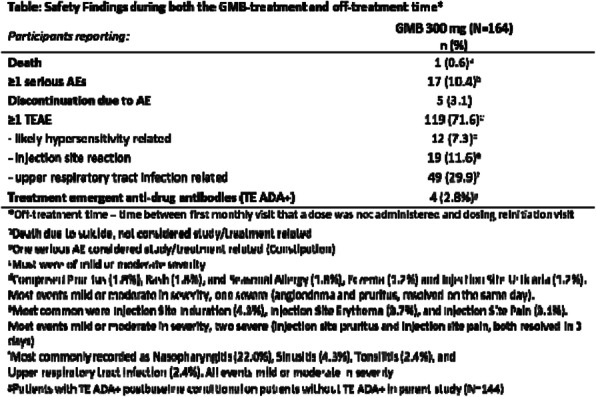
See text for description

## P0409 Exploratory study of the effectiveness of Oxygen Therapy Using Home Oxygen Concentrators in the acute treatment of cluster headache

### D. W. Bae^1^, S. J. Cho^2^, S. H. Cho^3^, H. S. Moon^4^, M. K. Chu^5^, B. K. Kim^6^

#### ^1^St. Vincent's Hospital, Neurology, Suwon, South Korea; ^2^Dongtan Sacred Heart Hospital, Neurology, Hwaseong, South Korea; ^3^Uijeongbu Eulji Medical Center, Neurology, Uijeongbu, South Korea; ^4^Kangbuk Samsung Hospital, Neurology, Seoul, South Korea; ^5^Severance Hospital, Neurology, Seoul, South Korea; ^6^Nowon Eulji Medical Center, Neurology, Seoul, South Korea

##### **Correspondence:** D. W. Bae

**Background & Objective:** Oxygen therapy is the established acute treatment of cluster headache, but there are many obstacles that prevent widespread use by patients. Oxygen therapy using cylinder needs continuous replacement of oxygen and some difficulties in storage and delivery. Oxygen concentrator supplied oxygen at concentration of more than 90% and at flow rates up to 6L/min. We report four patients with episodic cluster headache who were treated with oxygen therapy from one or two oxygen concentrators.

**Methods:** Four patients with cluster headache were enrolled during their cluster period. There were three treatment protocols; 1) oral abortive medication 2) two oxygen concentrators connected to non-rebreathing mask using Y-tube 3) one oxygen concentrator connected to non-rebreathing mask. Four enrolled patients documented total 50 treated attacks (22 attacks treated with oral medication, 20 attacks treated with two oxygen concentrator, 8 attacks treated with one oxygen concentrator). Among the three treatment options, two oxygen concentrator showed superior pain free response rate at 30 minutes (65%) compared to one oxygen concentrator (62.5%) and oral medication (4.5%). Attack duration in two oxygen concentrators was also shortest among the three treatment protocols(29±1.4min).

**Conclusion:** This study is going to provide the evidence of oxygen concentrator therapy in the acute treatment of cluster headache. Further randomized controlled trial is in progress.

**Fig. 1 (abstract P0409).**
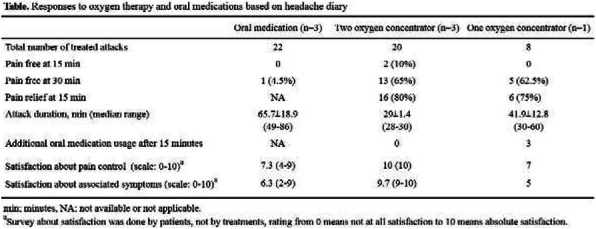
See text for description

## P0410 Association study between headache and dizziness according to age spectrum for patients

### J. Lee

#### BuCheon U-RI General Hospital, Seoul, Korea, Department of Neurology, Bucheon-si, South Korea

**Background and objective:** We investigate whether vertigo is correlated with clinical relevance in headache according to age spectrum.

**Methods:** From May 2020 to February 2021, 925 patients, aged ≥ 7 years and participants who met the inclusion criteria were enrolled from the neurology clinics. We re-analyzed patients who experience headache with/without vertigo symptoms and two groups were studied according to the patient"s age. Logistic regression analysis was used to evaluate the association between headache and vertigo. First, we reviewed vertigo with diagnosed 281 and 570 headache patients, and 31 vertiginous headache. Second, common types of primary headache patients were included. Third, the vertigo was classified into 2 groups: peripheral or central types.

**Results**: Of the 925 outpatient, headache of 570(61.6%) and vertigo of 281(30.3%) were reported. In this study, prevalence of headache of 200(35.1%) in men and 370(64.9%) in women was expected. The frequency of vertigo in headache patients was significant relationship with ageing{Ages 0 to 9:0, 10 to 19:2, 20 to 29:5, 30 to 39:2, 40 to 49:3, 50 to 59:8, 60 to 69:7, 70 to 79:1,80 to 89:3}. Total 31 headache related vertigo patients were divided into two groups{peripheral(n=27), central(n=4) type} and proportion of peripheral vertigo has a positive relevance with headache.

**Conclusions:** The findings of our study may provide direction for useful diagnostic and therapeutic approach to the vertigo attack in headache patients.

## P0411 Feasibility and effectiveness of home-withdrawal program combined with behavioural approach in patients with Chronic Migraine & Medication Overuse during Covid-19 emergency (BE-Home)

### L. Grazzi^1^, D. D'Amico^1^, A. Telesca^1^, P. Rizzoli^2^

#### ^1^IRCCS Foundation “Carlo Besta” Neurological Institute, Neuroalgology, Milan, Italy; ^2^Harvard Medical School, J Graham Headache Center, Brigham & Faulkner Hospital, Boston, MA, United States

##### **Correspondence:** L. Grazzi

Chronic Migraine (CM)is a disabling condition that affects the 2% of migraine population often complicated by Medication overuse (MO) and withdrawal is often needed for these patients. Clinical results can be improved when traditional therapies are combined with behavioral approaches (mindfulness). As the emergency situation due to the COVID-19 pandemic, the regular clinical practice adopted for patients with CM-MO changed and the traditional program for withdrawal has been modified. We run a pilot study to enforce the application of a Home- withdrawal procedure and the use of web technology so that patients could continue their therapeutic process by using behavioral support (mindfulness). We enrolled 30 CM-MO patients. A withdrawal program at home, by oral administration of therapies, with specific instructions and education, was organized. Instructions for behavioral approach (mindfulness) were given: daily 12-minutes standardized mindfulness sessions on their smartphone have been combined with weekly 60-minutes-video-on line-sessions for 6 weeks. A face-to-face follow-up was scheduled every 3 months until 12 months. 15 patients achieved the 3 months follow-up: days of migraine/month, medication intake/month, catastrophizing attitude decreased significantly (PCS: 21.5±6.7 vs 9+8.7; 22.1±5.7 vs 7.9±8.6; 28±11.4 vs 21±10.9). Results seem to encourage toward the application of this approach in emergency condition and in regular clinical practice too.

## P0412 Effectiveness of MINDFULNESS by smartphone, for patients with Chronic Migraine and Medication Overuse during the Covid-19 emergency

### L. Grazzi^1^, A. Telesca^1^, P. Rizzoli^2^

#### ^1^IRCCS Foundation “Carlo Besta” Neurological Institute, Neuroalgology, Milan, Italy; ^2^Harvard Medical School, J Graham Headache Center, Brigham & Faulkner Hospital, Boston, MA, United States

##### **Correspondence:** L. Grazzi

Chronic Migraine is frequently associated with medication overuse that makes this condition difficult to treat. Prior investigations confirmed the efficacy of withdrawal, and clinical results are enhanced when traditional therapies are combined with behavioral approaches, in particular mindfulness. At the Besta Institute, after a medication withdrawal, patients follow a specific prophylaxis for migraine, and a program for mindfulness practice, administered for 6 weekly-45 minutes sessions in small groups. During the COVID-19 pandemic, technology (smartphones) has been used to treat patients practicing the mindfulness approach. **25** patients completing the withdrawal program were provided the additional training needed for mindfulness on their smartphone, practicing 12minutes/day. All remote sessions were recorded by the expert who generally managed face-to-face sessions at the hospital. A separate weekly video call was made to evaluate clinical condition, encourage and reinforce the use of pain management strategies. Follow-up at 3,6 and 12 months was scheduled. Fifteen patients achieved the 6 months follow-up: a decrease of migraine days/month and of medication intake/month are reported (CPS:20.6±6.1 vs 8.7±4.5; 19.4±5.8 vs 7.5±5.0). Our findings suggest this type of combined treatment can yield significant clinical results. Adherence to the treatment was high. This approach which evolved in response to a severe health crisis, warrants further, more controlled investigations.

## P0413 Headache symptom and COVID-19 survival: a systematic review and meta-analysis of 43,169 patients [VJGL1] [VJGL1]Max. 1500 characters with spaces

### V. Gallardo López^1^, R. E. Shapiro^2^, E. Caronna^1^, P. Pozo-Rosich^1^

#### ^1^VHIR, Neuroscience, Barcelona, Spain; ^2^University of Vermont, Department of Neurological Sciences, Burlington, United States

##### **Correspondence:** V. Gallardo López

**Objective:** To study the prevalence of headache as a prognostic factor for mortality in COVID-19 inpatients.

**Background**: COVID-19 has not impacted people uniformly. This disparity has prompted investigations to identify clinical and genetic predictors of COVID-19 mortality. Headache, a COVID-19 symptom, has been associated with positive disease prognosis.

**Methods:** Following PRISMA guidelines, we conducted a systematic literature search (April 1 to December 22, 2020) of COVID-19 clinical inpatients series that reported headache as a symptom of the disease. Random-effects pooling models were computed in order to estimate the effect size of the presence of headache in survived vs. non-survived COVID-19 cohorts. Quality of studies was assessed with Newcastle-Ottawa Scale (NOS). PROSPERO registration number: CRD42021260151.

**Results:** From a total of 48 full-text peer-reviewed meta-analyzed publications, the estimated prevalence of headache as a symptom among COVID-19 inpatients was 10.4% [8.3% - 12.9%]. We observed higher risk ratio (RR) of headache among COVID-19 inpatients who survived, compared to those who did not (RR: 1.90 [1.46-2.47], p<0.0001). In sensitivity analyses, excluding studies with lower quality (NOS score < 7), headache RR increased without a statistically significant heterogeneity between studies (RR: 2.60 [2.03-3.32], p<0.0001; I2=23.6%, p=0.180).

**Conclusion:** Headache as a COVID-19 symptom is associated with enhanced survival in hospitalized patients.

## P0414 Mask and Personal Protective Equipment (PPE) Associated Headache Among the COVID-Time Physicians of Bangladesh

### K. M. N. Joy^1^, R. Mahmud^2^, G. Rabbani^3^, M. K. Islam^4^, M. I. Khalil^1^, N. C. Kundu^1^

#### ^1^Shaheed Suhrawardy Medical College, Department of Neurology, Dhaka, Bangladesh; ^2^Dhaka Medical College Hospital, Department of Neurology, Dhaka, Bangladesh; ^3^University of Dhaka, Department of Statistics, Dhaka, Bangladesh; ^4^Dhaka Medical College Hospital, Department of Medicine, Dhaka, Bangladesh

##### **Correspondence:** K. M. N. Joy

**Objective:** We investigated the prevalence of mask/PPE associated headache among Bangladeshi physicians during COVID 19 pandemic along with the risk factors and headache characteristics. Headache severity was assessed by the Headache Impact Test (HIT-6).

**Methods:** This is a cross-sectional, online Google form based study among 200 physicians from different hospitals in Dhaka conducted from December 2020 to April 2021.

**Results:** Majority participants were male (129, 64.5%) with mean (SD) age of 35.4(7.5) years. Filter masks (146, 73%) were mostly used along with other PPE (139, 69.5%). Headache prevalence was 71% and 59.9% developed new onset headache. Doctors working in the COVID unit [OR: 2.47, 95% CI: 1.18-5.18; *P*=0.017] had the highest risk of developing headache. Headache occurrence was independently associated with previous primary headache [OR:5.40, 95% CI: 2.03-14.41; *P*=0.001] and combined mask & all PPE usage [OR:2.48,95%CI:1.31-4.23 ;*P*=0.006] for ≥ 6months [OR:2.05, 95%CI:1.05-3.99; *P*=0.036].Most headaches were dull aching (33.8%), lasted for 1-4 hours (58.5%) & relieved within 1 hour of mask removal (43.6%). Headache Impact Test (HIT-6) score was substantial to severe among the doctors with previous headaches [OR: 2.91, 95%CI:1.43-5.92; *P*=0.003] and those having moderate to severe stress levels[OR: 2.56, 95%CI: 1.19-5.55; *P*=0.017].

**Conclusions:** Most physicians with previous primary headache develop mask/PPE associated headache with considerable impacts on daily life.

**Fig. 1 (abstract P0414).**
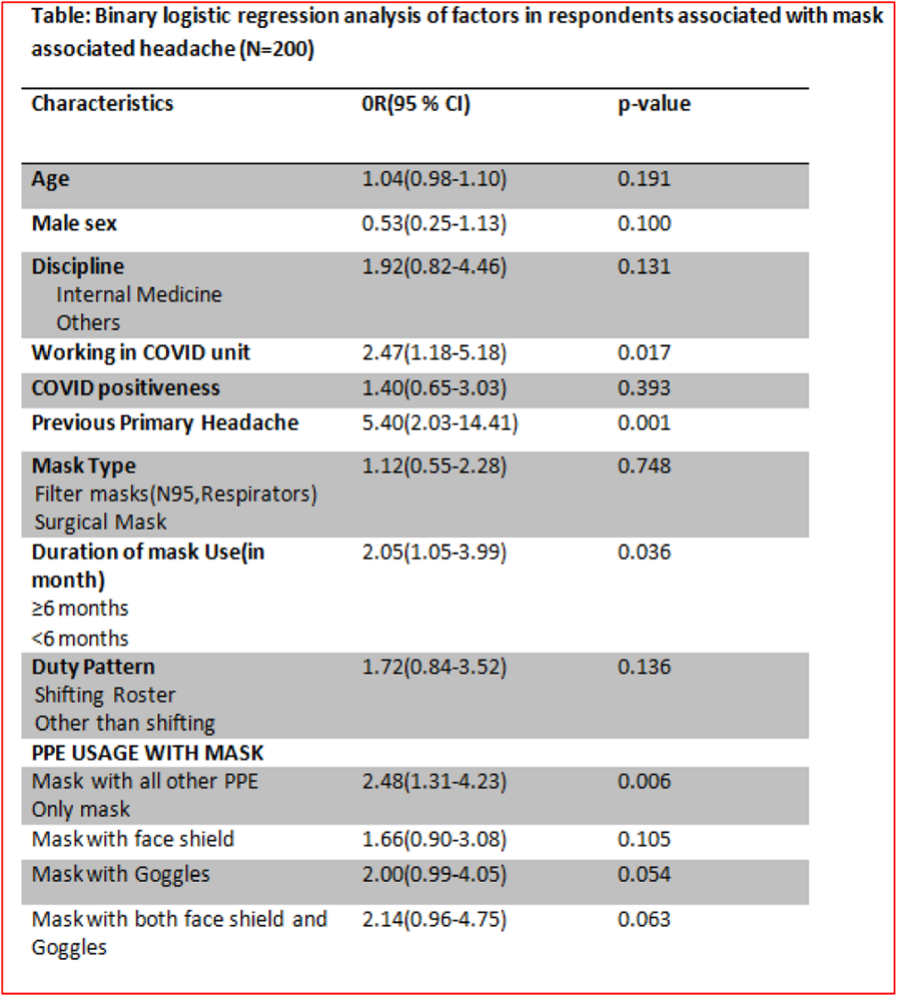
See text for description

**Fig. 2 (abstract P0414).**
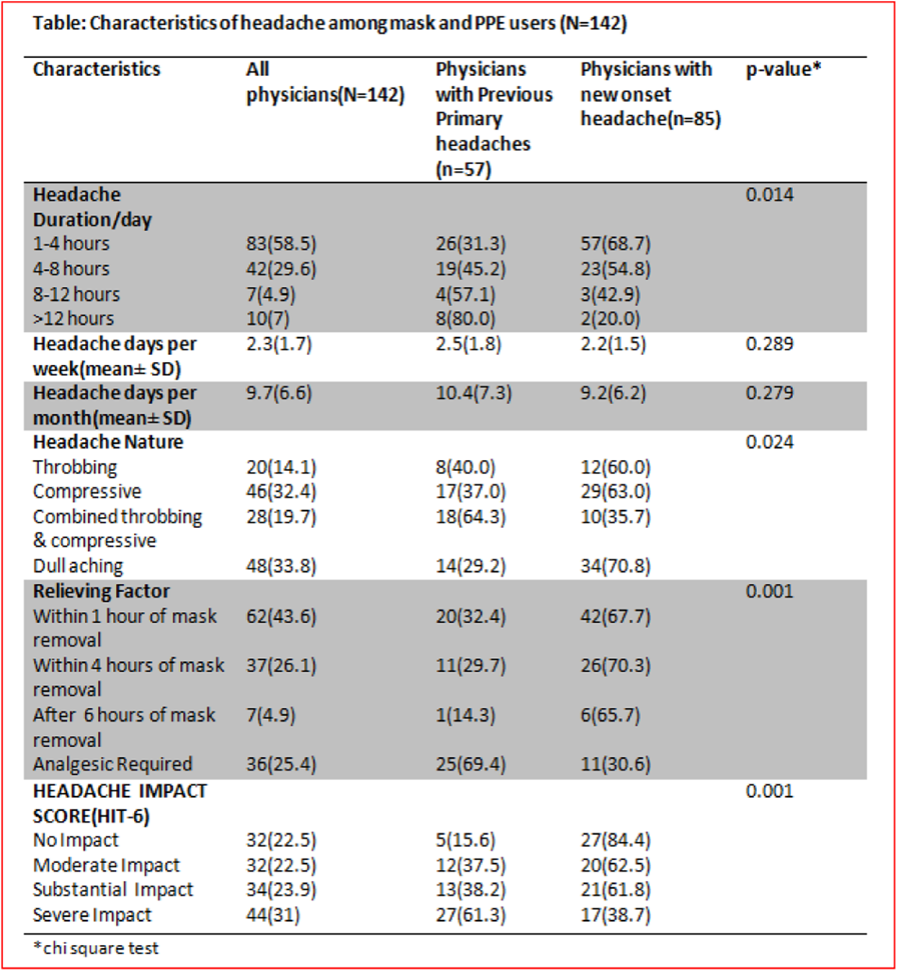
See text for description

## P0415 Headache during COVID-19 and Post COVID-19 Headache: Observations in the largest COVID-19 center in México

### R. A. Garcia Santos, M. S. Rodríguez Rodríguez, A. S. Ramírez García Luna, D. S. López Gonzalez

#### National Institute of Respiratory Diseases, Neurology, Mexico City, Mexico

##### **Correspondence:** R. A. Garcia Santos

Our objective is to report headache during and post COVID-19 in hospitalized COVID-19 survivors.

Three neurologists examined and analyzed the presence of headache on 273 patients admitted at a single medical center of COVID-19 three months after their hospital discharge, in Mexico City, from November 1st 2021 to June 1st 2021. All patients were positive for SARS-CoV-2 RT-PCR.

Most of the patients were men (63.4%). 65.2% intubated with mean intubation of 19 days. 49.5% experienced headache during COVID-19, being holocranial (36.3%) and oppressive. Duration ranged from 1 to 5 days, (mean of 3 days). Patients with headache during COVID-19, had a higher frequency of post COVID-19 fatigue (85.2%), this difference was statistically significant among groups (p= 0.02). Cognitive complain at the time of neurological evaluation was frequent in patients with headache during COVID-19 (71.1%).

41.8% patients experienced post COVID-19 headaches, holocranial oppressive headache was the most common (27.2%) with a frequency of twice a week in 28.3%. Most patients with post COVID-19 had headache during COVID-19 (73.3%), this difference was statistically significant among groups (p= <0.001). Patients who presented with post COVID-19 headache, had a higher frequency of post COVID-19 fatigue (74.2%), with statistically significance (p= 0.01). Almost half of the patients presented depressive symptoms at the time of evaluation, with no difference among headache groups regarding these symptoms.

## P0416 Prevalence of primary headaches in a group of hospital workers during Sars-COV2 infection

### E. Pucci^1,2,3^, M. Q. Falvo^1,2,3^, V. Nava^1,2,3^, M. Lagorio^1,2,3^

#### ^1^Università di Pavia, Department of Brain and Behavioral Sciences, Pavia, Italy; ^2^University Of Pavia, Graduate School of Geriatrics and Gerontology, Pavia, Italy; ^3^ASP – IDR S. Margherita, Alzheimer’s Nucleus, Pavia, Italy

##### **Correspondence:** E. Pucci

**Background and objective**

Headaches represent at the same time the symptom and the disease, while the secondary ones are the expression of an ongoing pathology that can be systemic. The aim of this study is to determine the prevalence rate in the workplace in a ward during the period of Sars-COV2 infection. This survey was carried out using 2 questionnaires: work activity and headache sheet according to IHS criteria.

**Methods**

All health personnel belonging to the Alzheimer Nucleus of the IDR S. Margherita di Pavia were subjected to compilation of questionnaires during the Sars-COV2 infection period.

**Results**

From the analysis of the questionnaires administered, it was found that out of 15 workers, 10 women and 5 men. 4 (all women had migraines without aura) and 7 tension-type headaches (5 women and 2 men). Before the Sars-COV2 period, only 2 workers had migraine without aura and 2 tension-type headaches (all women). All 11 workers reported stress, insomnia, and concern for family members and their own health. None of the workers at the time of testing had been vaccinated.

**Conclusions**

Factors related to the work environment are able to increase the frequency and/or intensity of pre-existing headaches. It is also likely that particular situations can give rise to or cause some forms of headache under certain working conditions. Excessive responsibility or, on the contrary, disaffection and incongruous work rhythms should be considered among the occupational risk factors.

## P0417 Atypical migraine: sentinel symptom for Sars-COV2 infection

### E. Pucci^1,2,3^, M. Q. Falvo^1,2,3^, V. Nava^1,2,3^, M. Lagorio^1,2,3^

#### ^1^Università di Pavia, Department of Brain and Behavioral Sciences, Pavia, Italy; ^2^University Of Pavia, Graduate School of Geriatrics and Gerontology, Pavia, Italy; ^3^ASP – IDR S. Margherita, Alzheimer’s Nucleus, Pavia, Italy

##### **Correspondence:** E. Pucci

**Background and objective**

Migraine without aura is the most frequent of the forms of migraines (about 60 - 80% of all forms of migraines). There are many causes that can trigger migraines, including infections (IHS ICDH-3).

**Methods**

69 year old woman. Professional nurse. Family history of migraine (maternal line). Arising in school age. Diagnosis made according to the IHS ICDH-3 criteria. The patient presented 2-3 crises / month with pulsating pain in the bilateral frontotemporal region, medium-strong intensity, associated with photo-phonophobia, nausea, sometimes vomiting. Duration 24-36 hours. Triggering factors: menstruation and psychophysical stress. After menopause (49 years) reduction of intensity, duration and frequency with 1-2 cris / month related stress lasting 12-24 hours and responsive to NSAID intake. No preventive therapy performed.

**Results**

On 29.11.2020 episode of atypical headache (described as different from other episodes) with very strong, throbbing, stabbing, burning pain in the bilateral front-temporal region. Duration 24 hours. No other symptoms reported, apiretic. 30.11.2020: TNF fast: +. Molecular TNF: positive for SARS COV 2. During the period of infection headache present whenever the patient had fever and was unresponsive to paracetamol.

**Conclusions**

In our case report, atypical migraine can be considered a sentinel symptom of an initial infection. The patient works as a professional nurse in the ward which had become Covid on 3.11.2020.

## P0418 Headache and covid 19: moldovan online survey

### O. Grosu

#### Diomid Gherman Institute of Neurology and Neurosurgery, Headache center, Chisinau, Moldova

**Objective:** The aim of the study was to characterize the Moldovan cohort of headache and COVID 19 infection patients.

**Methods:** A survey of people with COVID 19 and headaches from January till June 2021 was done. The study sample consists of 138 volunteers, mean age 36.74±8.16, 89.6% female, that complete online a questionnaire about demographics, comorbidities, clinical signs of COVID 19 infection, headache before, during, and after COVID 19, screening for anxiety, depression, and sleep disorders.

**Results:** the study showed that 58.3% of the respondents had different forms of headache before COVID 19 infection and 17.9% severe forms. During the period of COVID 19 infection - 91% of respondents had bouts of headache attributed to COVID 19 infection and 54.5% severe forms with increased intensity, generalized localization (30.9%), associated with vertigo (64.8%), nausea (54.1%), peripheral vegetative signs (22.4%), accompanied with pronounced asthenia (80%) and pain with another localization (85.5%). Persistent headaches after COVID presented 62.7% of respondents, being severe for 16.7% of them with associated vertigo (34.7%), nausea (25.2%), and phonophobia (39.9%).

**Conclusion:** The percentage of patients with a headache in the post-COVID period is worryingly high, it increases the degree of functional disability of patients and the individual and social burden, respectively.

## P0419 Medication overuse in patients with headache and covid 19 infections

### O. Grosu

#### Diomid Gherman Institute of Neurology and Neurosurgery, Headache center, Chisinau, Moldova

**Background:** Medication overuse in patients with headaches is the most important risk factor for chronification and persistence. **The aim** of the study was to analyze the use of analgesics in patients with headaches and COVID 19 infection and evaluate medication overuse according to international criteria.

**Methods:** An online survey, launched through social media channels from January till June 2021, was completed by patients with headaches and COVID 19 disease. Validated questionnaire gathered data on demographics, COVID infection, the characteristics of headache before, during, and after COVID 19 infection, abortive headache medication, behavior, sleep disorders, anxiety, and depression.

**Results**. The study included 131 participants: 14 men (10.6%) and 117 women (89.31%), mean age - 37 ± 8,16 years. Before COVID 19 infection participants used analgesic drugs on 3.67 ± 2.96 days/month, during the COVID 19 infection month - 10.44 ± 8.81 days/month, and in the post-Covid period - 12.27 ± 9.73 days/month. From the study group, 9.1% of patients had medication overuse before COVID 19, during the Covid 19 period – 43%, and after the Covid 19 – 33%.

**Conclusion**: The study proved an increased analgesic consumption during and after the COVID 19 infection, possibly due to the association of a secondary headache namely headache attributed to infection. The other factors will be elucidated in further research.

## P0421 COVID-19 and Headache: Impact on Pre-existing and Characteristics of de novo: A cross-sectional Study

### J. Al-Hashel^1,2^, F. Abokalawa^2^, R. Alroughani^3^, M. Alenezi^4^, S. Farouk Ahmed^2,5^

#### ^1^Kuwait University, Medicine, Kuwait, Kuwait; ^2^Ibn Sina Hospital, MOH, Neurology, Kuwait, Kuwait; ^3^Amiri Hospital, MOH, Neurology, Kuwait, Kuwait; ^4^Farwanya hospita, MOH, Medicine, Kuwait, Kuwait; ^5^Faculty of Medicine, Minia University, Neuropsychiatry, Minia, Egypt

##### **Correspondence:** J. Al-Hashel; S. Farouk Ahmed

Background: Headache is a common symptom during and after acute respiratory syndrome coronavirus 2 (SARS-COV-2). Objective: To study headache character in relation to SARS-COV-2 infection. Methods: This was a cross-sectional study include patients who had SARS-COV-2 infection presented to headache clinic within 3 months after the onset of infections. Patients were diagnosed as primary headache disorders according to Headache characters were reported before and after SARS-COV-2 infections. Results: A total of 121 patients who recovered from SARS-COV-2 infections were included. Their mean age was 35.29 and of most of them were females 83.5%. Prior to SARS-COV-2 infections 70.2% had migraine and 14.9% experienced a tension-type headache while14.9% reported de novo headache post SARS-COV-2. The patient had significant increase in mean headache days 11.09 compared with 8.66 headache days before SARS-COV-2 infection (p < 0.006). Mean days of analgesic use (8.93versus 5.628; P

## P0422 Post Covid-19 rhino-orbital-cerebral mucormycosis presenting as unilateral headache

### A. Dubey^1^, S. Dubey^2^

#### ^1^GMC & Hamidia Hospital Bhopal, Bhopal, India; ^2^MMCH & RI, Department of Neurology, Kanchipuram, India

##### **Correspondence:** A. Dubey

Objective

Covid-19 pandemic has had a major impact on the health of people globally. Mucormycosis has come up as an important challenging epidemic in India in patients recovered from Covid-19 and with immunocompromised states like diabetes mellitus. We describe a case of rhino-orbital-cerebral mucormycosis here.

Methods

A 47 year old diabetic male was admitted in Covid unit for pulmonary symptoms. He received steroids along with the standard treatment and was discharged in a stable state. 15 days after discharge, he developed left sided dull aching headache of moderate intensity and increasing in the next 3 days and being unresponsive to analgesics.

Results

Examination showed tenderness over left cheek, reduced touch sensations over left upper face with mild ptosis in left eye.Lateral movements were restricted from left eye. These findings suggested left 3rd, 5th and 6th cranial nerve involvement. MRI brain and paranasal sinuses showed left cavernous sinus thrombosis with internal carotid artery thrombosis and left maxillary and ethmoid sinusitis. Considering the ongoing epidemic of mucormycosis in covid rcovered patients, endoscopic sinus biopsy was taken which showed aseptate hyphae consistent with mucormycosis.

Conclusion

Mucormycosis is an important post Covid complication especially in diabetic patients treated with steroids. Careful history and examination are needed for any such patient presenting with isolated headache.

## P0423 Can a Calcitonin Gene-Related Peptide (CGRP) receptor antagonist mitigate neuroinflammatory, hyper-immune, and nausea-like responses to SARS-CoV-2 infection in preclinical mouse models?

### S. Rahman^1^, D. Buchholz^2^, B. Imbiakha^2^, H. Aguilar-Carreno^2^, A. Luebke^1^

#### ^1^University of Rochester, Biomedical Engineering and Neuroscience, Rochester, MN, United States; ^2^Cornell University, Ithaca, United States

##### **Correspondence:** S. Rahman

In December 2019, the coronavirus disease (COVID-19) caused by SARS CoV-2 was identified. COVID-19 causes a respiratory illness like the flu with symptoms such as fever, cough, headache, chills, and nausea. The FDA has approved BiohavenPharmaceuticals to proceed to a clinical trial of its CGRP-receptor antagonist to treat patients with severe COVID-19, suggesting that the neuroinflammatory reaction that is initiated by CGRP in response to SARS-CoV-2 could be a therapeutic target for treating severe COVID-19. We were interested in testing if a CGRP receptor antagonist (olcegepant) would mitigate COVID-19 symptoms in mice. As a readout of SARS-CoV-2 infection symptoms, we have assessed weight loss, O_2_ saturation, temperature in young and old mouse models with CGRP receptor antagonized by olcegepant (2 mg/kg/day/SQ). In ongoing experiments, we will be also monitoring the presence of a nausea-like state by assessing hypothermic responses to provocative motion. To date, we have determined that CGRP receptor antagonism is only protective in older C57B6 mice, as there was no significant difference between CGRP receptor antagonism and placebo controls in younger mice. Ongoing studies will determine if CGRP antagonism is similarly protective against nausea - like symptoms. Information gained from these studies will provide a direct assessment of whether a CGRP-receptor antagonist can mitigate both mild and severe symptoms associated with SARS-CoV-2 infection.

## P0424 COVID-19 Associated Mucormycosis (CAM) with headache: An emerging case entity

### S. Dubey^1^, V. Ramakrishnan^1^, A. Dubey^2^, C. Mutharasu^1^

#### ^1^MMCH & RI, Department of Neurology, Kanchipuram, India; ^2^Gandhi Medical College, Department of Medicine, Bhopal, India

##### **Correspondence:** S. Dubey

Background and objective:

The coronavirus disease 2019 (COVID-19) pandemic has led to a large number of morbidity as well as mortality across the world. Out of its many complications, COVID-19 Associated Mucormycosis (CAM) has also emerged to be an important one. We here present an interesting case of CAM with headache.

Methods:

A 77 year old diabetic man presented with history of cough, breathlessness and fever. He was diagnosed to be COVID positive. HRCT chest showed 85-90% lung involvement with CT severity score 23/25. He was treated with oxygen, remdesivir and corticosteroids. During his stay, he started complaining of unilateral (right sided) headache.

Results:

There was nasal stuffiness with blackish discharge from nostrils along with right sided facial tenderness. CT head revealed heterogenous soft tissue lesion showing few hyperdense foci within the right maxillary sinus with associated thinning and rarefaction of medial wall of maxillary sinus and bones of ethmoidal sinus and sclerosis of lateral wall of maxillary sinus, suggestive of fungal sinusitis. Biopsy confirmed mucormycosis. He underwent surgical debridement and received amphotericin B and is currently on improving course.

Conclusions:

Any new headache during COVID-19 infection should be dealt with immediately. Mucormycosis cases associated with and after COVID infection have been on a rise lately probably because of immunocompromised states including diabetes and corticosteroid use.

**Fig. 1 (abstract P0424).**
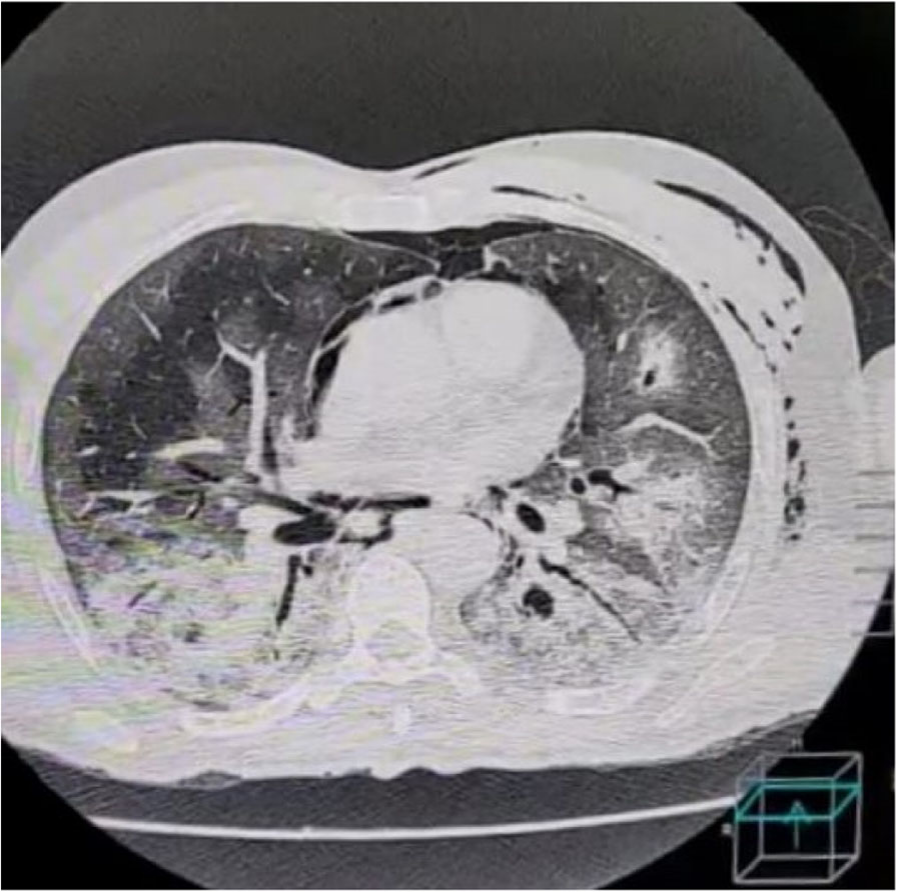
See text for description

**Fig. 2 (abstract P0424).**
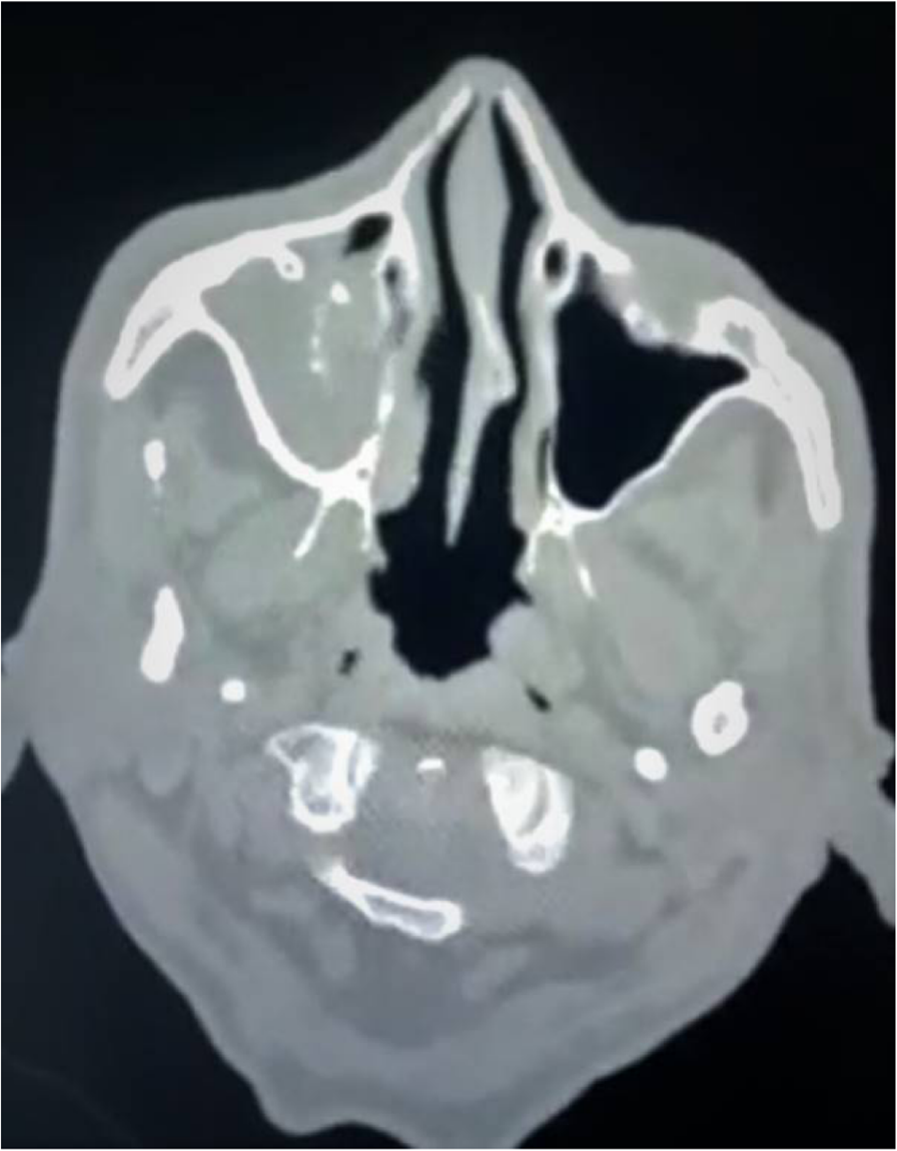
See text for description

## P0425 Efficacy of microvascular decompression in patients with trigeminal neuralgia

### A. Gusev, M. Kurnukhina, V. Cherebillo

#### First Pavlov State Medical University of St. Petersburg, Neurosurgical, St. Petersburg, Russian Federation

##### **Correspondence:** A. Gusev

**Summary**.Trigeminal neuralgia is one of the most persistent pain syndromes in clinical neurology. If there is no effect on the background of drug therapy,various surgical methods of treatment are used.One of the possible methods of surgical treatment is to perform microvascular decompression,which ensures a more stable regression of the pain syndrome.

**Materials and methods.** A clinical study of 40 patients aged 21 to 78 years (median 52,5 years) was conducted.The study of patients was carried out at three stages:before the operation,in the early and late postoperative periods.Retrosigmoid access was used.To assess the effectiveness of surgical treatment, the McGill pain intensity questionnaire, the SF-36 were used.

**Results and discussion**.The patients showed positive dynamics in the late postoperative period in the form of a significant decrease in the sensory,affective and evolutional scales of the McGill pain intensity questionnaire (p˂0,05).After surgery positive dynamics were revealed on all scales of the SF-36 questionnaire for all 40 patients:an increase in physical,role, social,emotional functioning,a significant decrease in the intensity of pain syndrome(p˂0,05).Only in 6,8% of cases, we noted a regression of the pain syndrome in the first 3 months after surgical treatment.

**Conclusion**. Microvascular decompression leads to an improvement in the quality of life in the late postoperative period and is an effective treatment method for patients with trigeminal neuralgia.

## P0426 Ocular pain due to orbital myositis

### I. Velichko, M. Barabanova, G. Muzlaev

#### Kuban State Medical University, Neurology and Neurosurgery, Krasnodar, Russian Federation

##### **Correspondence:** I. Velichko

**Objective**: Ocular myositis is a rare inflammatory disorder of single or multiple extraocular eye muscles. We report a woman presenting with ocular pain caused by inflammation of extraocular muscle.

**Methods**: Case report.

**Results**: A 40-year-old woman presented to the department of neurology because of onset of diplopia and ocular pain on the left. The onset of the disease occurred about 2 months ago with a headache in the left temporal region. Neurological examination revealed limited and painful adduction of the left eye. Pupillary light reflexes and dilated fundus examination were normal. Complete blood count, biochemical profile, erythrocyte sedimentation rate, and thyroid function tests were within normal limits. Antinuclear antibodies were negative. Magnetic resonance imaging (MRI) showed left medial rectus muscle enlargement. The endocrinologist and rheumatologist ruled out the pathology. Based on clinical characteristics, MRI results, the ophthalmologist diagnosed orbital myositis. Intravenous therapy with prednisolone was carried out, followed by oral administration of the drug at a dose of 60 mg per day with a gradual dose reduction. The treatment resulted in complete remission and pain relief within a few weeks.

**Conclusion**: Diagnosis, assessment and management of facial pain requires the cooperation of neurologists and ophthalmologists in order to achieve the best patient outcomes.

**Disclosure of Interest**

The authors declare no conflicts of interest.

## P0427 Intravenous lacosamide and phenytoin as treatment of acute pain exacerbations in trigeminal neuralgia: a retrospective analysis

### A. Muñoz-Vendrell^1^, S. Teixidor-Panella^1^, J. Sala-Padró^2^, S. Campoy^1,3^, M. Huerta^1,3^

#### ^1^Hospital de Bellvitge - IDIBELL, Headache Unit. Neurology Department., L’Hospitalet de Llobregat, Spain; ^2^Hospital de Bellvitge - IDIBELL, Epilepsy Unit. Neurology Department., L’Hospitalet de Llobregat, Spain; ^3^Hospital de Viladecans, Neurology Department., Viladecans, Spain

##### **Correspondence:** A. Muñoz-Vendrell

**Objective**: The aim of this descriptive study is to evaluate the efficacy and security of intravenous lacosamide (LCM) and phenytoin (PHT) in the treatment of acute pain caused by trigeminal neuralgia (TN).

**Methods**: We reviewed clinical records of patients who attended the emergency department at a tertiary hospital in Barcelona between 2012 and 2020 due to acute pain exacerbations related to TN and were treated for the first time with either intravenous PHT or LCM. We analyzed demographic features, TN characteristics and registered follow-up for at least 6 months. Primary endpoints were pain relief (defined as report by the patient, absence of further rescue medication and hospital discharge under 10 hours since treatment) and adverse effects during the hospital stay. We compared these variables between groups using Fisher"s exact test.

**Results**: We recorded 117 episodes from 95 patients (median age 59"2 years, 65"3% women). TN etiology was secondary in 10"5%. Out of 59 LCM infusions, pain relief was observed in 76"3% of cases with a 1"7% of adverse effects. Out of 58 PHT infusions, pain relief was observed in 75"9% with a 13"8% of adverse effects, all of them mild. There was no difference in pain relief between groups, but the percentage of adverse effects was significantly different (p=0.017).

**Conclusion**: Intravenous LCM and PHT can be effective and safe treatments for acute pain in trigeminal neuralgia. According to our series though, LCM might be better tolerated than PHT.

## P0428 Outcomes of treatment and psychometric performance of patient reported outcomes in Trigeminal Neuralgia - two Systematic Reviews

### C. Venda Nova^1^, J. M. Zakrzewska^1^, S. R. Baker^2^, R. Ni Riordain^1,3^

#### ^1^University College London, Eastman Dental Institute, London, United Kingdom; ^2^Sheffield University, School of Clinical Dentistry, Sheffield, United Kingdom; ^3^Cork University Dental School and Hospital, Oral Medicine, Cork, Ireland

##### **Correspondence:** C. Venda Nova

There are multiple treatment options in Trigeminal Neuralgia (TN) however, consensus is lacking as to what the important outcomes of treatment should be.Additionally, there is no clear choice for the correct measurement instrument to illustrate the impact of treatment.The objectives of these systematic reviews (SRs) were to summarize all the outcomes, patient reported outcomes (PROs) and their psychometric properties published in the literature to date.Two SRs were completed by searching multiple databases for a)all the TN studies with a surgical and/or a medical intervention to summarise the outcomes used to date mapped to the Initiative on Methods,Measurement, and Pain Assessment in Clinical Trials guidelines (IMMPACT);b)all the TN studies assessing psychometric properties of instruments based on Consensus-based Standards for the selection of Health Measurement Instruments guidance(COSMIN).In the intervention studies (n=467), most collected data on the impact of treatment on pain (n=459) and on side effects (n=386).A small number of studies collected data on the impact of treatment on physical (n=46) and emotional functioning (n=17).Of the 6 studies assessing psychometric properties of 5 PROs, the Penn Facial Pain Scale-Revised (Penn-FPS-R)was the only demonstrating promising content validity results.The lack of clearly defined outcomes and the poor psychometric performance of PROs creates difficulties in comparing studies and prevents the standardized reporting of results.

## P0429 Efficacy of surgical treatment in patients with trigeminal neuralgia secondary to multiple sclerosis – a prospective study of 18 cases with independent evaluation of outcome and complications

### N. Noory^1^, E. A. Smilkov^2^, T. B. Heinskou^1^, A. S. S. Andersen^1^, J. B. Springborg^3^, P. Rochat^3^, J. L. Frederiksen^4^, L. Bendtsen^1^, S. Maarbjerg^1^

#### ^1^Danish Headache Center, Department of Neurology, Glostrup, Denmark; ^2^Rigshospitalet-Glostrup, Department of Radiology, Glostrup, Denmark; ^3^Rigshospitalet-Glostrup, Department of Neurosurgery, Copenhagen, Denmark; ^4^Rigshospitalet-Glostrup, Department of Neurology, Glostrup, Denmark

##### **Correspondence:** S. Maarbjerg

**Background and Objective**

Trigeminal neuralgia (TN) is relatively common among patients with multiple sclerosis (MS). There is a lack of high-quality scientific evidence regarding efficacy of surgery in patients with TN secondary to MS (TN-MS). Such studies are crucial to counterbalance the potential gain from surgery and the risk of surgical complications.

**Methods**

Surgically treated patients with TN-MS were included from 2012 to 2019. The procedures were microvascular decompression, glycerol rhizolysis and balloon compression. Preoperatively, all patients underwent 3.0 Tesla MRI, a clinical examination including a semi structured interview. All patients were followed for 12 months and surgical complications were classified according to a predefined protocol.

**Results**

We included 18 TN-MS patients. Seven patients underwent microvascular decompression. Five (71%) had an excellent or good outcome. Three (43%) patients suffered major complications. Eleven patients underwent balloon compression and glycerol rhizolysis. Six (54%) patients had excellent or good outcome. Two patients (18%) had major complications.

**Conclusions**

Microvascular decompression was efficient in the majority of TN-MS patients, but the rate of major complications was high. Percutaneous procedures were effective in half of the operated patients. We recommend the use of percutaneous procedures in medically refractory TN-MS patients and microvascular decompression in well-selected patients.

## P0430 A joint medical and surgical multidisciplinary trigeminal neuralgia service eleven-year evaluation

### S. Singhota^1,2^, N. Tchantchaleishvili^3,4^, J. Wu^5^, L. Zrinzo^3^, L. Thorne^3^, J. M. Zakrzewska^1,6^

#### ^1^Royal National ENT & Eastman Dental Hospitals, London, United Kingdom; ^2^Medical School, University of Birmingham, Birmingham, United Kingdom; ^3^The National Hospital for Neurology and Neurosurgery, London, United Kingdom; ^4^University of Bordeaux, Bordeaux Neurocampus, Bordeaux, France; ^5^School of Dentistry, University of Leeds, Leeds, Germany; ^6^Pain Management Center, London, United Kingdom

##### **Correspondence:** N. Tchantchaleishvili

**Background**

Trigeminal Neuralgia (TN) is an episodic severe neuralgic pain resulting in significant impact on quality of life. It can be managed both medically and surgically.

**Aim**

To review all patients who attended a joint neurosurgeon and physician Multi-Disciplinary Team (MDT) clinic over a 11-year period and determine what treatments they underwent and their outcomes.

**Methods**

Using electronic health records, data was transferred to an excel spreadsheet to analyse a) patient demographics, referrer details, duration of TN, and drugs used at the time of referral b) pain status and drugs used: prior to the MDT, at the time of MDT, and at the last visit to the service c) the type and total number of surgical procedures. Surgical complications were classified according to the Ibanez model.

**Results**

337 patients attending the MDT between 2008-2019 were analysed of which 49 had previous surgery and were analysed separately. Of the remaining patients, 53% opted to have surgery following the MDT. At the last reported visit 55% of patients who opted to have surgery were pain free and off drugs, compared to 15.3% of medically managed patients. Surgical complications were mostly attributable to numbness and was temporary.

**Conclusion**

The MDT clinic offers an opportunity for shared decision making with patients deciding on their personal care pathway. In this cohort more than half of patients opted for surgery, and subsequently had better pain control and less drug therapy.

**Fig. 1 (abstract P0430).**
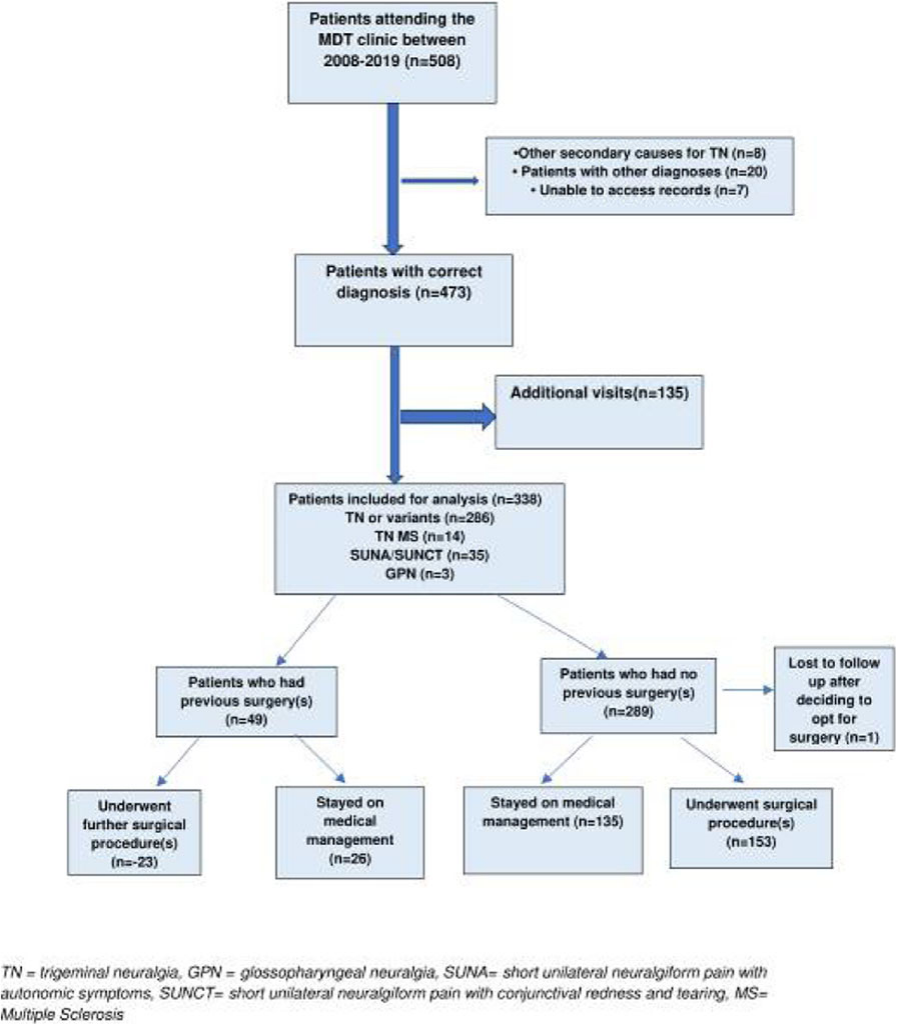
See text for description

## P0431 Episodic and chronic migraine differ in 1911a>g polymorphism of *trpv1* gene: a possible biomarker of migraine chronification?

### A. Yakubova^1^, R. Giniatullin^2^

#### ^1^Kazan Federal University, Kazan, Russian Federation; ^2^A.I. Virtanen Institute for Molecular Sciences, University of Eastern Finland, Kuopio, Finland

##### **Correspondence:** A. Yakubova

TRPV1 receptors expressed in trigeminal neurons are implicated in migraine pain. Recent genetic studies suggested that the single nucleotide polymorphism (SNP) 1911A>G affects functional activity of the receptors and is involved in different pain conditions. However, this SNP has not been tested in migraine.

Here was evaluated the frequency distribution of AA, AG and GG variants of 1911A>G in the *TRPV1* gene in healthy individuals and patients with episodic (EM) and chronic migraine (CM) to test the influence of the SNP on susceptibility to these forms of migraine.

The study included 106 patients with migraine (32 EM and 24 CM) and 50 healthy controls. DNA from peripheral blood was used to test *TRPV1* SNP using allele-specific PCR.

The genotype frequency distribution in EM was comparable with that in controls (AA-38%, AG-53%, GG-9% and AA-34%, AG-46%, GG-20%, respectively, *p*=0.467) but a tendency of GG variant frequency reduction is noticeable. In CM the distribution differed significantly from control and EM (*p*=0.012 and *p*=0.049): the AA genotype doubly increased, whereas the GG variant was completely absent, AA-67%, AG-33%, GG-0% (Fig.1).

This is first indications of distinctive involvement of *TRPV1* 1911A>G genotypes in EM and CM. Our data reveal a different predisposition to chronic pain in migraine and give a new look at the nature of its chronification, proposing that the absence of GG genotype may be considered as potential biomarker of migraine chronification risk

**Fig. 1 (abstract P0431).**
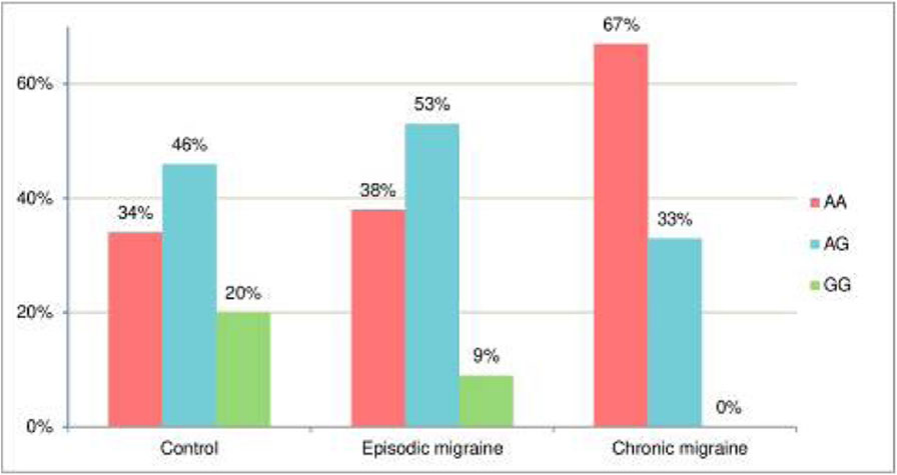
See text for description

## P0432 The Index Vein is a highly specific sign for migraine with aura in the emergency setting

### A. Scutelnic^1^, V. Petroulia^2^, L. Schraml^3^, S. Jung^4^, M. Branca^5^, M. Beyeler^4^, U. Fischer^4^, R. Wiest^2^, N. Slavova^2^, C. Schankin^1^

#### ^1^Inselspital Bern, Neurology, Bern, Switzerland; ^2^Inselspital Bern, Neuroradiology, Bern, Switzerland; ^3^Inselspital Bern, Internal Medicine, Bern, Switzerland; ^4^Inselspital Bern, Neurology, Bern, Switzerland; ^5^University of Bern, Bern, Switzerland

##### **Correspondence:** A. Scutelnic

Objective: To assess the relevance of the "index vein" (IV) for making the correct diagnosis of migraine with aura (MwA) in patients with acute neurological deficits. We tested the hypothesis that the prevalence of the IV differs between migraine aura, epilepsy, ischemic stroke, and controls. Methods: The IV was defined according to our previous work (Slavova et al. Neurology;94(24):e2577-e2580): a single prominent vein in susceptibility weighted imaging draining the cortical area of the neurological deficit in the absence of diffusion abnormality and occlusion in angiography. For this retrospective study, 400 patients were included when they (i) presented at our emergency department with an acute neurological deficit, (ii) had a brain MRI within eight hours after the symptoms stopped, and (iii) had a discharge diagnosis of migraine aura, ischemic stroke, epilepsy or none of these (controls, n=100 per group). The primary hypothesis was that there is a difference distribution of IV in the four conditions (chi-square test). Results: Compared to stroke (n=2), epilepsy (n=5) and controls (n=1), the IV is more prevalent in migraine aura (n=17, chi-square 27.8, p<0.001). Although the sensitivity is low (17%), we found the IV to be highly specific for migraine aura (specificity 97%, 95% CI: 95-99%). The finding of IV has positive predictive value for the diagnosis of migraine aura of 70% (95%CI 48-87). Conclusions: When present, the IV could serve as a good biomarker for migraine aura in the emergency setting.

## P0433 A Mathematical Interpretation of the International Classification of Headache Disorders 3rd Edition

### P. Zhang

#### Rutgers Robert Wood Johnson Medical School, New Brunswick, NJ, United States

Background:

International Classification of Headache Disorders (ICHD3) provides definition of headache disorders that can be used to construct a mathematical basis for headache classification. We seek to construct a theoretical framework for such a construction.

Methods:

Headache and facial pain conditions are interpreted as bundles of phenotypes. ICHD3 diagnoses are then defined as sets. We proceed to show that observations, in the form of theorems, can be proved with our set theoretic construction for the ICHD3.

Results:

In order for our set theoretic construction to be mathematical consistent, the all-present "not accounted for by another ICHD3 diagnosis" criterion must be excluded. Furthermore, our system can be used to construct a categorical approach to headache medicine in the tradition of category theory.

Conclusions:

Mathematical interpretation of ICHD3 is possible and may provide significant implication for understanding the structure and organization of headache diagnostic classification.

## P0434 Artificial intelligence: a useful tool in headache consultations

### M. A. Ruiz Yanzi^1^, M. V. Nagel^1^, S. Crema^1^, M. Grandinetti^1^, D. Calvo^1^, M. Olivier^1^, N. Larripa^1^, M. T. Gutiérrez^1^, S. Cavanagh^1^, L. Bonamico^1^, F. Dorr^2^, D. Fernández Slezak^2,3^, M. Farez^4^, M. T. Goicochea^1^

#### ^1^Fleni, Buenos Aires, Argentina; ^2^Entelai, Buenos Aires, Argentina; ^3^UBA, Facultad de Ciencias Exactas, Buenos Aires, Argentina; ^4^CEBES, Buenos Aires, Argentina

##### **Correspondence:** M. V. Nagel

Objective:to implement an artificial intelligence (AI) based software as a tool for standardizing headache consultations.

Methods: Prospective study in collaboration with Novartis. Between May and November 2019, in the Headache Section of our institution, an AI software (ENTELAI PRE-DOC) was implemented. Patients completed an electronic survey in the waiting room registering sex, age, MIDAS (Migraine Dissability Assessment Scale), HIT-6 (Headache Impact Test), and clinical characteristics of headache.

The AI software estimated a diagnosis. This information was used by the headache specialist during consultation.

Results: 757 patients were included (84.3% women). Average age 41 years. Migraine was the main diagnosis (90.9%), and of those, 39% were chronic.

29.4% described hemicranial pain, only 29.8% pulsatile pain, 65.6% photophobia and phonophobia, 69.3% nausea/vomiting, and 59% worsening of pain with exercise. 66.9% of patients had MIDAS scores higher or equal to 11 (moderate to severe disability) and 73.1% had HIT-6 scores higher or equal to 56 (severe impact).

Conclusions: ENTELAI PRE-DOC facilitated recollection of standardized data for diagnosis and assessment of functionality in headache patients. In our population, headache was highly disabling and had an important impact in functionality.

This software could be useful when access to headache specialists is difficult, and to generate standardized data collection for a better analysis of headache.

## P0435 Early maladaptive schemas is maybe a good predictor of adolescent migraine

### G. Guler Aksu^1^, O. Kayar^2^, A. Özge^3^

#### ^1^Mersin University, Department of Child and Adolsecent Psychiatry, Mersin, Turkey; ^2^Çankırı Karatekin Univeristy, Department of Psychology, Çankırı, Turkey; ^3^Mersin University, Department of Neurology, Mersin, Turkey

##### **Correspondence:** G. Guler Aksu

Despite many findings from studies, there is still unknown points why migraine and psychiatric disorders are highly comorbid, what is the underlying pathophysiology in this comorbidity, how do we treat. Moreover, schemas which is the basic structure of cognition is understudied although cognitive behavioral therapy based studies are more. This study examined gender effect on early maladaptive schemas (EMS) and migraine in adolescent migraineurs. It comprised 171 adolescents (67.3% girls, n=115) aged 12-18 years. The migraine clinic characteristics, accompanying symptoms and EMS were evaluated by gender. Differences were tested controlling for psychopathology and abuse. Mean age was 15.37±1.87 in girls; 15.23±1.98 in boys (p=0.672). There was no difference the migraine clinic characteristics and except from dizziness all other accompanying symptoms were similar between genders. As schema domain, disconnection and rejection in females, impaired limits in males are more frequent. Also defectiveness/shame, emotional deprivation, abondanment/instability, dependence/incompetence, vulnerability harm or illness schemas were more frequently in females, insufficient self-control/ self-discipline schemas so were in males more. This concluded EMS was different in females and males in earlier times before the clinical presentation of migraine was not differentiated in adolescents. EMS may have an effect the clinical presentation of migraine and prognosis potentially chronification.

**Fig. 1 (abstract P0435).**
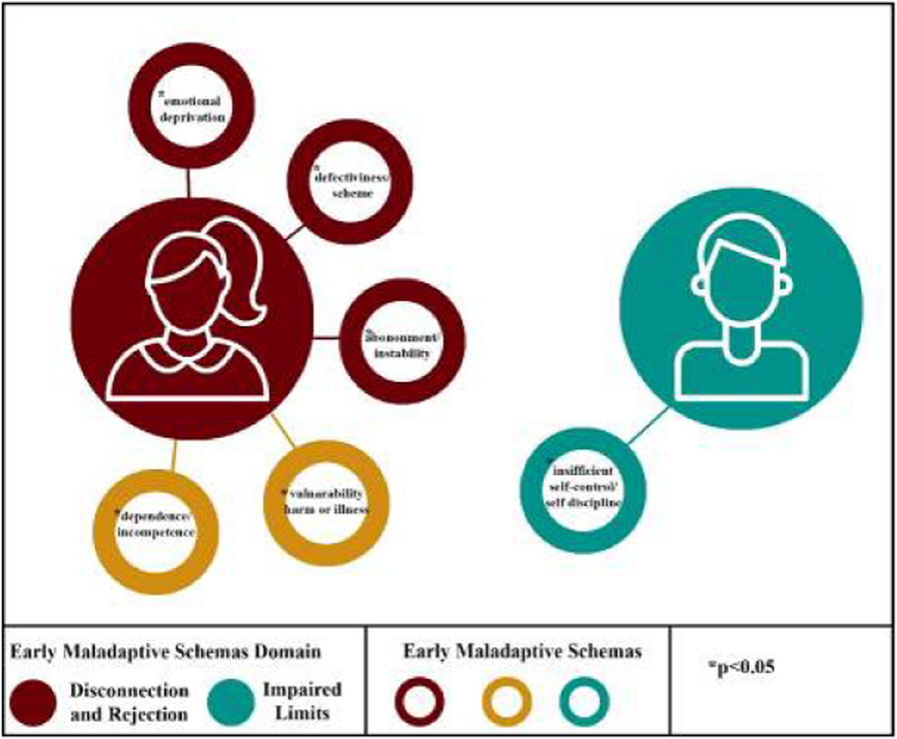
See text for description

## P0436 Headache and musculoskeletal pain in school children are associated with uncorrected vision problems and need for glasses: a case–control study

### H. M. S. Thorud, R. Aurjord, H. K. Falkenberg

#### University of South-Eastern Norway, Department of Optometry, Radiography and Lighting Design, Kongsberg, Norway

##### **Correspondence:** H. M. S. Thorud

Musculoskeletal pain and headache are leading causes of years lived with disability, and an escalating problem in school children. Children spend increasingly more time reading and using digital screens, and increased near tasks intensify the workload on the precise coordination of the visual and headstabilizing systems. Even minor vision problems can provoke headache and neck- and shoulder (pericranial) pain. This study investigated the association between headaches, pericranial tenderness, vision problems, and the need for glasses in children. An eye and physical examination was performed in twenty 10–15 year old children presenting to the school health nurse with headache and pericranial pain (pain group), and twenty age-and-gender matched classmates (control group). The results showed that twice as many children in the pain group had uncorrected vision and needed glasses. Most children were hyperopic, and glasses were recommended mainly for near work. Headache and pericranial tenderness were significantly correlated to reduced binocular vision, reduced distance vision, and the need for new glasses. That uncorrected vision problems are related to upper body musculoskeletal symptoms and headache, indicate that all children with these symptoms should have a full eye examination to promote health and academic performance. Thorud, H. S., Aurjord, R. & Falkenberg, H. K. Headache and musculoskeletal pain in school children are associated with uncorrected vision problems and need for glasses: a case-control study. *Sci Rep* **11**, 2093, doi:10.1038/s41598-021-81497-w (2021).

## P0437 Urine caffeine concentration vs. headache severity in pediatric patients

### T. Hikita^1^, H. Goda^2^, Y. Ogawa^2^, T. Kudo^2^, K. Ito^2^

#### ^1^Hikita Pediatric Clinic, Kiryu City, Japan; ^2^Research Institute of Pharmaceutical Sciences, Musashino University, Tokyo, Japan

##### **Correspondence:** T. Hikita

**Background**: Caffeine is sometimes used for headache treatment. On the other hand, caffeine consumption is a risk factor for chronic migraine.

**Aim**: We evaluated correlations among urine caffeine concentration, sleep time, and headache severity in patients.

**Methods**: Study subjects were 41 patients who came to Hikita Pediatric Clinic for headache treatment. Informed consent was obtained, and study design was approved by Musashino University Ethics Committee. Urine caffeine concentrations were determined by LC-MS/MS, and data were collected for age, sleep time, HIT-6, PedMIDAS, and headache severity score (range 0-10).

**Results**: Subjects' ages ranged from 5 to 19 yrs (median 13). Diagnoses and #s of cases were: Migraine without aura (MWO) 22, Migraine with aura (MWA) 4, Orthostatic disease (OD) 3, MWO+OD 3, Probable MWO 3, Other 6. Significant correlations were observed for HIT-6 and PedMIDAS scores (ρ: 0.56), and for HIT-6 and headache severity scores (ρ: 0.65), but not for other combinations of factors. There were 8 urine caffeine-negative (level <0.0625 μM) cases, and 33 urine caffeine-positive cases. For these two groups, respective median values for parameters were: age 12.5 vs. 13 yrs; weekday sleep time 9 vs. 7.75 hrs; weekend sleep time 9.75 vs. 9 hrs; HIT-6 score 61.5 vs. 64, PedMIDAS score 13 vs. 20, headache severity score 4 vs. 5.

**Conclusion**: Headache severity was greater for urine caffeine-positive than for urine caffeine-negative cases.

## P0438 Headache in pediatric patients with muscular dystrophies: An observational study

### Y. Levinsky, T. Eidlitz-Markus

#### Schneider Children’s Medical Center, Givaat Shmuel, Israel

##### **Correspondence:** Y. Levinsky

**Background and objective**: Studies have shown that Duchenne and Becker muscular dystrophies are associated with chronic pain, but headache in particular has not been investigated. The aim of the study was to evaluate the prevalence and features of headache in Duchenne and Becker muscular dystrophies.

Methods: The cohort included 68 male patients aged 3-18 years (mean 3.8±9.0 years with Duchenne (n=49) or Becker (n=20) muscular dystrophy attending a tertiary neuromuscular clinic in 2015-2019. The parents and older patients completed demographic and headache questionnaires followed by the Strengths and Difficulties Questionnaire (SDQ). Patients reporting headache were referred for further evaluation.

Results: Twenty-two of the 68 patients (32.4%) reported headaches: 10 (45.5%) were subsequently diagnosed with migraine and 12 with tension-type headache. Patients with headache were older than patients without headache (p <0.001) and had a higher rate of pain in other organs (p<0.01), higher score on the emotional problems scale of the SDQ (p=0.001), and higher rate of parental headache (p <0.001).

**Conclusions**: Headache occurs in about one-third of patients with muscular dystrophy and is associated with older age, more emotional problems in SDQ scale , higher rate of pain in other organs, and higher rate of parental headache

**Fig. 1 (abstract P0438).**
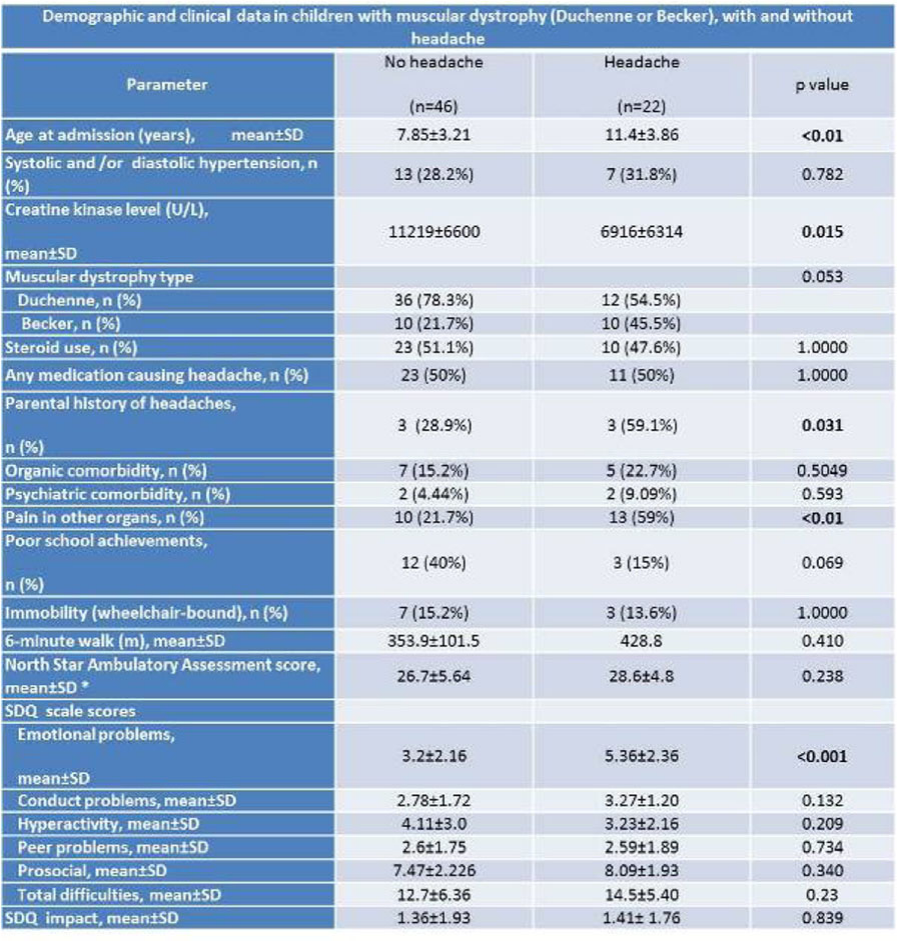
See text for description

## P0439 Risk of cochlear disorders in patients with in migraine and non-migraine headache

### S. Jong-Hee^1^, L. Sang-Hwa^1^, C. Byoungchul^1^, K. Jong-Ho^2^, K. Young-Suk^2^

#### ^1^Chuncheon Sacred Heart Hospital, Department of Neurology, Chuncheon, South Korea; ^2^Chuncheon Sacred Heart Hospital, Department of Anesthesiology and Pain Medicine, Chuncheon, South Korea

##### **Correspondence:** S. Jong-Hee

**Background:** Headache, especially migraine, has been associated with various vestibular symptoms and syndromes. Also, tinnitus and hearing impairment are prevalent among migraine patients. But, it is not yet clear whether headache including migraines are related to cochlear disorders. Thus, we sought to investigate possible associations between headache and cochlear disorders. **Methods:** We analyzed clinical data from the smart CDW from 2011 to 2021. In patients with migraine and non-migraine headache, MD, BPPV, vestibular neuronitis (VN) and cochlear disorders, such as hearing loss and tinnitus, were collected and compared to clinical data from controls who had health check-ups without headache. **Results:** Participants included 15,128 with migraines, 76,773 patients with non-migraine headache and controls were identified based on propensity score matching (PSM). After PSM, the odds ratios (ORs) in subjects with migraine versus controls were 2.59 for MD, 2.05 for BPPV, 2.98 for VN, 1.74 for hearing loss, and 1.97 for tinnitus, respectively (*p*<0.001). The OR for MD (1.77), BPPV (1.73), VN (2.05), hearing loss (1.40), and tinnitus (1.70) in patients with non-migraine was also high after matching (*p*<0.001). **Conclusions:** Our findings suggest that migraine and non-migraine headache are associated with an increased risk of cochlear disorders, in addition to vestibular disorders.

## P0440 Cost estimation of productivity loss, healthcare resource utilization and symptom burden associated with migraine in Indian healthcare setting

### J. R. Chaudhuri^1^, T. K. Banerjee^2^, R. Kulkarni^3^, S. Singh^4^, S. Gokhale^5^, S. Anand^5^, S. Thakur^6^, A. Thorat^6^

#### ^1^Yashoda Hospitals, Neurology, Hyderabad, India; ^2^National Neurosciences Centre, Neurology, Kolkata, India; ^3^Deenanath Mangeshkar Hospital and Research Centre, Neurology, Pune, India; ^4^Agrim Institute of Neurosciences, Artemis Hospitals, Neurology, Gurugram, India; ^5^Novartis Healthcare Private Limited, Market Access, Mumbai, India; ^6^Novartis Healthcare Private Limited, Medical Affairs, Mumbai, India

##### **Correspondence:** S. Gokhale

Objective: Migraine prevalence peaks in Indian patients during their most productive years leading to an economic burden. There are no scientific tools or literature to uncover this burden and is often ignored. We developed an evidence-based tool estimating migraine related costs associated with productivity loss, resource utilization and symptom burden

Methods: Work productivity and activity impairment data specific to India (N=263) was adopted from MyMigraineVoice online survey conducted from Sept-2017 to Feb-2018 in patients having at least four monthly migraine days and a failure on prophylactic therapy. Literature review was conducted to get insights about local epidemiology and costs. Tool enables user to input patient numbers, preventive treatment failure (no preventive treatment, no failure, 1 failure and 2+ failures), time horizon (1-12 months) and earnings per day to present cost estimates in Indian rupees (₹)

Results: Tool dynamically demonstrates total cost of migraine based on user provided inputs. Productivity loss was attributed to absenteeism and presenteeism. Resource utilization included costs associated with overnight hospitalizations and emergency visits. Expenses related to brain scans and pharmacological management were considered as symptom burden

Conclusion: In a self-pay market like India, this tool will enable decision makers to make an evidence-based decision. Increasing cost burden in migraine can be reduced by optimal preventive treatment

**Fig. 1 (abstract P0440).**
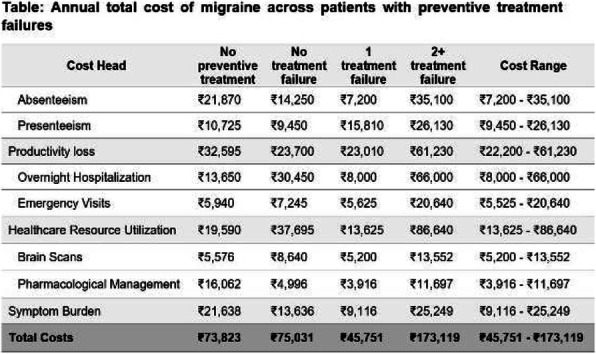
See text for description

**Fig. 2 (abstract P0440).**
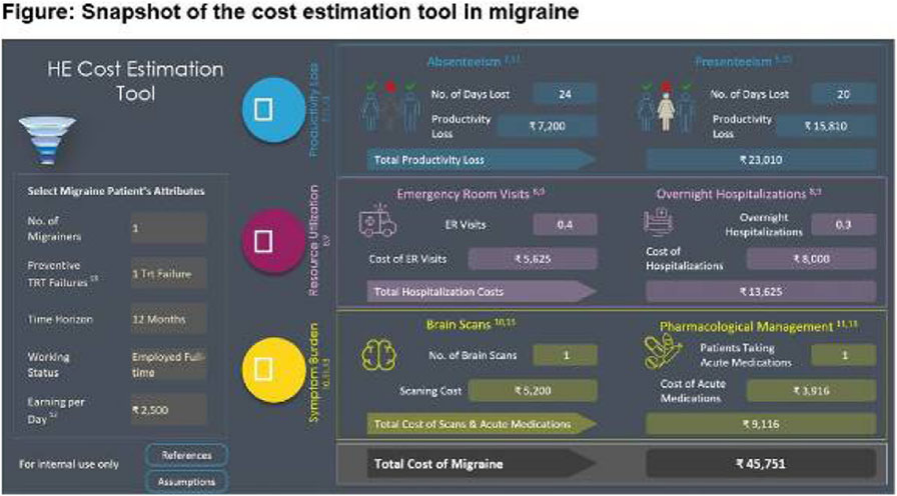
See text for description

## P0441 Migraine Diagnosis, Disability, and Work Productivity Impact in Migraine: Results of the OVERCOME (International) Study

### R. B. Lipton^1,2^, A. M. Nelson^3^, R. A. Nicholson^3^, A. Zagar^3^, Y. Kim^3^, J. Pascual^4^, S. Evers^5^, K. Hirata^6^, E. Pearlman^3^

#### ^1^Albert Einstein College of Medicine, Bronx, United States; ^2^Montefiore Medical Center, Bronx, United States; ^3^Eli Lilly and Company, Indianapolis, IN, United States; ^4^University Hospital Marqués de Valdecilla, University of Cantabria and IDIVAL, Santander, Spain; ^5^Krankenhaus Lindenbrunn, Coppenbrügge and University of Münster, Munster, Germany; ^6^Dokkyo Medical University, Mibu, Tochigi, Japan

##### **Correspondence:** R. B. Lipton

**Objective:** Quantify migraine diagnosis rates and burden among respondents to an ObserVational survey of the Epidemiology, tReatment, and Care Of MigrainE (OVERCOME). **Methods:** Web-based OVERCOME country-specific surveys were fielded in 2020-2021 in US, Spain, Germany, and Japan. Respondents who met modified ICHD-3 criteria for migraine via a validated diagnostic screener were identified. Respondents self-reported if they had a medical diagnosis (SR-MD) of migraine. Migraine-related disability (MIDAS), interictal burden (MIBS-4), and work productivity and activity impairment due to migraine (WPAI-M) were assessed for each country and internationally (equally weighted by country). Descriptive statistics were conducted. **Results:** Among respondents (N=57,837), 52,382 (91%; country-specific range: 82-97%) met ICHD-3 criteria for migraine. Among those (mean age 40.3 years; 66% female), 52% (range: 47-54%) had SR-MD of migraine; 55% (range: 37-67%) reported at least mild disability (i.e., being disabled by migraine, on average >2 days/month); 42% (range: 30-48%) had severe interictal burden. Respondents reported on average, 47.0% (range: 37.7-52.8%) overall work impairment and 41.3% (range: 37.9-43.2%) activity impairment due to migraine. **Conclusion:** Across countries, just over half of OVERCOME respondents had SR-MD of migraine, although the majority were at least mildly disabled and had substantial overall work and activity impairment due to migraine. **Sponsor:** Eli Lilly and Company.

**Fig. 1 (abstract P0441).**
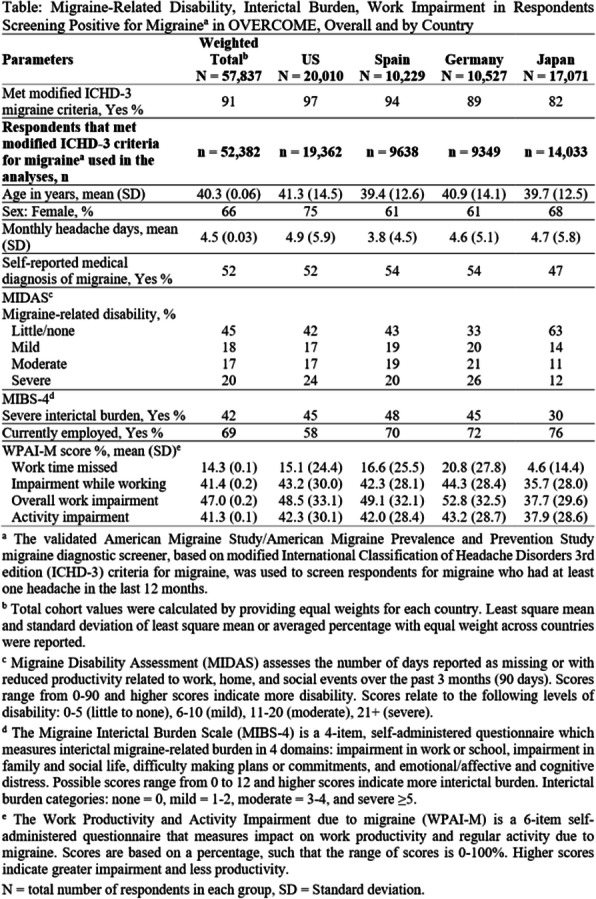
See text for description

## P0442 Pain intensity and cranial autonomic features in migraine: results from the American Registry for Migraine Research

### B. Benkli^1^, W. Zhang^2^, G. Dumkrieger^3^, M. J. Burish^4^

#### ^1^UT Health Neuroscience, Neurology, Houston, TX, United States; ^2^Department of Biostatistics and Data Science, UTHealth School of Public Health, Public Health, Houston, TX, United States; ^3^Mayo Clinic, Neurology, Scottsdale, AZ, United States; ^4^UT Health Neuroscience, Neurosurgery, Houston, TX, United States

##### **Correspondence:** B. Benkli

Objective: To determine the pain intensity of migraine and its relationship with cranial autonomic features.

Methods: The analysis included 1108 migraine patients who were enrolled in the American Registry for Migraine Research (ARMR) between February 2016 and June 2019. Patients completed the 0-10 numerical rating scale for pain intensity, as well as questions about associated features including all cranial autonomic features listed for cluster headache in the International Classification of Headache Disorders (ICHD) edition 3 beta. Patients also completed Generalized Anxiety Disorder-7 (GAD-7), Patient Health Questionnaire-4 (PHQ-4) and Migraine Disability Assessment Scale (MIDAS) questionnaires.

Results: Average pain intensity was 6.0 ±1.7, and 92.7% of patients in ARMR had moderate or severe pain. Higher pain intensity correlated significantly with higher GAD7, PHQ4, and MIDAS scores (p<0.0015). At least one cranial autonomic symptom was present in 83.8% of patients, and higher pain intensity correlated significantly with a higher rate of conjunctival injection, eyelid edema, and miosis (p<0.0005) along with increased restlessness (p=0.0001) and vomiting (p=0.0014).

Conclusions: Pain intensity in migraine correlates with headache burden (mood and disability scores) in a large clinical dataset, similar to population studies. A graded response of the trigeminal autonomic reflex appears to be a feature of not only cluster headache, but also migraine.

**Fig. 1 (abstract P0442).**
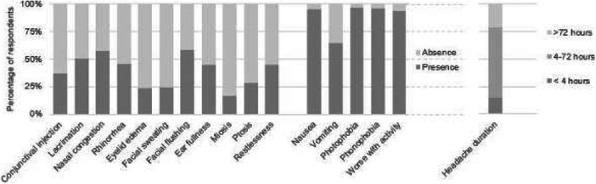
See text for description

**Fig. 2 (abstract P0442).**
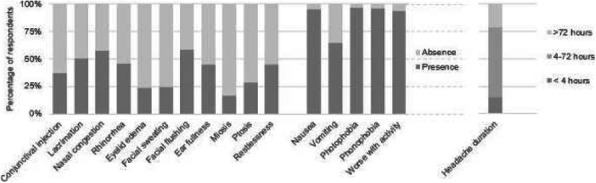
See text for description

## P0443 Clinical Characteristics, Treatment Satisfaction and Barriers to Treatment for Patients with Migraine: Results from OVERCOME (EU), the European Observational Survey of the Epidemiology, Treatment and Care of Migraine

### S. Evers^1^, T. Panni^2^, H. P. Hundemer^2^, D. Novick^2^, T. Treuer^2^, G. Dell Agnello^2^, J. Pascual^3^

#### ^1^University of Münster, Munster, Germany; ^2^Eli Lilly and Company, Indianapolis, IN, United States; ^3^Hospital Universitario Marqués de Valdecilla, Santander, Spain

##### **Correspondence:** S. Evers

**Objective:** Migraine is a chronic neurological disease with a considerable economic and societal burden. The objective of this study is to describe the demographics, quality of life, levels of treatment satisfaction and barriers to treatment for people with migraine.

**Methods:** Descriptive data is provided from the European ObserVational survey of the Epidemiology, tReatment and Care Of MigrainE; OVERCOME (EU), a large, cross-sectional, population level, web-based survey of adults (≥18 years) with migraine in Spain and Germany (Oct 2020 - Feb 2021).

**Results:** Overall, 20,756 respondents were evaluated, 57.6% had a migraine diagnosis, mean age of respondents was 40.4 years and 60.3% were female. With respect to Migraine Disability Assessment scores 39.4% of respondents reported little to no disability, 19.2% mild disability, 19.3% moderate disability and 22.2% severe disability. The top 3 barriers to taking preventative medication were i) efficacy of acute medication, ii) migraine not being serious enough for treatment and iii) concerns regarding side-effects. Of the patients taking preventative medications 21.1% found their medications to be a little efficacious, 37.7% somewhat, 22.9% a lot, and only 9.8% reported experiencing complete efficacy.

**Conclusion:** These results provide a unique insight into the levels of disability, satisfaction with currently available preventative treatments and existing barriers to treatment experienced by people with migraine in the EU.

## P0444 Demographics, Clinical Characteristics, Healthcare Provider Visits and Care Seeking Behavior Data from the European Observational Survey of the Epidemiology, Treatment and Care of Migraine: OVERCOME (EU)

### J. Pascual^1^, T. Panni^2^, H. P. Hundemer^2^, D. Novick^2^, T. Treuer^2^, G. Dell Agnello^2^, S. Evers^3^

#### ^1^Hospital Universitario Marqués de Valdecilla, Santander, Spain; ^2^Eli Lilly and Company, Indianapolis, IN, United States; ^3^University of Münster, Munster, Germany

##### **Correspondence:** T. Panni

**Objective:** Migraine is a chronic neurological disease with a considerable economic and societal burden. The objective of this study is to describe the demographics, clinical characteristics, and care seeking behaviors of people with migraine.

**Methods:** Descriptive data is provided from the European ObserVational survey of the Epidemiology, tReatment and Care Of MigrainE; OVERCOME (EU), a large, cross-sectional, population level, web-based survey of adults (≥18 years) with migraine in Spain and Germany (Oct 2020 - Feb 2021).

**Results:** In total 20,756 respondents were evaluated, 57.6% had a migraine diagnosis (mean age at diagnosis, 24.2 years), 60.3% were female and the overall mean age was 40.4 years. A total of 13,759 (66.3%) respondents reported 0-3 headache days per month (HD/month), 4,203 (20.2%) 4-7, 1,730 (8.3%) 8-14 and 1,064 (5.1%) 15+ HD/month. The most frequently reported comorbidities were allergies/hay fever (38.8%), anxiety (26.2%) and depression (24.0%). In the preceding 12 months 59.1% of participants visited a general practitioner, 39.4% a pharmacist, and 16.6% a general neurologist. The most severely affected individuals were more likely to have consulted a headache/pain specialist in the previous 12 months, 39.1% of total visits were made by individuals with Migraine Disability Assessment scores of 21+ (MIDAS IV).

**Conclusion:** This real-world study provides a unique insight into the demographics and care-seeking behaviors of people with migraine in the EU.

## P0445 Prevalence and attributed burden of headaches disorders in the general population of Benin

### T. Adoukonou^1^, M. Agbetou^1^, E. Dettin^1^, O. Kossi^1^, D. Houinato^1^, T. J. Steiner^2,3^

#### ^1^Department of Neurology, University of PARAKOU, BORGOU, Parakou, Benin; ^2^Lifting The Burden, 21-27 Lamb’s Conduit Street, United Kingdom; ^3^Department of Neuromedicine and Movement Science, Norwegian University of Science and Technology, Trondheim, Norway, Trondheim, Norway

##### **Correspondence:** T. Adoukonou

Headaches disorders globally are common and disabling. The aim of this study, within the Global Campaign against headache, was to determine their prevalence and attributabled burden in the general population of Benin. Disorders of interest, because of their public health importance, were migraine, tension-type headache (TTH) and disorders characterised by headache occuring on ≥ 15days/month (H15+) including probable medication-overuse headache (pMOH). In a cross-sectional study we randomly selected 2400 adults aged 18-65years from two regions of the country, one in the north and one in the south, taking rural (70%) and urban (30%) dwellers from each. In home visits, we interviewed each participant using HARDSHIP structured questionnaire applying ICHD-3 diagnostic criteria and several measures of burden. The study was approved by the ethics committee of biomedical research of University of Parakou. The mean age of participants was 32.1+/-11.2years. The overall 1-year prevalence of headaches was 76.5% (95%CI : 74.7-78.1). Adjusted for age and gender, the prevalence of migraine was 26.1%, of TTH 46.2% and H15+ 3.3% (including pMOH 2.2%). One-day prevalence (Headace yesterday) was 15.4%, implying that almost one in six adults had headache on any day. Attributed burden was measurable especially in lost productive time and impaired quality of life. The prevalence of headaches reported in Benin is similar to those reported in Ethiopia and Zambia and higher than averages.

## P0446 Vertebrobasilar alterations in migraine - driven only by a subgroup?

### O. Hensel^1^, P. Burow^1^, T. Kraya^2^, S. Nägel^1^

#### ^1^University of Halle, Neurology, Halle, Germany; ^2^St. Georg Hospital Leipzig, Neurology, Leipzig, Germany

##### **Correspondence:** O. Hensel

Objective: There is an ongoing debate regarding the relevance of arterial changes in migraine, as plenty but inconclusive evidence exists. We investigate whether alterations of the vertebrobasilar arteries occur in patients with migraine retrospectively analyzing MRI angiographies.

Methods: In 63 patients with episodic and chronic migraine (30.6±8.9 years, 84% women) and 126 age- and sex-matched controls, we determined the outlet angle of the superior cerebral artery (SUCA) in a coronal TOF-MRI (see Figure).

Results: Across all patients, the SUCA outlet angle was reduced in patients with migraine compared to controls (159±26° vs. 169±29°, p=0.020). However, this appears to be driven by a subset of the patients, as not all migraine patients have decreased SUCA outlet angles. Analyzing only the patients with reduced SUCA outlet angles (1st cut-off: median of patients 160°, 2nd cut-off: mean-standard deviation of controls 140°) shows that patients with chronic migraine have lower SUCA angles compared to patients wih episodic migraine (1st cut-off: 125±25° vs.142±12°, p=0.025; 2nd cut-off: mean-SD 110±20 vs. 131±6; p=0.004).

Conclusion: Migraine patients showed reduced SUCA outlet angle. This only appears to be relevant in a subset of patients, which could reflect different genetic constitution and correspond to a dilation of longitudinal vessel wall structures.

**Fig. 1 (abstract P0446).**
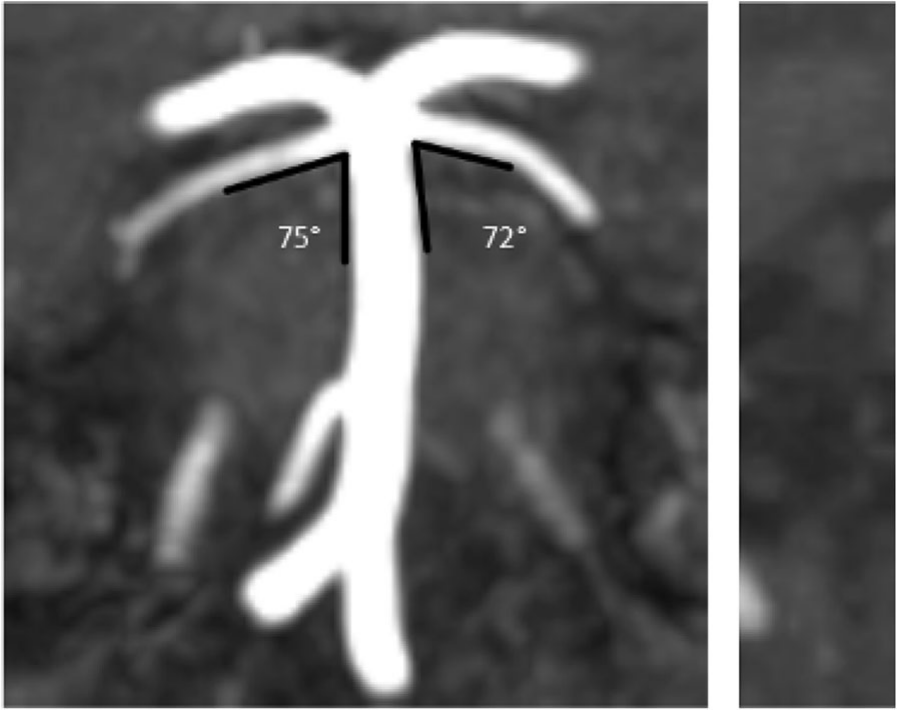
See text for description

## P0447 EEG-informed fMRI reveals rhythm specific activations during trigeminal pain processing

### H. Basedau, K. P. Peng, A. May, J. Mehnert

#### UKE, Institute for Systems Neuroscience, Hamburg, Germany

##### **Correspondence:** J. Mehnert

Objective: Non-invasive imaging studies of the trigemino-vascular system are limited by either spatial or temporal resolution. Multimodal imaging techniques - simultaneous functional magnetic resonance imaging (fMRI) and electroencephalography (EEG) – may solve this issue.

Methods: We conducted a - visual and trigeminal pain paradigm in 34 healthy volunteers in standalone EEG and fMRT sessions. Additionally, 17 volunteers participated in a simultaneous EEG-fMRI session. We then validated a novel non-parametric fusion technique, which exploits trial-to-trial variance.

Results: We reproduced previous findings and show correlations between both modalities. EEG power changes in theta-band induced by trigeminal pain correlate with fMRI activation within the brainstem, whereas those of gamma band correlate with cortical areas.

Conclusion: Our study validates a trigeminal nociceptive paradigm for EEG-fMRI fusion using standalone sessions and a visual paradigm as control. Power changes of the theta band induced by trigeminal nociception correlated with the nociceptive and anti-nociceptive pain processing systems in the brainstem while gamma band activity related to pain cortical networks of nociception and saliency. While our findings on the experimental side should be extended to headache patients, our analytical approach can be adapted to any multimodal analysis.

## P0448 Depicting areas of cerebral blood flow changes in nitroglycerin-induced cluster headache attacks using arterial spin labelling

### D. Y. Wei^1,2^, O. O’Daly^3^, F. O. Zelaya^3^, P. J. Goadsby^1,2^

#### ^1^King’s College, NIHR Wellcome Trust King’s Clinical Research Facility, London, United Kingdom; ^2^King’s College, Headache Group, Wolfson Centre for Age-Related Diseases, London, United Kingdom; ^3^King’s College, Centre for Neuroimaging Sciences, Department of Neuroimaging, London, United Kingdom

##### **Correspondence:** D. Y. Wei

Objectives: To investigate cerebral blood flow (CBF) changes during nitroglycerin (NTG) triggered attacks in cluster headache patients, using arterial spin labelling (ASL).

Methods: Subjects attended screening visits to receive intravenous NTG infusion. Those for whom NTG successfully triggered attacks attended two scanning visits. They received either intravenous NTG (0.5mg/kg/min) or an equivalent amount of 0.9% sodium chloride over a 20-minute infusion. Whole-brain CBF maps were acquired using a 3 Tesla MRI scanner at "baseline" and post-infusion "headache". This study was approved by an NHS Research Ethics Committee.

Results: Eighteen subjects completed the scanning visits. In a whole-brain analysis, we identified regions of elevated CBF in the medial frontal gyrus, superior frontal gyrus, inferior frontal gyrus and cingulate gyrus, ipsilateral to the attack side, during NTG triggered attack compared with the placebo session. We also identified significantly reduced CBF in the precuneus, cuneus, superior parietal lobe and occipital lobe contralateral to the attack side. Both the hypothalamus and ipsilateral pons showed higher CBF in a region of interest analysis.

Conclusion: Increases in regional cerebral perfusion were observed in several brain regions, including the hypothalamus and pons. These data contribute to our understanding of cluster headache pathophysiology; and suggest that ASL may be valuable in future mechanistic studies of this debilitating condition.

## P0449 Study of pathogenetic mechanism chronic tension-type headache

### E. Ekusheva

#### Academy of Postgraduate Education of the Federal Research and Clinical Centre for Specialized Medical Care and Medical Technolog, Neurology and Neurorehabilitation, Moscow, Russian Federation

There are many controversial and unclear issues with chronic tension-type headache (CTTH), in particular, the pathogenetic mechanisms of the development and chronization of the disease.

**Material and methods**. 84 patients (39.2 ± 6.1) with CTTH and 25 healthy subjects comparable in age were examined. Clinico-neurological examination, nociceptive flexor reflex (RIII reflex), blink reflex, and transcranial magnetic stimulation (TMS).

**Results.** All patients with CTTH were simultaneously examined clinically and neurophysiologically before the course of therapy with drugs with proven efficacy taken at therapeutic doses for 3months. After the 3month course of treatment all patients were divided into 2 groups: 1 group (1) - with a good response to the therapy (decrease in the frequency and intensity of headache) and 2 group (2) - with minimal effect or its absence. A comparative neurophysiological analysis of the data obtained in patients of these groups before the course of preventive treatment, showed significant differences between them.

**The conclusion.** The findings of this study suggest a comprehensive neurophysiological examination prior to the course of preventive therapy and the possible use of drugs from the group of anticonvulsants in the hyperexcitability of neurons of the motor cortex, especially in the refractory course of CTTN, however, these issues require further study.

## P0450 Altered cortical inhibition after sleep restriction in interictal migraine, a blinded longitudinal study of cortical silent period

### M. S. Mykland^1^, M. Uglem^1,2^, J. P. Neverdahl^1^, T. W. Meisingset^1,2^, L. R. Øie^1,2^, T. Sand^1,2^, P. M. Omland^1,2^

#### ^1^Norwegian University of Science and Technology, Department of Neuromedicine and Movement Science, Trondheim, Norway; ^2^St. Olavs Hospital, Department of Neurology and Clinical Neurophysiology, Trondheim, Norway

##### **Correspondence:** M. S. Mykland

There is a well-known but unexplained association between sleep and migraine. In this blinded longitudinal study, we measured the effect of sleep restriction on Cortical Silent Period (CSP) as a measure of intracortical inhibition.

Fifty-five episodic migraine patients and 30 controls underwent two sessions of Transcranial Magnetic Stimulation (TMS). A 24-hour limit for pre- and postictal phase left 47 migraine patients with at least one interictal recording. Every session was preceded by two nights of either sleep restriction (4 hours) or normal sleep (8 hours) in randomized order. CSP was recorded from the abductor pollicis brevis muscle during approximately 50% of maximum muscle force with TMS intensity of 120% of the resting motor threshold.

We used a linear mixed model of CSP with sleep condition and diagnose as fixed effects and found a trend for interaction between sleep condition and diagnosis (95% CI -24.0, 0.4; p = 0.058). The corresponding interaction was significant when achieved sleep time replaced sleep condition in the model (p=0.034). Controls had an increase in mean CSP duration from normal sleep (137.3 ms) to sleep restriction (141.7 ms), while interictal migraine had an opposite pattern of decrease in CSP from normal sleep (147.8 ms) to sleep restriction (140.3 ms).

These findings indicate that migraine pathophysiology may encompass a dysfunction of the cortical regulatory response to sleep restriction involving altered GABA mediated cortical inhibition.

## P0451 Unique brainstem and hypothalamic activity preceding a migraine headache

### N. Meylakh, K. Marciszewski, L. Henderson

#### University of Sydney, School of Medical Sciences, Sydney, Australia

##### **Correspondence:** N. Meylakh

**Objective:** Although mechanisms underlying migraine pathogenesis remain hotly debated, there is a growing body of evidence suggesting that brain function alters dramatically within the 24 hours preceding a migraine headache. It is possible that altered function, particularly in brainstem and hypothalamic sites, may either trigger or facilitate a peripheral trigger to activate higher cortical areas evoking pain. The aim of this series of investigations was to determine brainstem and hypothalamic function in the 24 hours preceding a migraine, in both a cross-sectional and longitudinal study.

**Methods:** In 8 migraineurs preceding (within 24 hours) a migraine and 78 pain-free controls, and in 3 migraineurs and 5 pain-free controls, we measured resting blood oxygen level dependent functional magnetic resonance imaging (fMRI) (180 volumes, TR=2 seconds) over the entire brain.

**Results:** There was significant increased infra-slow oscillatory activity in brainstem regions encompassing the spinal trigeminal nucleus and dorsal pons, as well as the hypothalamus in the 24-hour period preceding a migraine headache between individuals. Interestingly, alterations in these brainstem sites were found in the same period within individual migraine cycles.

**Conclusion:** These findings provide evidence that in the 24-hour lead up to a migraine, the activity of the hypothalamus and brainstem is disturbed. How these regions are involved in migraine initiation and expression are yet to be fully understood.

## P0452 Cerebral cortical activity in migraine: a LORETA analysis

### M. Filipchuk, T. Castro Zamparella, M. Carpinella, D. M. Conci Magris, M. Lisicki

#### Conci Carpinella Institute, Neuroscience Unit, Córdoba, Argentina

##### **Correspondence:** M. Filipchuk

OBJECTIVE*:* To evaluate cortical activity in episodic and chronic migraine patients compared to healthy controls.

METHODS: One-minute artifact free resting-state electroencephalogram segments from 25 healthy controls and 74 migraine patients (25 ictal, 25 inter-ictal, and 24 chronic) were analyzed using eLORETA. Subject-normalized delta (1-3Hz), theta (4-7Hz), alpha (8-12Hz), beta (13-30Hz) and gamma (31-45Hz) cerebral activity was compared (whole brain, voxel-wise) between groups. Afterwards, activity from specific ROIs (data driven) was extracted to build statistical models.

RESULTS: Marked differences in resting-state cerebral activity were consistently observed in the sub-callosal (BA25), parahippocampal (BA28, BA34, BA35, BA36), and precuneus (BA7) regions. A multivariate statistical comparison showed significant differences for the interaction group-region-frequency band.

CONCLUSION: Electrophysiological differences in resting-state cortical activity between healthy controls and migraine patients involve several regions, some of which have been previously linked to the disease under alternative approaches. In contrast with other neuroimaging tools, eLORETA directly evaluates brain functioning and not indirect markers like blood flow or metabolism. In addition, band-specific information could provide valuable clues regarding different levels of neural hierarchy. Further research is required to help explain the pathophysiological significance of these observations.

**Fig. 1 (abstract P0452).**
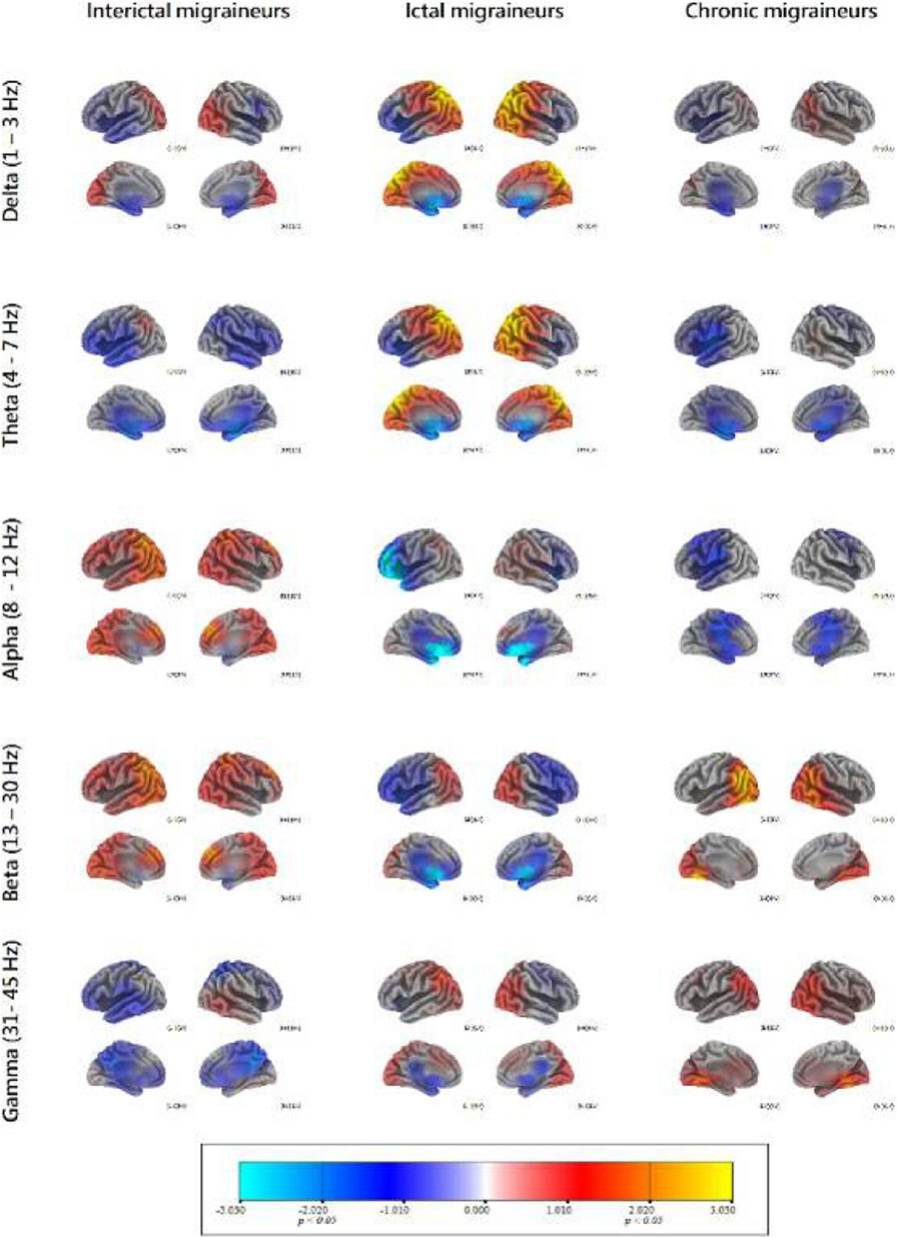
See text for description

## P0453 In migraine with aura patients visual stimulation can induce greater involvement of the salience network with the executive network, in relation to the frequency of the aura

### I. Corbelli^1^, A. Chiappiniello^2^, A. Di Renzo^3^, G. Guercini^4^, R. Tarducci^2^, P. Calabresi^5^, P. Sarchielli^1^, G. Coppola^6^

#### ^1^S.M. Misericordia Hospital - University of Perugia, Department of Medicine, Perugia, Italy; ^2^Azienda Ospedaliera di Perugia, Medical Physics Department, Perugia, Italy; ^3^IRCCS Fondazione Bietti, Rome, Italy; ^4^ServiOspedale S.M. Misericordia, Università degli Studi di Perugia, Servizio di Neuroradiologia, Perugia, Italy; ^5^Università Cattolica del Sacro Cuore, Institute of Neurology, Rome, Italy; ^6^University of Rome Polo Pontino, Department of Medico-Surgical Sciences and Biotechnologies, Latina, Italy

##### **Correspondence:** G. Coppola

Background. An involvement of the visual system in the pathophysiology of migraine with aura (MA), is well known and observed in both neurophysiological and functional neuroimaging studies. With this last method in particular, some studies have seen an involvement of different extrastriate networks during visual stimulation.

Methods. We acquired functional MRI at rest in 21 MA patients and 18 healthy controls (HCs) before and after 4 minutes of visual stimulation. For each group we extracted independent resting-state networks correlating the change in network connectivity strength with clinical disease variables.

Results. In HCs, visual stimulation significantly increases functional connectivity between the independent components pair left dorsal attention system (DAS) and executive control network (ECN), and between right DAS and ECN. In MA patients, visual stimulation significantly increased functional connectivity between the independent components pair salience network (SN) and left DAS, and between left DAS and ECN. Correlation test reveals that after light stimulation the slope of the regression line between pre and post visual stimulation Z-scores of the ECN correlated negatively with the monthly frequency of aura.

Conclusions. In HCs visual stimulation involves more the attentional and executive systems, while in MA patients, visual stimulation also involves the SN with the executive one, that is considered a function of the average monthly frequency of the aura.

## P0454 Structural brain changes in migraine correlate with migraine burden

### E. Caronna^1^, V. Gallardo López^1^, D. Pareto^2^, A. Alpuente^1^, M. Torres-Ferrus^1^, A. Rovira^2^, P. Pozo-Rosich^1^

#### ^1^Vall d’Hebron University Hospital, Neurology, Barcelona, Spain; ^2^Vall d’Hebron University Hospital, Neuroradiology, Barcelona, Spain

##### **Correspondence:** E. Caronna

**Objective**

The objective was to describe the differences between patients with migraine and healthy controls (HC) in the total amount of accumulated structural brain changes and their correlation with specific clinical features of the disease.

**Methods**

We included right-handed patients with migraine (chronic / high frequency episodic) and HC who underwent a 3T brain MRI study. Cortical thickness values were analyzed for each area (31 per hemisphere), calculating the corresponding Z-scores and defining the areas with threshold -/+ 1.96 as abnormal. We compared the two groups and we analyzed the correlations between abnormal areas and migraine clinical variables.

**Results**

We included 26 patients with migraine and 26 HC, with no differences in age, gender, anxiety/depression. In the migraine group, we observed a higher proportion of brain structural changes (migraine 14.5% vs. 4.0% HC, p<0.0001). The areas with significant differences between groups were: paracentral (p<0.0001), isthmus cingulate (p<0.0001), postcentral (p=0.006), inferior parietal (p=0.005) and parahippocampal (p=0.006). The presence of isthmus cingulate changes was associated with longer disease evolution (p=0.017) and longer chronification (p=0.013).

**Conclusions**

Migraine correlates with a greater load of structural brain changes as a marker of disease burden.

## P0455 Galcanezumab effectiveness in preclinical models of migraine and trigeminal autonomic cephalalgias

### M. Vila-Pueyo^1^, K. Johnson^2^, P. J. Goadsby^1^, P. R. Holland^1^

#### ^1^King’s College, Wolfson Centre for Age Related Diseases, London, United Kingdom; ^2^Eli Lilly and Company, Indianapolis, IN, United States

##### **Correspondence:** M. Vila-Pueyo

**OBJECTIVE**

Galcanezumab binds to and inhibits calcitonin gene-related peptide (CGRP) signalling and is approved for migraine and cluster headache treatment. To understand better its mechanisms of action, we have studied the effects of galcanezumab in preclinical models of migraine and trigeminal autonomic cephalalgias (TACs) including cluster headache.

**METHODS**

Male Sprague-Dawley rats (*N*=32) were anesthetized with isoflurane and maintained with propofol infusion (33-50mg/kg/h). Trigeminovascular afferents and the trigeminal autonomic system were activated by electrical stimulation of the meningeal afferents surrounding the dural vasculature or the superior salivatory nucleus (SSN), respectively. Neuronal responses were recorded in the trigeminocervical complex (TCC). Following baseline responses, animals were intravenously infused with human control IgG or galcanezumab (10mg/kg) and responses were recorded for 270 mins.

**RESULTS**

Treatment with galcanezumab significantly inhibited spontaneous neuronal activity in the TCC from 3h after administration (*F*_2.8,39_=8.69,*P*<0.05). Additionally, galcanezumab significantly reduced durovascular-evoked nociceptive activation in the TCC (*F*_5.4,76_=26.47,*P*<0.05), starting 150 mins post-infusion. Finally, galcanezumab significantly reduced SSN-evoked nociceptive activation in the TCC (*F*_3.7,48_=5.44,*P*<0.05), starting at 225 mins post infusion.

**CONCLUSION**

The results demonstrate a clear effect of galcanezumab in preclinical models of migraine and TACs.

## P0456 Noradrenergic networks modulating migraine-associated nociception

### P. Sureda-Gibert^1^, M. Vila-Pueyo^1^, S. Hirschberg^2^, T. Pickering^2^, P. J. Goadsby^1,3^, P. R. Holland^1^

#### ^1^King’s College, Wolfson Centre for Age-Related Diseases, London, United Kingdom; ^2^University of Bristol, School of Physiology & Pharmacology, Bristol, United Kingdom; ^3^King’s College, NIHR-Wellcome King's Clinical Research Facility (CRF), London, United Kingdom

##### **Correspondence:** P. Sureda-Gibert

**Background and objective:** Migraine patients commonly report head pain, marked fatigue and lack of concentration as hallmarks of their attacks. Noradrenergic locus coeruleus (LC) activity has been shown to have divergent roles on nociceptive durovascular-evoked neuronal responses in the trigeminocervical complex (TCC). We sought to optimise a chemogenetic strategy using a Canine adenoviral vector (CAV) with the PRS promoter (CAV2-PRS-hM3D(Gq)-mCherry) to selectively target noradrenergic projections from the LC, and investigate their role on teminal nociceptive processing. **Methods:** Twenty 4-week-old rats underwent targeted unilateral stereotaxic administration into the LC of either an active CAV2-PRS-hM3D(Gq)-mCherry (n=11) or a control non-PRS CAV (CAV-CMV-mCherry)(n=9). Allowing three weeks to recover, dural nociceptive-evoked neural responses and spontaneous activity were measured in the spinal cord TCC C1 level. Responses were assessed before and after administration of a specific chemogenetic ligand: Clozapine-N-Oxide (CNO) (3mg/kg). **Results:** Dural-evoked responses significantly decreased in the active group (p=0.0042), at time points: 60, 90 and 150min post-CNO, but not in the control group (p=0.098). Spontaneous activity significantly decreased in the active group (p=0.0391) post-CNO, but not within the control group(p=0.7303). **Conclusions:** *In vivo* activation of LC Noradrenergic projections uncovered an inhibitory influence over trigeminal/migraine-associated nuclei.

**Fig. 1 (abstract P0456).**
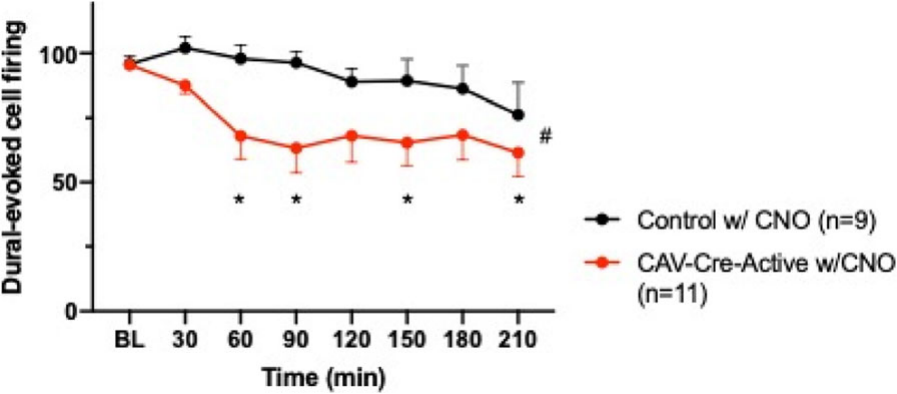
See text for description

## P0457 Medication overuse headache: Is there a difference between naproxen and paracetamol?

### D. Moreno-Ajona, M. D. Villar-Martínez, N. Futter, J. Hoffmann, P. J. Goadsby

#### King’s College, NIHR-Wellcome Trust King’s Clinical Research Facility, King’s College London, London, United Kingdom

##### **Correspondence:** D. Moreno-Ajona

Objectives

Medication overuse headache (MOH) is a cause of chronic daily headache. MOH biological explanation is unresolved. ICHD-3 defines medication overuse as an intake of more than 10 days of opioids, more than 15 days of paracetamol or NSAIDs, or more than 10 days of combined analgesics. We sought to explore medication overuse in rheumatology patients to examine links between headache and the regular intake of analgesics.

Methods

Data from patients seen at the Rheumatology Clinic, King"s College London, were retrospectively analysed. Clinical letters were checked for the presence of headache and regular intake of opioids, paracetamol and naproxen. Headache prevalence was compared using the Pearson"s chi-squared test.

Results

Data was found for 9288 patients. The total prevalence of headache was 11% (1025 patients). After exclusion of patients taking combined analgesics (2789), the prevalence of headache in patients taking regular opioids (566) was 15.7%, 15.3% for paracetamol (850) and 7.2% for naproxen (805). The difference with the total headache prevalence was significant for all groups (Chi2 opioids vs total: P=0.001; paracetamol P=0.001; naproxen: P=0.007; paracetamol vs naproxen: P<0.00001).

Conclusion

Regular intake of opioids and paracetamol was associated with higher headache prevalence. In contrast, the findings suggest that naproxen may not cause MOH and may even have a protective effect. These observations should be confirmed in a prospective clinical study.

## P0458 Effect of Strong P-gp and BCRP Inhibition, Using Cyclosporine and Quinidine as Probes, on the Pharmacokinetics of Oral Rimegepant 75 mg in Healthy Subjects

### R. Bertz^1^, M. S. Anderson^2^, J. L. Collins^1^, J. Stringfellow^3^, J. Madonia^1^, R. Bhardwaj^2^, J. A. Finley^1^, D. A. Stock^1^, V. Coric^1^, R. Croop^1^

#### ^1^Biohaven Pharmaceuticals, New Haven, CT, United States; ^2^Certara USA, Princeton, NJ, United States; ^3^Navitas Data Sciences, Pottstown, PA, United States

##### **Correspondence:** R. Bertz

**Objective**

Clinically significant drug interactions can limit the utility of medications used for the acute and preventive treatment of migraine. This study evaluated the effect of strong inhibitors of P-glycoprotein (P-gp) and breast cancer resistance protein (BCRP) on the pharmacokinetics (PK) of rimegepant in healthy adults.

**Methods**

This single-center, open-label, randomized study had 2 parts. Part 1 was a 2-period, 2-sequence, crossover evaluation of the effect of cyclosporine (1 oral 200 mg dose), a strong inhibitor of both P-gp and BCRP, on the PK of rimegepant 75 mg. Part 2 was a 2-period, 2-sequence, crossover evaluation of the effect of quinidine (1 oral 600 mg dose), a strong selective P-gp inhibitor, on the PK of rimegepant 75 mg.

**Results**

Fifteen subjects completed Part 1; 12 subjects completed Part 2. Coadministration with cyclosporine increased rimegepant AUC_0-inf_ and C_max_; geometric mean ratios (90% CI) versus rimegepant alone were 160% (149, 172) and 141% (127, 157). In Part 2, the evaluation with quinidine increased rimegepant AUC_0-inf_ and C_max_ similarly; geometric mean ratios (90% CI) versus rimegepant alone were 155% (140, 172) and 167% (146, 191).

**Conclusions**

Strong P-gp inhibitors (cyclosporine, quinidine) moderately increased rimegepant exposures (>50%, <2-fold). The similar effect of cyclosporine and quinidine on rimegepant exposure suggests that BCRP inhibition minimally influences rimegepant exposure.

**Fig. 1 (abstract P0458).**
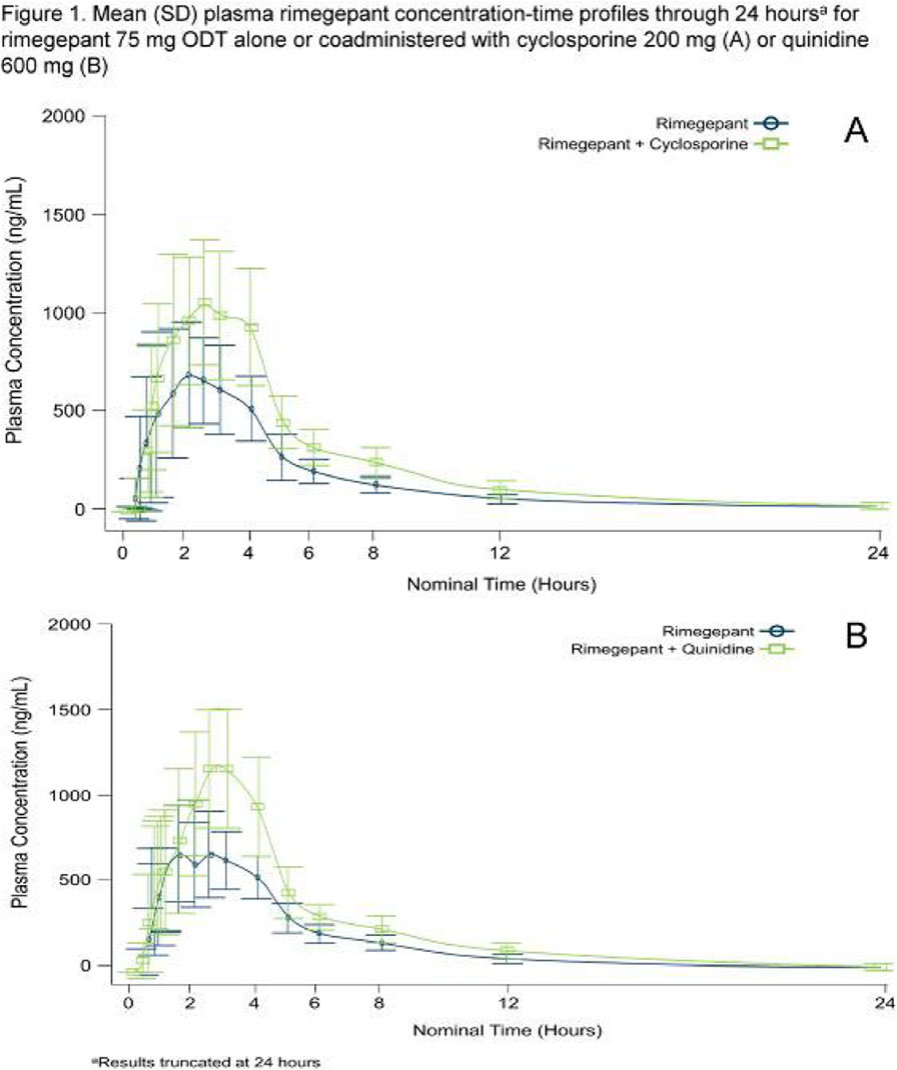
See text for description

## P0459 Use of non-invasive sensor to assess intracranial compliance in the management of migraine patients: case report

### N. Nunes Rabelo^1^, E. Peixoto^2^

#### ^1^University of São Paulo, Department of Neuroloy, São Paulo, Brazil; ^2^Federal University of São Paulo, São Paulo, Brazil

##### **Correspondence:** N. Nunes Rabelo

Objective: To report the use of a non-invasive sensor to assess intracranial compliance in the management of patients complaining of migraine. Methodology: This is a case report on the use of a non-invasive sensor to assess intracranial compliance (ICC) in a patient complaining of migraine in the late postoperative period of pseudotumor cerebri. Results: Female patient, 34 years old, diagnosed with pseudotumor cerebri, with an opening pressure initial of 60 cm H2O. Obese, a non-adjustable lumboperitoneal shunt was chosen. Two years after surgery, the patient complained of headache, a non-invasive monitoring was performed, which showed a curve with an altered ICC, a P2/P1 ratio of 1.2, suggesting a reduction in the ICC, but no change in the opening pressure, magnetic resonance imaging maintained the same previous findings and hemodynamic control remained stable. A diagnosis of migraine was made, under treatment with topiramate and propranolol, with total improvement after treatment. Conclusion: The use of non-invasive sensor helped the medical team to understand the information obtained by the ICP curve in the office and in the management of patients with headache complaints, supporting clinical decision making, improving the quality of care, correlated with underlying diseases and procedural and patient safety.

## P0460 Could erectile dysfunction be a side effect of CGRP inhibition? A case report

### L. Al-Hassany^1^, T. de Vries^1^, J. A. Carpay^2^, A. Maassen van den Brink^1^

#### ^1^Erasmus University Medical Center, Division of Vascular Medicine and Pharmacology, Department of Internal Medicine, Rotterdam, Netherlands; ^2^Tergooi Hospital, Department of Neurology, Hilversum, Netherlands

##### **Correspondence:** L. Al-Hassany

A need exists for effective preventive medication of migraine, a highly disabling disorder. Recently, antimigraine drugs targeting calcitonin gene-related peptide (CGRP) or its receptor have been approved for use in the clinic. Here, we present a case of a 54-year-old Caucasian male patient suffering from migraine with aura and who was administered a subcutaneous loading dosage of 240 mg galcanezumab, a monoclonal antibody targeting CGRP, and 120 mg each month thereafter. The patient reported erectile dysfunction (ED) as a possible side effect of the treatment. Interestingly, his potency recovered after discontinuation of the treatment. His general practitioner did not find any plausible (other) explanation for this temporary ED. Considering that CGRP is involved in the mammalian penile erection, ED is a conceivable, and possibly underreported, side effect associated with inhibition of CGRP. The reversibility of ED after cessation of galcanezumab treatment hints towards a causal association between the use of galcanezumab and ED. Yet, future studies should elucidate the actual incidence of ED in patients using drugs targeting CGRP or its receptor, and should determine whether such causal relationship between CGRP inhibition and ED exists. This would be relevant not only because of the direct sexual consequences of ED, but also considering the potential cardiovascular consequences of CGRP blockade and the association of both migraine and ED with cardiovascular disease.

## P0461 Pericranial nerve blocks with local anesthetics and steroids in chronic migraine: impact and adverse effects of the addition of dexamethasone in patients without prior response to 2 preventive treatments, experience in Colombia

### S. Taborda, F. Villa, L. Henao, V. Dominguez, Y. Giraldo

#### CES University, Neurology, Medellín, Colombia

##### **Correspondence:** S. Taborda

**Objective:** To determine the utility and real risks of adding steroids to pericranial blocks in chronic migraine.

**Methods:** Prospective, descriptive study, case series type; 22 patients with a diagnosis of chronic migraine were evaluated, who had received at least 2 first-line preventive drugs without improvement, and who were given, for the first time, a pericranial block with bupivacaine / lidocaine / dexamethasone.

**Results:** One month after intervention, there was a median difference in the average headache intensity of 2 on the visual analog pain scale (p = 0.019 confidence interval or CI: 0.4 – 3.6), the median difference in the number of days of the month with migraine before and after the block was 10 days (p = 0.001, CI 4.5 – 15-5); There was no change regarding the duration of the headache episodes, use of abortive medications, or the number of days absent from work per month. Regarding adverse effects, 75% of the patients denied any adverse effect associated, 10% reported pain for more than one day, 5% drowsiness, 5% itching, and 5% persistent dizziness. It is important to note that none of the patients reported alopecia or aesthetic alteration during follow-up.

**Conclusion:** The combination of local anesthetics with dexamethasone produced a significant decrease in the number of days with pain and in the median intensity of the headache, without producing alopecia or aesthetic alteration, the latter being a frequently feared complication due to the use of steroids.

**Fig. 1 (abstract P0461).**
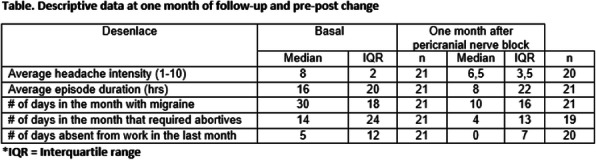
See text for description

**Fig. 2 (abstract P0461).**
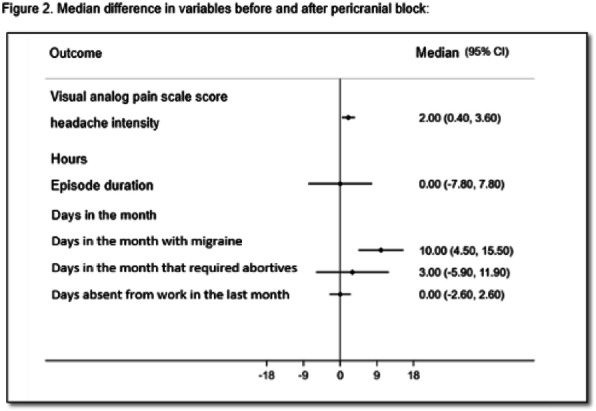
See text for description

## P0462 Migraine Preventive treatment uses and needs in a headache center from Latin America

### S. Cavanagh, V. Nagel, M. Olivier, N. Larripa, M. T. Gutiérrez, M. Grandinetti, D. Calvo, L. Bonamico, M. T. Goicochea

#### Fleni, Headache, Buenos Aires, Argentina

##### **Correspondence:** S. Cavanagh

**Backround and Objective:** Evaluate the use of preventive treatment in migraine patients at a headache clinic in Argentina.Determine how many patients needed a preventive treatment and who had failure to at least 2 preventive treatments. CGRP monoclonal antibodies were not available in Argentina at the time of this study. **Methods:** Transversal study. We evaluate adult patients with migraine for 30 consecutive days through a survey.Diagnoses,actual use of preventive,patients with indication of preventive treatment and patients who failed at least 2 preventive treatments.Study approved by ethics committee. **Results:** 602 patients with migraine diagnosis.20 were excluded.582 patients were analyzed.88% women, mean age 41 years. Diagnoses: high frequency episodic migraine (17%), chronic migraine (30%), overuse headache (13%), migraine with aura (10%). 216 (46%) patients use preventive treatment. Topiramate (42%), amitriptyline (25%), valproic acid (8%), botulinum toxin (8%), beta blockers (5%), venlafaxine (4%), flunarizine and others (3%). From the group without preventive treatment(315 patients), 138 (44%) needed one and 52% had used it before.16.5% had failure to at least 2 preventive treatments. **Conclusion:** We have a high proportion (30%) of patients with chronic migraine because we are a specialized center. Topiramate was the most used. Most of our population received or needed preventive treatment (70%) and 16.5% could benefit with new preventive treatments such as CGRP monoclonal antibody.

## P0463 Efficacy of fremanezumab in refractory chronic migraine patients: Real-world data from the Hull Migraine Clinic, UK

### F. Cheng, M. Hussain, V. Wilkinson, M. Khalil, F. Ahmed

#### Hull University Teaching Hospital, Department of Neurology, Hull, United Kingdom

##### **Correspondence:** F. Cheng

**Objective:** To evaluate fremanezumab efficacy in refractory chronic migraine (CM). **Methods:** Adult CM patients attending the Hull Migraine clinic were prescribed fremanezumab and followed up prospectively. Patients maintained a headache diary for at least 1 month prior to and continuously after commencing fremanezumab. All patients tried and failed at least 6 treatments from amitriptyline, propranolol, topiramate, candesartan, flunarizine, greater occipital nerve block and onabotulinumtoxinA. We measured monthly headache days (MHD), migraine days (MMD), headache-free days (HFD), analgesia medication (AMD) and triptan days (TD) and Headache Impact Test-6 (HIT6) scores at baseline and monthly during treatment. **Results:** 289 patients (215 F, 74 M), mean age 48.6 years (range 21–75), commenced fremanezumab between November 2020–April 2021. 182 patients (119 F, 63 M) at 3-month follow-up so far had baseline MHD, MMD and HFD of 28, 17 and 2 days, improving to 15, 6 and 15 days respectively. Mean MHD and MMD decreased by 37.7% and 57.0% from baseline (*p*<0.001). 57.7%, 38.5% and 17.0% patients achieved ≥30%, ≥50% and ≥75% MHD reduction. 79.4%, 68.1% and 41.8% achieved ≥30%, ≥50% and ≥75% MMD reduction. 55.5% and 41.8% increased baseline HFD by ≥2 and ≥3-fold. AMD and TD improved from 10 and 2, to 4 and 0 days (*p*<0.001). Mean HIT6 improved from 68.0 to 55.0 (*p*<0.001). **Conclusion:** We report significant improvement with fremanezumab as a 7th prophylactic treatment in the real-life setting.

## P0464 Effect of erenumab on central sensitization in migraine: a pilot study

### A. Berdnikova^1^, N. Latysheva^2^, E. Filatova^2^, M. Naprienko^2^, N. Kadymova^3^

#### ^1^Federal State Budgetary Educational Institution of Higher Education «A.I. Evdokimov Moscow State University of Medicine and Dent, Neurology, Moscow, Russian Federation; ^2^I.M. Sechenov First Moscow State Medical University, Neurology, Moscow, Russian Federation; ^3^Alexander Vein Headache Clinic, Moscow, Russian Federation

##### **Correspondence:** A. Berdnikova

Objective.The effect of CGRP monoclonal antibodies (MAT) may be based on the reduction of peripheral sensitization. The aim of this study is to evaluate the effect of MATon central sensitization (CS) in migraine.

Methods.We recruited 19 patients with episodic (n=6) and chronic migraine (n=13) who received 3 monthly injections of erenumab70 mg. All patients filled in the Central Sensitization Inventory (CSI), GAD-7 anxiety questionnaire, Beck Depression Inventory (BDI), and the HIT-6 disability questionnaire. Pressure pain thresholds were measured monthly at 3 sites bilaterally with the Pain Test algometer (Wagner Instruments, USA), temporal summation of pain (wind-up) - with the Neuropen (Owen Mumford, UK). Concomitant treatment of migraine (if any) remained unchanged throughout the study.

Results.Monthly headache days (MHD) decreased significantly from 20.4±9.2 to 12.0±8.6 (p=0.001), headache intensity was also reduced (p=0.0001) after 3 months of treatment. 53% of patients reached at least a 50% decrease in MHD. The level of depression but not anxiety decreased significantly (p=0.004 and p=0.27, correspondingly). The HIT-6 disability level improved dramatically (p=0.0001). We observed a significant decrease in the CSI score (p=0.04) and an increase in the pressure pain score (p=0.004).

This is the first study measuring the CSlevel during MAT treatment. The effect of erenumab on headache frequency and intensitymay be mediated by its ability to reduce the severity of CS.

## P0465 Correlation between patient-related outcome scales (PROs) and CGRP-mAbs treatment response at 3-months in migraine patients

### A. Alpuente^1,2^, M. Torres-Ferrus^1^, E. Caronna^2^, E. Gine-Cipres^3^, V. Gallardo López^2^, P. Pozo-Rosich^1,2^

#### ^1^Vall d’Hebron University Hospital, Headache Unit, Neurology Department, Barcelona, Spain; ^2^Autonomous University of Barcelona, Medicine Department, Barcelona, Spain; ^3^Vall d’Hebron University Hospital, Neurology Department, Headache Unit, Barcelona, Spain

##### **Correspondence:** A. Alpuente

**Objectives:** To analyze which patient-related outcome scale (PROs) is better correlated with an improvement in treatment response measures after 3-months.

**Methods:** Prospective study including migraine patients candidates for CGRP-mAbs. They completed an eDiary as well as PROs at baseline and after 3-months: MIDAS, HIT-6, MSQ and PGIC. Treatment response measures were reduction in monthly migraine days (MMD), monthly headache days (MHD), pain intensity (INT, from 0 to 3) and days of acute medication intake (AMD). We analyzed the correlation between PROs improvement and treatment response measures through the Spearman correlation coefficient (rs).

**Results:** 263 patients completed 3-months. All treatment response measures as well as PRO scores were significantly reduced. Only the improvement of the total score from MSQ reflected the improvement in all the treatment response measures: MHD(rs=0.243; p<0.0001); MMD(rs=0.321; p<0.0001); INT(rs=0.285; p<0.0001) and AMD(rs=0.172; p=0.004). The role function-restrictive (RFR) of the MSQ was the only domain with statistically significant correlation with all the treatment response measures.

**Conclusions:** MSQ (RFR) is the PRO significantly correlated with an improvement in all the treatment response measures after 3-months of CGRP-mAbs. It gives clues on which PROs better reflects a global improvement, which scale to use when evaluating treatment response and, probably, which domain of migraine-burden CGRP-mAbs have higher impact.

**Fig. 1 (abstract P0465).**
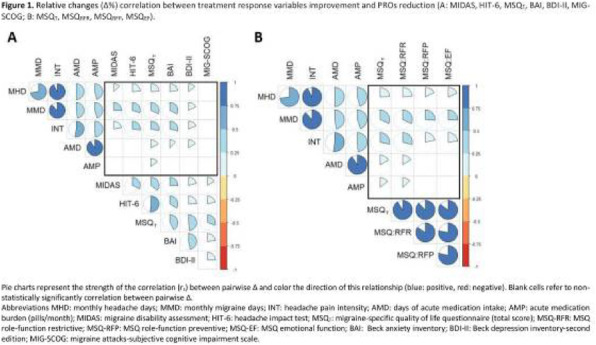
See text for description

## P0466 Which patient-related outcome scale better reflects continuing treatment with CGRP-mAbs at 3-months in migraine patients?

### A. Alpuente^1,2^, M. Torres-Ferrus^1^, E. Caronna^2^, E. Gine-Cipres^3^, V. Gallardo López^2^, P. Pozo-Rosich^1,2^

#### ^1^Vall d’Hebron University Hospital, Headache Unit, Neurology Department, Barcelona, Spain; ^2^Autonomous University of Barcelona, Medicine Department, Barcelona, Spain; ^3^Vall d’Hebron University Hospital, Neurology Department, Headache Unit, Barcelona, Spain

##### **Correspondence:** A. Alpuente

**Objectives:** To analyze which patient-related outcome scale (PROs) more accurately determines the continuation of CGRP-mAbs after 3-months.

**Methods:** Prospective study including patients candidates for CGRP-mAbs. They continuously completed an eDiary as well as different PROs at baseline and after 3- months of treatment: MIDAS, HIT-6, MSQ and PGIC. A stepwise logistic regression was used in order to identify which PROs were independently associated with treatment continuation at 3-months. A ROC analysis was performed in order to identify the most clinically valid cut-off point for the relative change (%∆) of the independent PROs identified in relation to the continuation of treatment.

**Results:** 263 patients completed 3-months and 83.3% (219/263) further continued treatment. %∆ in total MSQ (MSQT) (OR[95%]: 7.676[1.821-36.140]; p=0.047) and PGIC were the only scales being statistically significant independent factors associated with treatment continuation. The ROC analysis for the MSQT %∆ in relation to the continuation of treatment showed an AUC of 0.725 (95% CI, 0.640–0.809; p=0.003), suggesting that a mean reduction of -11.7% in MSQT significantly predicted treatment continuation.

**Conclusions:** A change in the MSQ score and PGIC scale is the most accurate way of determining the continuation of CGRP-mAbs treatment at 3-months. This finding focuses on which scale is better to manage treatment and help us decide on whether to continue treatment or not.

**Fig. 1 (abstract P0466).**
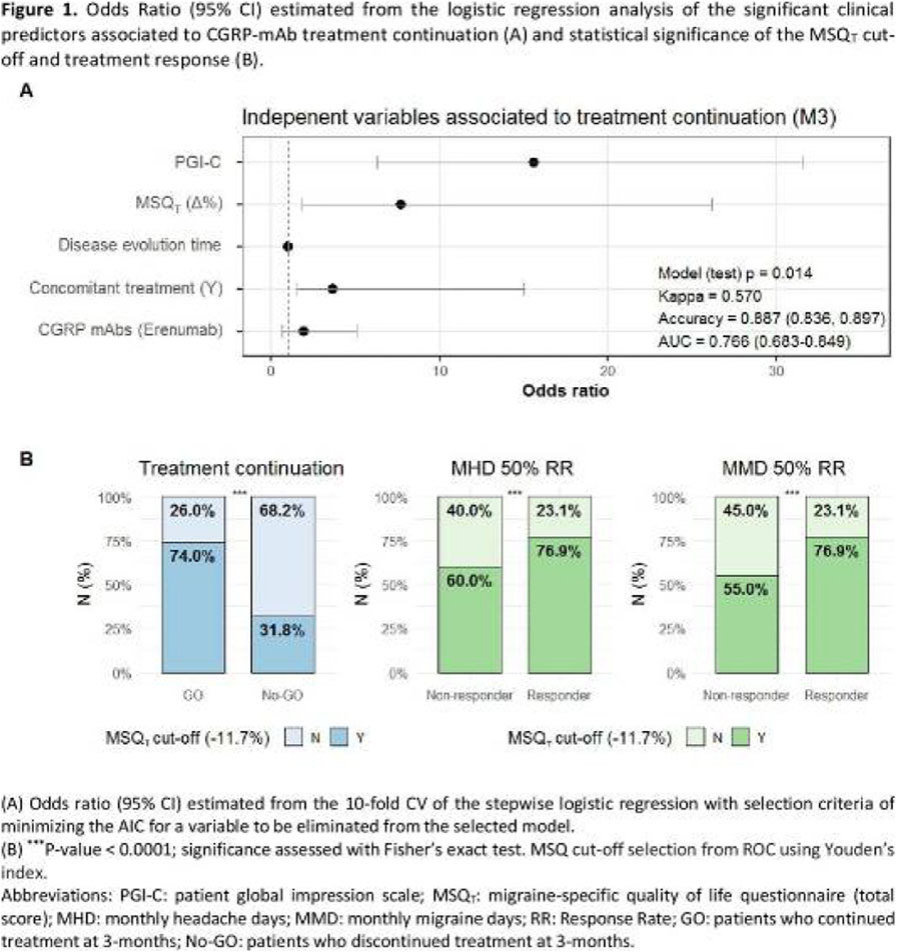
See text for description

## P0467 Patterns of response to anti-CGRP monoclonal antibodies after 6-months of treatment in resistant migraine patients

### M. Torres-Ferrus^1,2^, A. Alpuente^1,2^, E. Gine-Cipres^1,2^, E. Caronna^1,2^, V. Gallardo López^2^, P. Pozo-Rosich^1,2^

#### ^1^Vall d’Hebron University Hospital, Neurology, Barcelona, Spain; ^2^Vall d’Hebron Research Institute, Headache Research Group, Barcelona, Spain

##### **Correspondence:** M. Torres-Ferrus

**Objective:** To describe patterns of start and consistency of the response to anti-CGRP MAb after 3 and 6 months of treatment. **Methods:** We included consecutive resistant migraine patients treated with erenumab/galcanezumab. Demographic, clinical and migraine variables were collected at baseline and after 3 (M3) and 6 (M6) months of treatment. The response was categorized according to ≥50% or <50% reduction in headache days/month (HDM) and migraine days/month (MDM). We defined 4 response patterns: Maintained-Response with ≥50% reduction at M3 and M6; Short-Response with M3≥50% and M6<50% reductions, Late-Response with M3<50% and M6≥50% reductions or Partial-Response with M3 and M6<50% reductions. The baseline characteristics associated with response patterns were analyzed. **Results:** 222 patients started treatment (44 stopped treatment at M3). 178 patients were included (68.5% erenumab, 31.5% galcanezumab). Baseline frequency was 21.7±6.6 HDM and 16.2±6.7 MDM. The distribution according to response (DCM/DMM) was: Mantained-Response 36.0/48.9%; Short-Response 10.7/12.9%; Late-Response 16.3/12.4% and Partial-Response 37.1/25.8%. Short-response patients have less medication overuse (p<0.05). No other significant baseline clinical differences were found including diagnosis or the anti-CGRP MAb used. **Conclusions:** One fourth of patients do not have a consistent response to anti-CGRP MAb treatment at 3 and 6 months. Baseline characteristics do not predict the pattern of response.

## P0468 Clinical Predictors of Efficacy in the treatment with Erenumab

### M. D. Villar-Martínez^1^, D. Moreno-Ajona^1^, J. Hoffmann^1^, N. Vandenbussche^2^, P. J. Goadsby^1,3^

#### ^1^King’s College, London, United Kingdom; ^2^Ghent University Hospital, Neurology, Ghent, Belgium; ^3^University of California, Neurology, Los Angeles, CA, United States

##### **Correspondence:** M. D. Villar-Martínez

Phenotyping of primary headache disorders is an inexpensive tool, essential to diagnosis. Our aim was to identify any potential factors in clinical history that could serve as a predictor of efficacy to treatment with erenumab. Data were prospectively collected from headache patients seen at King"s College Hospital. Two authors independently reviewed patients" letters and classified them into 3 phenotype groups: pure chronic migraine and new daily persistent headache fulfilling ICHD-3 criteria, and chronic migraine-plus for those fulfilling the chronic migraine criteria that shared features with other headaches. Differences in headache days were transformed into a 57-limited-outcome-scale. Modelling was performed using negative binomial distribution with multiple imputation. The pure phenotype had lower total and severe headache days (21±8vs28±3, and 21±7vs25±10, respectively) and was predictive for reduction in total headache days, B=0.314 (0.194, 0.434, P<0.001) treated with erenumab. Longer treatment and shorter disease duration were predictors of reduction in severe headache days, B=-0.019 (-0-030, -0.007, P=0.001) and B=0.014 (0.003, 0.023, P=0.016), respectively. Reduction in headache and migraine days may be more prominent in patients with a more typical migraine phenotype. Patients with complex migraine phenotypes and new daily persistent headache may still benefit from a reduction in severe headache days, which may also be related to the duration of the treatment.

## P0469 xperience with Erenumab in Chile: An observational study

### P. Ruedi, J. Arribas

#### Instituto Chileno de Neurología, Santiago, Chile

##### **Correspondence:** J. Arribas

**Objective:** To provide the best treatment in migraine prevention we carried out a descriptive study in patients with migraine history, evaluating the response to preventive treatment with the first monoclonal antibody arrived in Chile, Erenumab, that targets CGRP receptor and has evidence of effectiveness in double blind controlled studies. Our purpose is describe the patients response and compare it with international experience in migraine treatment.

**Methods:** We considered patients older than 18 years having episodic or chronic migraine with or without aura and history of prior preventive treatment failure. The headache had to be disabling due to frequency or intensity. During the monthly 70mg Erenumab use, previous indicated prophylactic treatment was not suspended until there was no significant change in the pain course. For the effectiveness analysis we considered two objectives: the decrease in monthly migraine days (MMD) and the percentage of patients who achieved a reduction ≥50% from their MMD. For both, the comparisson was between the MMD recorded the month prior to Erenumab start and the month after the third dose.

**Results:** From 51 patients, 36 were considered on final analysis. The average decrease of MMD was 13.3 days and 75% of the patients achieved a reduction ≥50% on their initial MMD.

**Conclusion:** Even though methodological limitations, our study supports the evidence for Erenumab use as an effective migraine prevention drug, being a hope for many patients.

**Fig. 1 (abstract P0469).**
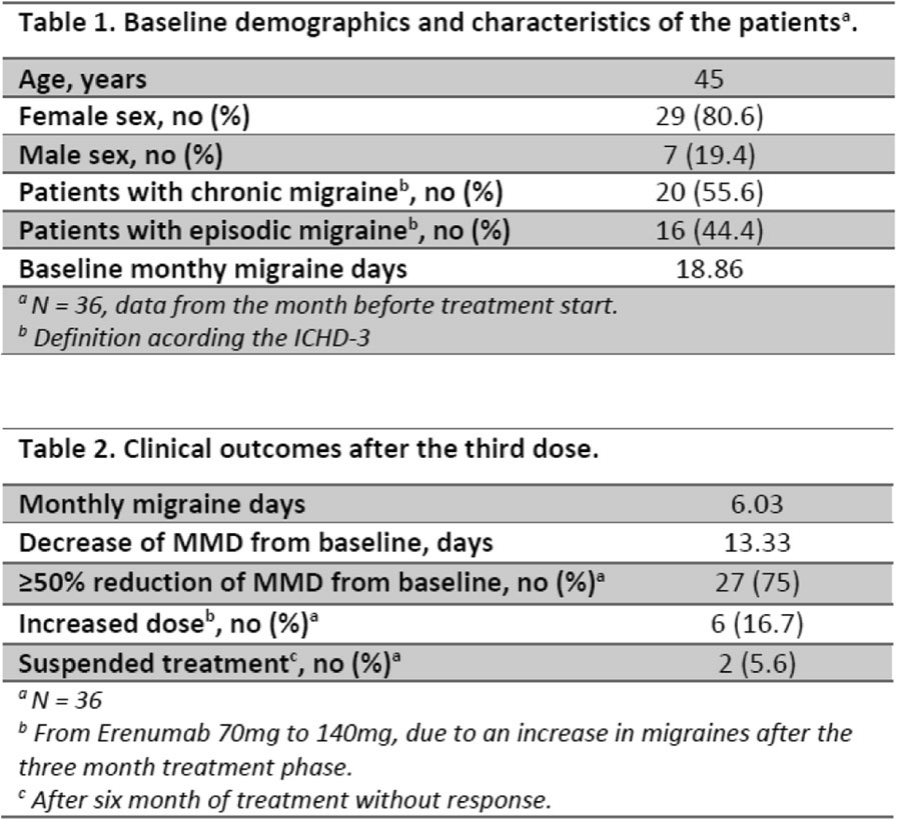
See text for description

**Fig. 2 (abstract P0469).**
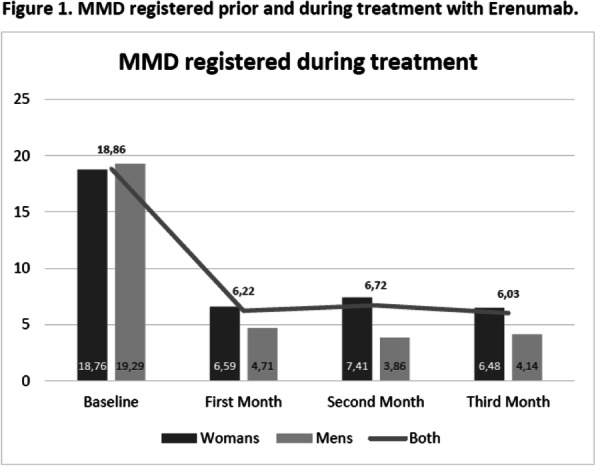
See text for description

## P0470 Ecchymoses with Erenumab

### A. R. Gonçalo Pinheiro, Â. Abreu, E. Parreira

#### Hospital Prof. Dr. Fernando Fonseca, Neurology, Lisbon, Portugal

##### **Correspondence:** A. R. Gonçalo Pinheiro

**Introduction:** CGRP is a neuropeptide with a pivotal role in the pathophysiology of migraine and may also play a role in the modulation of platelet function in humans. The experience arising from the increased real-world usage of anti-CGRP therapies leads to the description of possible new adverse effects. Recently there was a report of extensive ecchymoses in a Danish patient taking erenumab and fish oil supplements. We also describe a case of recurrent ecchymoses during erenumab treatment.

**Clinical Case:** A 40-year-old woman, with chronic migraine, after discontinuing 3 different prophylactic drugs due to adverse effects, started treatment with Erenumab 70mg. She completed 27 months of treatment with marked clinical improvement. However, on the day of erenumab injection or on the following day, after minor trauma or spontaneously, she developed ecchymoses in several body locations, unrelated to injection site. Laboratory tests, including platelet count, antiplatelet antibodies and coagulation tests were unremarkable. Despite being alerted for the potential seriousness of this adverse effect, the patient repeatedly requested to continue treatment. After stopping erenumab no new ecchymosis appeared.

**Conclusion:** Since CGRP inhibits platelet aggregation, its antagonism, through the action of calcitonin gene-related peptide receptor antibodies, can work as a transient modulator of platelet function, which may increase the risk of subcutaneous hemorrhage.

## P0471 Network Meta-analysis on Monthly Migraine Day Reductions With Fremanezumab, Rimegepant, and Atogepant in the Prevention of Episodic Migraine

### S. D. Silberstein^1^, J. M. Cohen^2^, M. Driessen^3^, B. Muresan^3^, L. J. Krasenbaum^2^, A. Johnston^4^, M. J. Seminerio^5^, K. Carr^5^, H. Akçiçek^3^, J. Dever^4^

#### ^1^Thomas Jefferson University, Jefferson Headache Center, Philadelphia, PA, United States; ^2^Teva Branded Pharmaceutical Products R&D, Inc., West Chester, PA, United States; ^3^Teva Pharmaceuticals, Amsterdam, Netherlands; ^4^Medical Decision Modeling, Indianapolis, IN, United States; ^5^Teva Pharmaceuticals USA, Inc., Parsippany, NJ, United States

##### **Correspondence:** B. Muresan

**Objective:** To assess relative efficacy for fremanezumab (225mg monthly [MLY]; 675mg quarterly [QLY]), atogepant (10/30/60mg daily [QD] or 30/60mg twice daily [BID]), and rimegepant (75mg every other day [QOD]) for prevention of episodic migraine (EM).

**Methods:** A targeted literature review (excluding studies only in patients with prior preventive failure) was conducted. A fixed-effect Bayesian network meta-analysis (NMA) indirectly compared ≥50% reduction in monthly migraine days (MMD) and mean change from baseline (CFB) in MMD at a 12-week follow-up. Relative efficacy was assessed with pairwise odds ratios (OR) and CFB differences with 95% credible intervals (CrIs).

**Results:** Six studies were included. Fremanezumab MTY showed a significantly higher ≥50% reduction in MMD vs rimegepant (OR, 2.14 [CrI, 1.38, 3.31]), significantly higher CFB reductions vs rimegepant and atogepant QD, and numerically higher CFB reductions vs atogepant BID (**Table**). Fremanezumab QLY showed a significantly higher ≥50% reduction in MMD vs rimegepant (OR, 2.03 [CrI, 1.29, 3.19]), significantly higher CFB reductions vs rimegepant and atogepant 10/30mg QD, and numerically higher CFB reductions vs atogepant BID and 60mg QD (**Table**).

**Conclusions:** Both fremanezumab doses showed better efficacy on both outcomes vs rimegepant. Fremanezumab MTY also showed significantly higher reductions in MMD vs all atogepant QD doses; fremanezumab QLY showed significantly higher reductions in MMD vs atogepant 10/30mg QD doses.

**Fig. 1 (abstract P0471).**
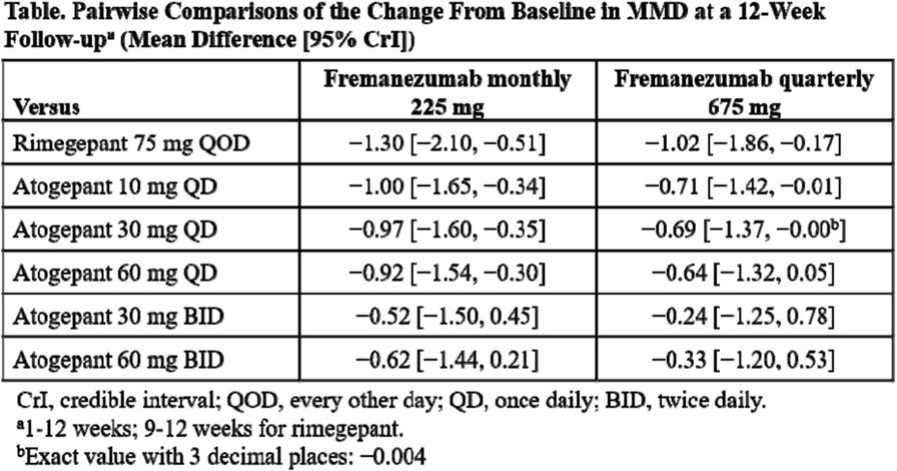
See text for description

## P0472 Clinical relapse after erenumab suspension

### A. R. Gonçalo Pinheiro, Â. Abreu, E. Parreira

#### Hospital Prof. Dr. Fernando Fonseca, Neurology, Lisbon, Portugal

##### **Correspondence:** A. R. Gonçalo Pinheiro

**Objective:** Evaluate the course of migraine after discontinuing erenumab treatment in the group of responders.

**Methods:** From a prospective study of 28 months of follow-up of erenumab treated patients in our hospital, we performed a sub-analysis of the group of responders. We recorded frequency and severity of attacks, analgesic intake and several patient reported outcome measures.

**Results:** From a total of 35 migraine patients treated with erenumab, 9 patients, that had stopped due to sustained response, were included. They had an average age of 50,2 years. Prior to erenumab treatment, 33% had chronic migraine. The median duration of treatment was 11 months ranging from 6 to 12 months. Erenumab had led to a reduction in migraine frequency of more than 50% in all of them. After discontinuing treatment, 6 patients (67%) had a progressive increase in headache frequency with 4 even reporting a possible rebound effect. 3 and 5 months after erenumab discontinuation we recorded a median increase in attack frequency of 5 and 11-days per month, respectively. In 5 patients, erenumab had to be restarted. In all 5, the second course of treatment led to a quick improvement. This positive response persisted at 6-month follow-up.

**Conclusion:** We highlight that in this group, suspension of erenumab led not only to a progressive worsening of migraine but also to a possible rebound effect. This might indicate that in this group, anti-CGRP therapy didn´t exhibit a disease-modifying effect.

## P0473 Migraine Through the Lens of The Meditating Brain: Explaining Psychophysiological Distinctions Between Meditation Categories and Their Application for Migraine Management

### F. Golshan, M. Mikleborough

#### University of Saskatchewan, Psychology, Saskatoon, Canada

##### **Correspondence:** F. Golshan

Background and Objective: Migraine is a prevalent primary headache disorder with incapacitating neurological characteristics. With respect to the disability and adversity caused by migraine by and large, there is a need for examining noninvasive treatments that can have efficacy on migraine management in the long run. Meditation is a well-recognized umbrella term including different mental training techniques which are shown to be efficient in helping individuals cultivate their core psychological capacities, such as attention and affective self-regulation, and improve resilience towards their physical pain. Based on the dichotomy of meditation into Open Monitoring and Focused Attention subgroups in the neuroscience approach, this presentation will specifically review and suggest how bottom-up and top-down modulation of migraine pain can be facilitated via the two groups of meditation. Subsequently, Open Monitoring and Focused Attention techniques are compared with regards to their attentional and affective control before and during a migraine attack. Further suggestions are made for a migraine symptom checklist in order to optimize the efficacy of the selected meditation practice.

## P0474 Evaluation of the Pharmacokinetic Interaction and Safety of Coadministered Atogepant and Topiramate

### R. Boinpally^1^, D. McGeeney^1^, L. Borbridge^2^, M. Butler^1^, L. Severt^1^

#### ^1^AbbVie, Madison, NJ, United States; ^2^AbbVie, Irvine, CA, United States

##### **Correspondence:** R. Boinpally

**Objective:** To evaluate the potential for pharmacokinetic (PK) drug-drug interactions between atogepant and topiramate.

**Methods:** Phase 1, single-center, open-label, multiple-dose study. Healthy adults (18–45 y) were randomized to cohort 1 to evaluate the effect of topiramate (mild CYP3A4 inducer) 100mg twice daily on the PK of atogepant (CYP3A4 substrate) 60mg once daily, or cohort 2 to evaluate the effect of atogepant 60mg once daily on the PK of topiramate 100mg twice daily. Blood samples were collected to evaluate the potential for PK drug-drug interactions. Safety was monitored throughout the study.

**Results:** A total of 28 and 25 participants were enrolled in cohorts 1 and 2, respectively. For atogepant, overall systemic exposure (AUC_0-tau_) and maximum plasma concentration (C_max_) were reduced by 25% and 24%, respectively, with topiramate coadministration. Atogepant median T_max_ was the same when administered alone or with topiramate (2h). For topiramate, AUC_0-tau_ and C_max_ were reduced by 5% and 6%, respectively, with atogepant coadministration. Topiramate median T_max_ was delayed by 0.5h when coadministered with atogepant. Administration of atogepant and topiramate, alone and in combination, was safe and well tolerated.

**Conclusion:** Atogepant AUC_0-tau_ and C_max_ decreased by 25% and 24% when coadministered with topiramate. Given the wide effective dose range for atogepant, these changes are not expected to be clinically significant and no dose adjustments are needed.

## P0475 Three-year efficacy and safety of erenumab in participants with episodic migraine and 2–4 prior preventive treatment failures: Results from the LIBERTY study

### U. Reuter^1,2^, P. J. Goadsby^3,4^, M. Ferrari^5^, G. Paiva da Silva Lima^6^, S. Mondal^7^, S. Wen^8^, T. Stites^8^, M. Arkuszewski^9^, M. Lanter-Minet^10,11^, S. Pandhi^9^

#### ^1^Charité University Hospital Berlin, Neurology, Berlin, Germany; ^2^Universitätsmedizin Greifswald, Greifswald, Germany; ^3^King’s College, NIHR-Wellcome Trust, London, United Kingdom; ^4^University of California, Neurology, Los Angeles, CA, United States; ^5^Leiden University Medical Center, Neurology, Leiden, Netherlands; ^6^Amgen Inc., Thousand Oaks, CA, United States; ^7^Novartis Healthcare Pvt. Ltd., Hyderabad, India; ^8^Novartis Pharmaceuticals Corporation, East Hanover, NJ, United States; ^9^Novartis Pharma AG, Basel, Switzerland; ^10^Université Côte d’Azur, Pain Department and FHU InovPain, Nice, France; ^11^Auvergne University, INSERM U1107 Migraine and Trigeminal Pain, Clermont-Ferrand, France

##### **Correspondence:** U. Reuter

**OBJECTIVE**

Efficacy of erenumab 140mg has been demonstrated in the 12-week double-blind treatment phase (DBTP) of the LIBERTY study. Efficacy and safety of erenumab at completion of the 3-year open-label extension phase (OLEP) are reported.

**METHODS**

Patients completing the DBTP (N=240) continued into OLEP, receiving monthly erenumab 140mg for ≤3 years. Outcomes measured at Week 168 were ≥50% and ≥75% reduction in monthly migraine days (MMD); change from baseline (BL) in MMD, Headache Impact Test (HIT-6™) total score, Migraine Physical Function Impact Diary (MPFID), Everyday Activities (EA), Physical Impairment (PI) and safety.

**RESULTS**

Of 240/246 (97.6%) patients entering OLEP (118 continuing erenumab, 122 switching from placebo), 169 (70.4%) completed 3-year OLEP. Discontinuations were mainly due to lack of efficacy (12.5%, n=30), patient decision (10.8%, n=26) and adverse events (AEs; 4.6%; n=11, single case per AE). The ≥50% and ≥75% responder rate at 3-year completion was 52.3% and 33.1% (Table). Mean (SD) change from BL at 3-year completion was −4.4 (3.9) in MMD. Mean (SD) change from BL at 3-year completion was –9.7 (8.9), –6.1 (8.2) and –5.1 (7.6) for HIT-6, MPFID-EA and -PI scores. Common AEs (>10%) were nasopharyngitis, influenza and back pain.

**CONCLUSIONS**

Efficacy was sustained over 3 years in patients with difficult-to-treat EM who failed 2–4 prior migraine preventives. Erenumab was well-tolerated, with no new safety signals reported after long-term exposure.

**Fig. 1 (abstract P0475).**
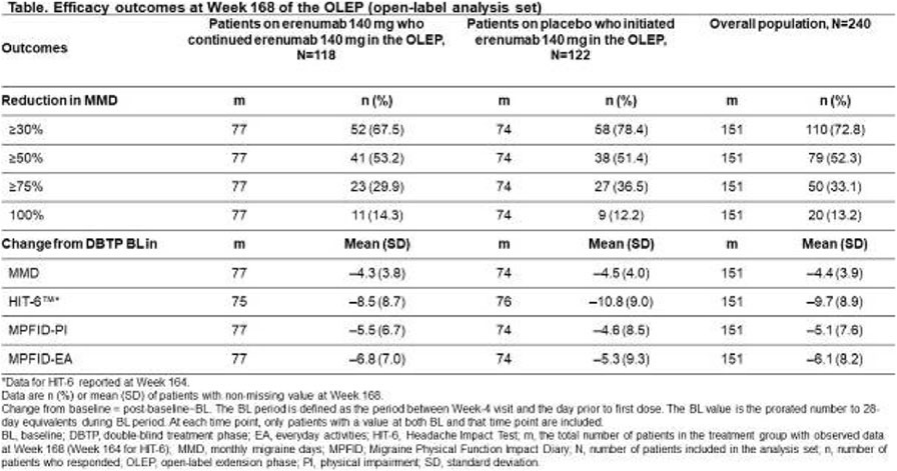
See text for description

## P0476 Long-term safety and tolerability of erenumab in episodic migraine: A pooled analysis from two clinical trials and their extension phases

### M. Ashina^1^, U. Reuter^2,3^, D. W. Dodick^4^, F. Zhang^5^, S. Ritter^6^, T. Stites^6^, G. Paiva da Silva Lima^5^, M. Arkuszewski^7^, P. J. Goadsby^8,9^

#### ^1^Rigshospitalet-Glostrup, Department of Neurology, Danish Headache Center, Faculty of Health and Medical Sciences, Glostrup, Denmark; ^2^Charité University Hospital Berlin, Department of Neurology, Berlin, Germany; ^3^Universitätsmedizin Greifswald, Greifswald, Germany; ^4^Mayo Clinic, Department of Neurology, Scottsdale, AZ, United States; ^5^Amgen Inc., Thousand Oaks, CA, United States; ^6^Novartis Pharmaceuticals Corporation, East Hanover, NJ, United States; ^7^Novartis Pharma AG, Basel, Switzerland; ^8^King’s College, NIHR-Wellcome Trust, King’s Clinical Research Facility, London, United Kingdom; ^9^University of California, Department of Neurology, Los Angeles, CA, United States

##### **Correspondence:** M. Ashina

**Background and objective:** To assess the long-term safety of erenumab using pooled data from the double-blind treatment phases (DBTP) and open-label extension phases (OLEP) of two clinical trials in episodic migraine (NCT03096834, NCT01952574). **Methods:** The incidence of adverse events (AEs) were summarized as exposure-adjusted patient incidence rates per 100 patient-years (r). Anti-erenumab antibodies were detected using a validated bridging electrochemiluminescence immunoassay. **Results:** Of 729 patients randomized across both studies, 502 received erenumab (70 or 140 mg) or placebo in the 12-week DBTP and 623 received erenumab (70 or 140 mg) in the 3- or 5-year OLEP. The cumulative duration of exposure to erenumab during the DBTP and OLEP was 54.3 and 1899.5 patient-years, respectively. Overall exposure-adjusted AE incidence rates were similar in the DBTP and OLEP; no new AEs emerged over time (**Table**). The most common AE for the erenumab treatment groups (presented as n [r], whereby n = number of subjects reporting ≥1 AE) was nasopharyngitis (DBTP, 11 [20.9]; OLEP, 224 [24.0]). The incidence of constipation (DBTP: 4 [7.5]; OLEP: 40 [3.2]) and hypertension (DBTP: 3 [5.6]; OLEP: 46 [3.7]) remained low over time. The occurrence of anti-erenumab antibodies was 5.8% in the DBTP and 10.3% in the OLEP, with a respective 0.4% and 1.4% developing neutralizing antibodies. **Conclusions:** Erenumab demonstrated a consistent favorable safety and tolerability profile with long-term exposure.

**Fig. 1 (abstract P0476).**
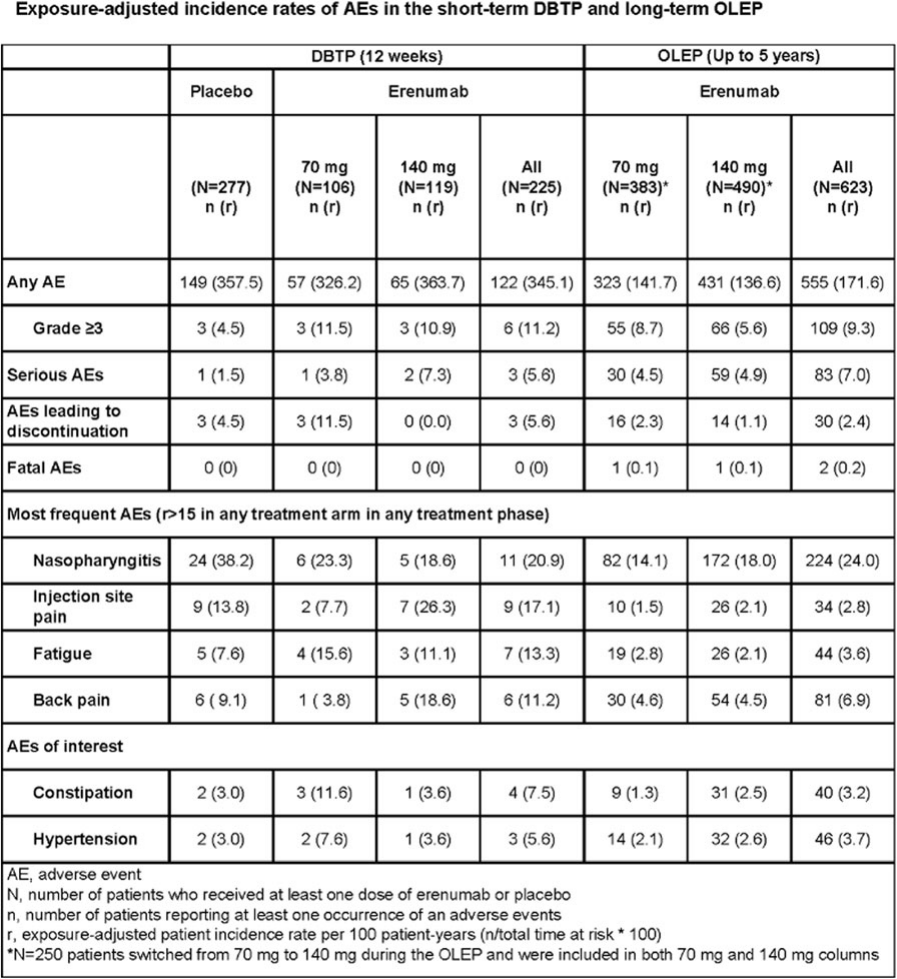
See text for description

## P0477 Effects of introvision, a self-regulation method based on a mindfulness-like perception technique in migraine prevention: a randomized waiting-list controlled study

### M. Empl^1^, S. Löser^2^, P. Spille^2^, A. Rozwadowska^3^, R. Ruscheweyh^4^, A. Straube^4^

#### ^1^Practice, Ludwig-Maximilians-University, Department of Neurology, Munich, Germany; ^2^Introvision e.V., Hamburg, Germany; ^3^kbo Klinikum, Neurology, Haar, Germany; ^4^Ludwig-Maximilians-University, Neurology, Munich, Germany

##### **Correspondence:** M. Empl

Objective: Migraine is a brain disorder with recurrent headache attacks and altered sensory processing. We examined the effect of introvision, a self-regulation method based on a mindfulness-like perception technique developed at the university Hamburg in migraine prevention. Methods: Migraineurs with at least 5 headache days per month were block-randomized to the experimental group (EG) or waiting list group (WL), the latter starting 6 weeks after the EG. Participants learned introvision in 6 weekly on-site group sessions with video-conference support followed by three individual video-conference sessions. Headache parameters were assessed before introvision and three months after the last individual introvision session. Results: 53 patients completed the study. The primary outcome, headache days of the EG after introvision compared to the WL before the introvision, showed no significant effect (11,3+/-7,8, n=22; vs. 10,9 +/- 6,2, n=29, p=0,63; Mann-Whitney-U-Test. The secondary outcome, comparing pooled EG and WL data before and after introvision, showed significant reduction of headache days (11,5+/- 6,4 vs. 9,8+/-7,0;, p=0,003; Wilcoxon-paired-Test) medication intake, HIT-6 scores and increased self-efficacy. Introvision was recommended by 97 % of participants. Conclusion: The study did not reach its primary endpoint. However, secondary outcome parameters showed an improvement of migraine after the intervention, with a decrease in monthly headache days by 1.7 days/month.

**Fig. 1 (abstract P0477).**
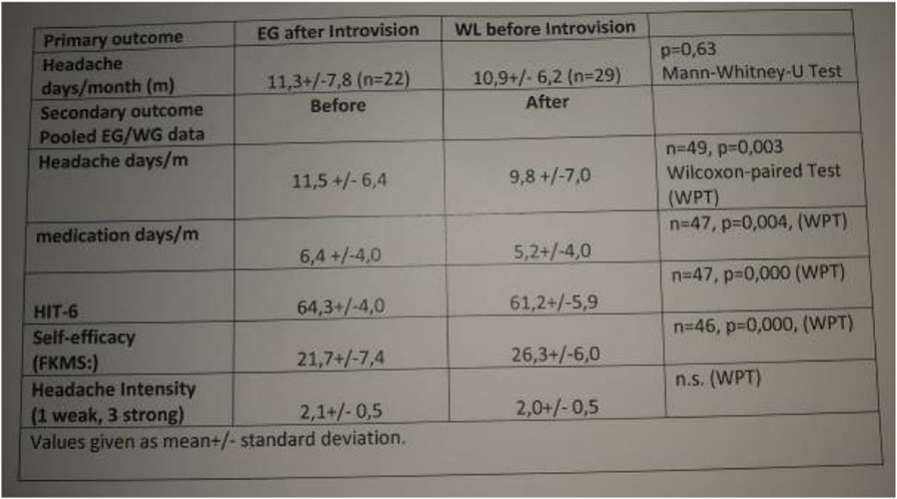
See text for description

## P0478 The Role of the HEIDI (HEadache Interactive DIary) app in Headache Services at Sunderland Royal Hospital

### G. Kennedy

#### Sunderland Royal Hospital, Neurology, Newcastle, United Kingdom

**Background and objective:**

Chronic migraine is underdiagnosed and not all patients suitable for NICE-approved therapies are identified. Headache diaries help clarify the headache diagnosis, identify eligible patients for NICE-approved treatments and recognise medication overuse. A headache diary app is likely to improve compliance with diary completion and provide a platform to monitor patients remotely which has become a requirement during the COVID pandemic restrictions and the introduction of home-based CGRP antibody treatments. This study assesses patient and clinician feedback to assess the feasibility of using the HEIDI app to support headache service s.

**Methods:**

Fifty patients receiving botulinum toxin treatment for chronic migraine were enrolled to use the HEIDI app. Patient reported outcomes on accessibility, speed, accuracy, usefulness and user satisfaction at 3 and 6 months.

**Results:**

The vast majority of patient users preferred the HEIDI app to paper diaries. The HEIDI app was associated with improved diary accessibility, data accuracy and patient satisfaction.

**Conclusions:**

The HEIDI app is a superior method to paper diaries in monitoring chronic headache conditions. It offers the unique function of remote patient monitoring which can lead to reduced inappropriate appointments and more efficient patient assessments which can be performed remotely. It is an appropriate up-to-date digital tool that can support headache services.

## P0479 Serotonin polymorphism associate to migraine subject in Malaysia

### N. A. A. Ain, W. A. W. Sulaiman

#### Universiti Putra Malaysia, 1Department of Medicine, Faculty of Medicine and Health Sciences, Serdang, Malaysia

##### **Correspondence:** N. A. A. Ain

Migraine is a disabling primary headache disorders, which has an annual prevalence of 15%. Migraine is regarded as a polygenic disease and serotonergic pathways and calcitonin gene related peptide (CGRP) appear to play a major role in its pathogenesis. We had conducted a case control study to determine the association of serotonin and CGRP polymorphism gene with migraine among a multi-ethnic Malaysian population. The gene polymorphisms were analysed in 113 migraine patients and 163 control subjects. Serotonin and CGRP polymorphism were genotyped by polymerase chain reaction-restriction fragment length polymorphism (PCR-RFLP) method. The study found that there were significant difference between serotonin and CGRP polymorphism among individuals with migraine as compared to the control (p<0.005). Hence, the study support possibility of involvement of the serotonin and CGRP in migraine among multi-ethnic Asian population in Malaysia.

## P0480 Understanding the patient experience and disease burden of migraine: a qualitative interview study

### K. Gallop^1^, S. H. Lo^1^, K. Shepard^1^, L. Powell^2^, T. Smith^3^, L. Harris^4^, G. L'Italien^4^

#### ^1^Acaster Lloyd Consulting Ltd., London, United Kingdom; ^2^Broadstreet Health Economics & Outcomes Research, Vancouver, Canada; ^3^StudyMetrix Research, Missouri, United States; ^4^Biohaven Pharmaceuticals, Connecticut, CT, United States

##### **Correspondence:** K. Gallop

Little is known about the interictal (between attack) burden of migraine. Despite many treatment options, patients are often not satisfied with treatment. This study explored the impact of migraines on patients" quality of life (QoL), including the interictal burden, and patients" treatment satisfaction. Semi-structured interviews were conducted with migraine patients in the US, UK and Canada. Interviews explored migraine symptoms, QoL impact and treatment satisfaction. Qualitative data were analysed using thematic analysis. Participants (n=35) had migraines on average 12 (SD: 8; range 1-30) days per month, reporting impacts of migraine attacks on daily life, work/study, emotional wellbeing, social/leisure activities and relationships. The interictal burden included lifestyle changes, being unable to plan, reducing/stopping work, avoiding migraine triggers and feeling anxious about migraines. Participants discussed their treatment experience and satisfaction, including efficacy, side effects, treatment administration and convenience. Most used both acute and preventative treatments and, when prompted, 30 were interested in exploring the use of a single medication for both purposes. Among participants with experience of injectable preventative treatment, some reported wearing off effects between injections and reduced efficacy over time. This study highlights the burden of migraines both during and between attacks and underlines the unmet treatment needs of migraine patients.

## P0481 Chronic headache controlled by surgical resection of underlying pulmonary arteriovenous malformation: Hereditary Hemorrhagic Telangiectasia

### E. J. Choi^1^, C. A. Lim^2^, D. G. Lee^2^

#### ^1^Gumi Hospital, Soonchunhyang University, Neurology, Gumi, South Korea; ^2^University of British Columbia, Neurology, Vancouver, Canada

##### **Correspondence:** E. J. Choi

Objective: Hereditary Hemorrhagic Telangiectasia (HHT) is an autosomal dominant disorder characterized by abnormal vascular growths leading to arteriovenous malformations (AVM) in various organs. Pulmonary AVM is a common presentation of HHT which can result into pulmonary arteriovenous fistula leading to neurologic complications such as chronic headache, brain abscess and meningitis. We report a case of silent pulmonary AVM leading to brain abscess in a previously undiagnosed HHT patient. Methods: This is a case of a pulmonary AVM and concomitant frontoparietal brain abscess in a 48-year-old female with HHT treated with antibiotics and wedge resection. Results: A 48-year-old female with chronic headache presents with epistaxis and right leg weakness. Familial history was suspicious for HHT with the imaging findings. CT Chest revealed a right lower lung AVM and brain MRI showed a large frontoparietal brain abscess. The brain abscess was treated with antibiotics and the pulmonary AVM surgically managed with wedge resection. The patient made complete neurological recovery after initiation of appropriate antibiotics. Conclusions: The patient above had chronic headahce with recurrent epistaxis but otherwise a silent pulmonary AVM leading to brain abscess and therefore, presentation of neurologic symptoms. this case illustrates the significance of screening for pulmonary AVM in patients with HHT suggestive symptoms to prevent further neurological sequelae.

**Fig. 1 (abstract P0481).**
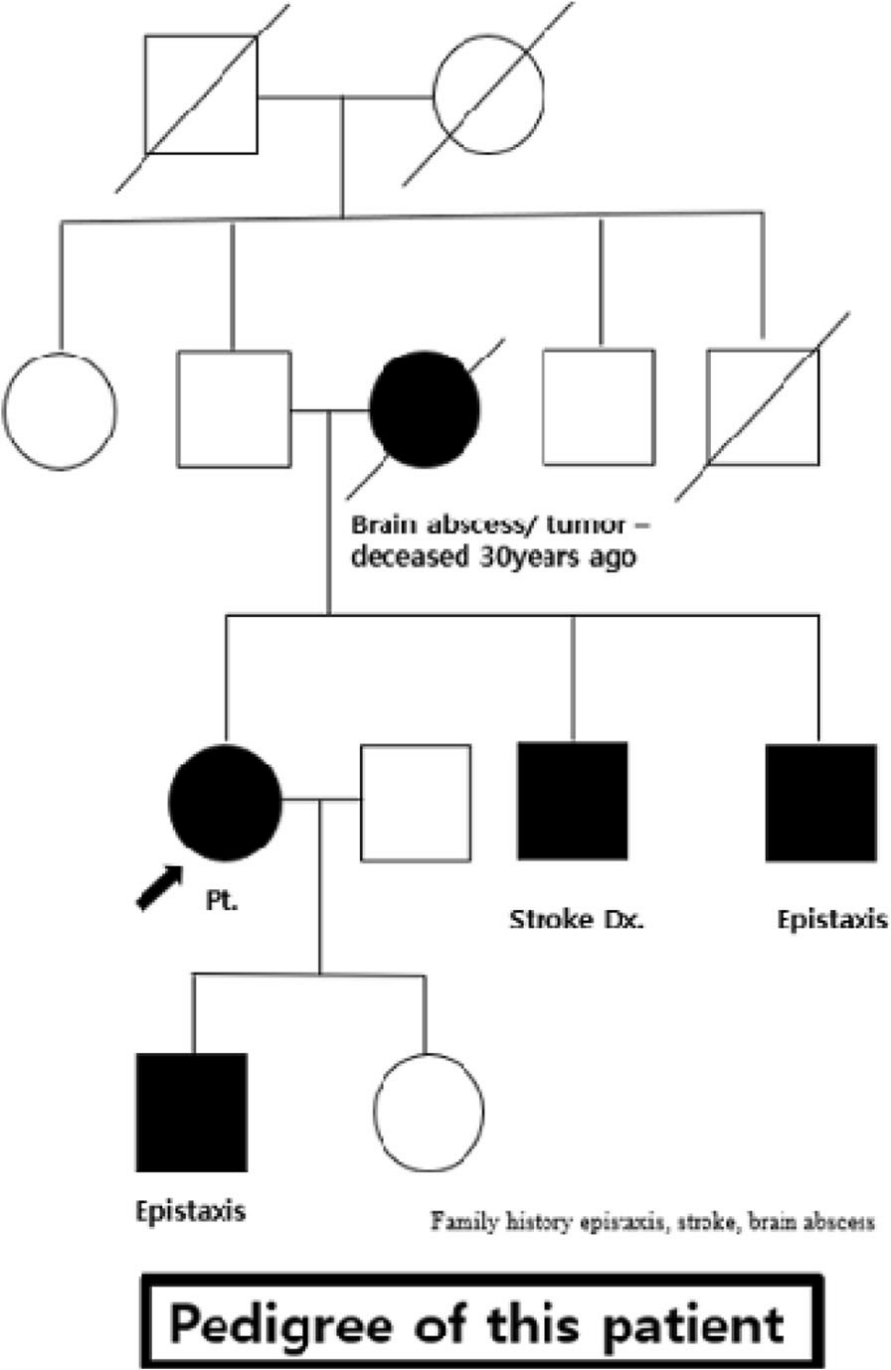
See text for description

**Fig. 2 (abstract P0481).**
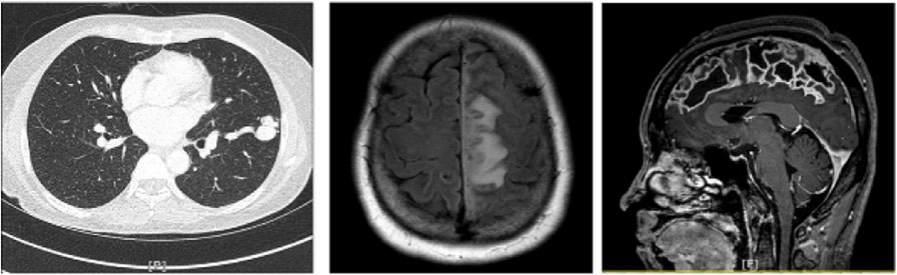
See text for description

## P0482 MIGRANEA AS A DIFFERENTIAL DIAGNOSIS OF CADASIL IN YOUNG INDIVIDUALS

### W. Horta, M. Horta

#### University of Pernambuco, Department of Neurology, Recife, Brazil

##### **Correspondence:** W. Horta

The most prevalent hereditary cerebral angiopathy is the cerebral autosomal dominant arteriopathy with subcortical infarcts and leukoencephalopathy, known worldwide as (CADASIL). It is a disease caused by a mutation in the NOTCH3 gene on chromosome 19. Objective: To identify migraine as an initial symptom of CADASIL. Method: Patients diagnosed with CADASIL Results: The proportion of migraine in patients with CADASIL is 5 times higher than the general population. The first symptoms usually occur before the age of 20, with the average age of appearance being 30. Migraine precedes other symptoms by many years, and resonance images may still be normal, leading to delays and diagnostic errors. The relationship between migraine and CADASIL can be justified by the identification of genetic abnormalities that occur on chromosome 19, the same chromosome as familial hemiplegic migraine. The characteristic of CADASIL migraine is the presence of aura symptoms, the most prevalent being the visual symptoms, followed by the sensory ones. Some individuals have atypical auras. A possible mechanism for the increased prevalence of aura in patients with CADASIL is the increased susceptibility to cortical spreading depression. Conclusion: Migraine, especially if there are symptoms of aura, can be an initial symptom of CADASIL disease, and should be considered as a differential diagnosis, especially when dealing with younger individuals.

## P0483 Psychiatric Co-Morbidities in Idiopathic Intracranial Hypertension

### J. Juhl Korsbaek^1^, D. Beier^2^, L. Dehghani Molander^3^, S. Malm Hagen^4^, R. H. Jensen^1^

#### ^1^Danish Headache Center, Department of Neurology, Rigshospitalet-Glostrup, Glostrup, Denmark; ^2^Odense University Hospital, Department of Neurology, Odense, Denmark; ^3^Odense University Hospital, Department of Ophthalmology, Odense, Denmark; ^4^Rigshospitalet-Glostrup, Department of Ophthalmology, Glostrup, Denmark

##### **Correspondence:** J. Juhl Korsbaek

**Objective**

To investigate the prevalence of psychiatric disease in patients with idiopathic intracranial hypertension (IIH).

**Methods**

This study is a prospective cohort study of 111 patients with new-onset IIH. A structured medical history was taken, and the prevalence of psychiatric disease was compared with the age and sex specific prevalence in the population. We compared IIH without psychiatric disease (IIH-P) and IIH with psychiatric disease (IIH+P) at baseline and 6 months for: BMI, visual fields (perimetric mean deviation), headache, employment and surgical intervention.

**Results**

In total, 45% of IIH patients had a psychiatric co-morbidity. Major depressive disorder (24.3% in IIH, 1.8-3.3% in the population) and emotionally unstable personality disorder (6.3% in IIH, 2.1% in the population) were highly prevalent compared to the general population. Visual fields were poorer in IIH+P at baseline (-8.6 vs. -6.0, p = 0.02) and 6 months (-5.5 vs. -4.0, p < 0.01). Medication related to weight gain was used by 28% of IIH+P (3.3% of IIH-P, p < 0.001).

**Conclusion**

Psychiatric co-morbidities, particularly major depressive disorder and emotionally unstable personality disorder, are highly prevalent in IIH. Patients with IIH+P have significantly worse visual fields at baseline and 6 months. We speculate that co-morbid psychiatric disease is related to underlying disease pathophysiology or exposure to medication. A reasonable approach is to include psychiatrists in IIH-teams.

## P0484 Intracranial Vertebral Artery Dissection Chiefly Presenting with Stabbing Headache

### B. S. Kim

#### Bundang Jesaeng General Hospital, Neurology, Seongnam, South Korea

Backgrounds

Despite the fact that most stabbing headaches are primary, stabbing headache could be occasionally a main manifestation of serious intracranial abnormalities.

Case

A 56-year old man visited our outpatient headache clinic due to newly developed stabbing headache in the right posterior head 4 days ago and following ipsilateral tinnitus and decreased hearing 2 days ago. Audiograms of the patient revealed sensorineural hearing loss of ≥ 30dB in the right audiogram, suggestive of sudden sensorineural hearing loss (SSNHL). Therefore, he undertook brain magnetic resonance imaging (MRI) to investigate intracranial pathology related to his SSNHL. Diffusion-weighted imaging showed multiple acute infarctions in the right posterior cerebral artery and superior cerebellar artery territories. In perfusion MRI, there was an extensive perfusion delay in the cerebellum and mid to lower brainstem. Magnetic resonance angiography and conventional angiography showed tapered thrombotic occlusion between right V3 segment of the vertebral artery and mid-basilar artery. After anticoagulation therapy using intravenous heparin, there were no new symptoms, and his tinnitus and decreased hearing improved.

Conclusions

This is an uncommon case of secondary stabbing headache accompanying with ear symptoms due to acute intracranial VAD. We should be careful of such occasional presentation of secondary stabbing headaches in clinical practice.

**Fig. 1 (abstract P0484).**
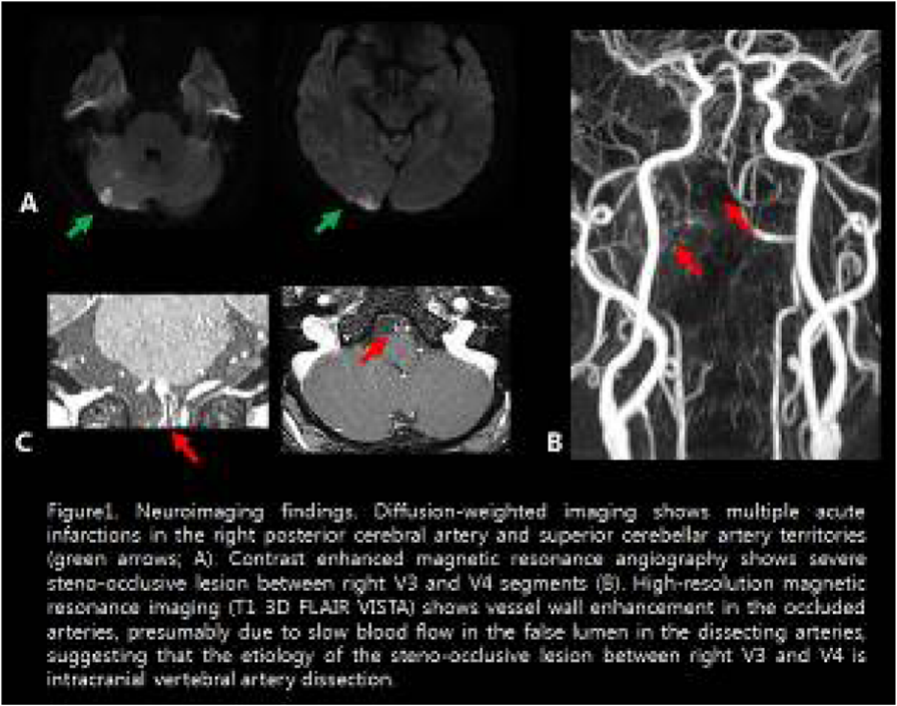
See text for description

**Fig. 2 (abstract P0484).**
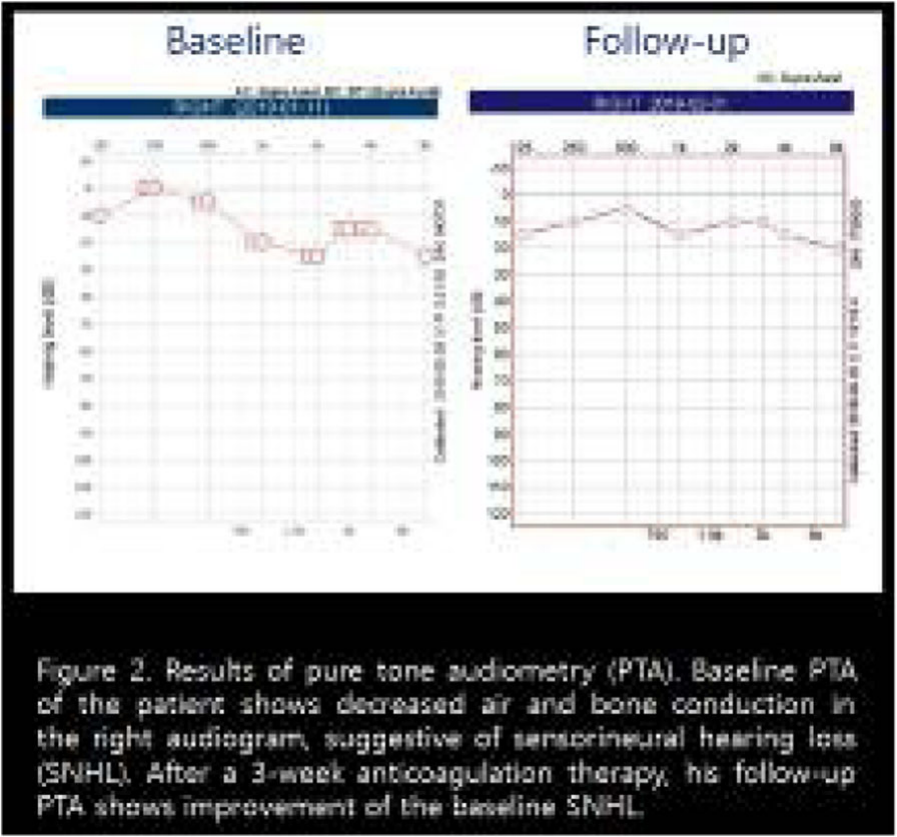
See text for description

## P0485 ACT for Migraine: Effect of Acceptance and Commitment Therapy (ACT) for High Frequency Episodic Migraine without Aura: A phase-II, multicentric, randomized, open-label study

### L. Grazzi^1^, C. Bernstein^2^, E. Sansone^1^, A. Raggi^1^, E. Grignani^1^, M. Searl^2^, F. Andrasik^3^, P. Rizzoli^2^

#### ^1^IRCCS Foundation “Carlo Besta” Neurological Institute, Neuroalgology, Milan, Italy; ^2^Harvard Medical School, J Graham Headache Center, Brigham & Faulkner Hospital, Boston, MA, United States; ^3^Memphis University, Psychology, Memphis, TN, United States

##### **Correspondence:** L. Grazzi

Patients with *High Frequency Migraine without Aura* (9/14 attacks per month) (HF-M/A) are particularly exposed to the risk of chronification and medication overuse. A multidisciplinary approach is suitable for these patients before than a chronic condition is induced. Recently, non-pharmacological approaches as Acceptance Commitment Therapy (ACT), showed efficacy in treatment of pain conditions and migraine, comparable to pharmacological prophylaxis at long-term, by promoting psychological flexibility and cultivating positive psychological capacities. Our aim was to assess the effectiveness of ACT model for HF-M/A. 35 patients were included and randomized for the study. Two treatment conditions: 1) TAU (Treatment as Usual): pharmacological prophylaxis (17 patients); 2) TAU + ACT (13 patients). ACT consisted of six 90-minutes weekly sessions, and 2 booster sessions, every 15 days; small groups of patients (7-10 patients each). Sessions included: psycho-education, mindfulness, experiential exercises, home assignments. At 12 months follow-up, results showed a decrease in days of headache /month in both groups; a decrease of medications intake/month in the ACT group, and a slight decrease in the TAU group. In conclusion, ACT seems beneficial for these patients. An integrated and flexible treatment program combining different approaches may be more effective than drugs alone to alleviate pain and reinforce clinical improvement.

## P0486 Prevalence and comparison of depression rates in the geriatric population of an old age home and a community, and its association with demographic factors

### S. Singhvi, P. Singh

#### Smt. NHLMMC and SVP Hospital, Ahmedabad, India

##### **Correspondence:** S. Singhvi

***Background and objective -***Depression is the most common mental health problem in the elderly. This adds severe burden on the patient, also affecting their families and their financial situation. Finding out the prevalence of depression among older adults living in an old age home and a community provides information about the impetus one should give on mental health. Therefore, the results of this study will help the entire health care community to understand the severity of depression in the geriatric age group, find the leading causes of depression and help with the intervention of the linkage.

***Methods*** *-*A cross section study of the geriatric group of population was performed, two sections of the geriatric group were taken for the study- geriatric population residing in an old age home (80) and geriatric population residing in a community (80). There were two forms used for data collection - a Geriatric Depression Scale (GDS), a standardized tool used to assess the level of depression and a demographic form was used to collect the demographic information. To find the association between different factors, the statistical method of Chi-square test and P-value was taken.

***Results*** *-* Given in the Tables below

***Conclusion*** *-* Increased attention to mental health care, especially in the geriatric population, should be encouraged. Further, multiple factors were found associated with depression and therefore preventive management of such factors should be our goal.

**Fig. 1 (abstract P0486).**
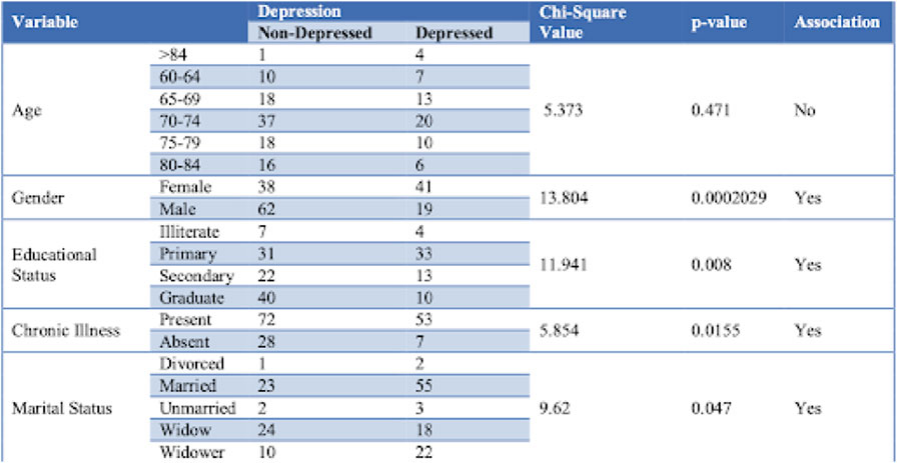
See text for description

**Fig. 2 (abstract P0486).**
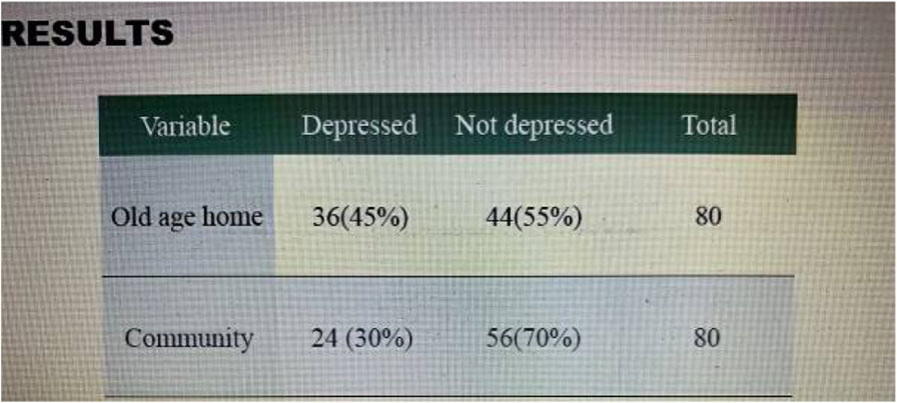
See text for description

## P0487 Efficacy and Safety of Occipital Nerve Stimulation and Occipital Surgical Decompression For Migraine - Systematic Review and Meta-analysis

### A. Shahid^1^, M. Abbasi^1^, J. Arturo Larco^1^, Y. Liu^1^, S. Madhani^1^, C. Robertson^2^, L. Savastano^1^, C. C. Chiang^2^

#### ^1^Mayo Clinic, Neurosurgery, Rochester, MN, United States; ^2^Mayo Clinic, Neurology, Rochester, MN, United States

##### **Correspondence:** A. Shahid

**Background**

Occipital nerve stimulation (ONS) and occipital surgical decompression (OSD) have been reported to be effective for medically refractory migraine. We conducted a systematic review and meta-analysis to analyze the efficacy and safety of ONS and OSD for the treatment of migraine.

**Methods**

We searched PubMed, Scopus, and Ovid Medline from 1990-January 2021 using keywords: occipital nerve surgery, occipital nerve stimulation, and migraine. Studies were included if mean change in headache frequency, intensity, and complication rate were reported. Studies were excluded if indications were not migraine and no interventions in the occipital region.

**Results**

11 studies (306 patients) on ONS and 6 studies (531 patients) on OSD reporting the change in headache frequency and intensity were included in the meta-analysis. There was no significant difference in mean decrease in headache frequency and headache intensity between ONS and OSD [frequency: 7.06 (CI 95%; 5.08 – 9.05) vs 8.31 (CI 95%; 1.13 – 15.50), p=0.74; intensity 3.20 (CI 95% 2.25-4.15) vs 4.73 (CI 95% 3.27-6.18), p =0.08]. Complication rate was 33.6 % (CI 95% 21.7%-47.9%) in ONS and 7.6 % (CI 95% 1.3%-33.7%) in OSD, p= 0.06)

**Conclusion**

We did not find a significant difference between the efficacy and safety of ONS and OSD for migraines. More data is needed to establish their role in refractory migraine. Standardized endpoints should be utilized for future studies, especially surgical intervention for migraine.

**Fig. 1 (abstract P0487).**
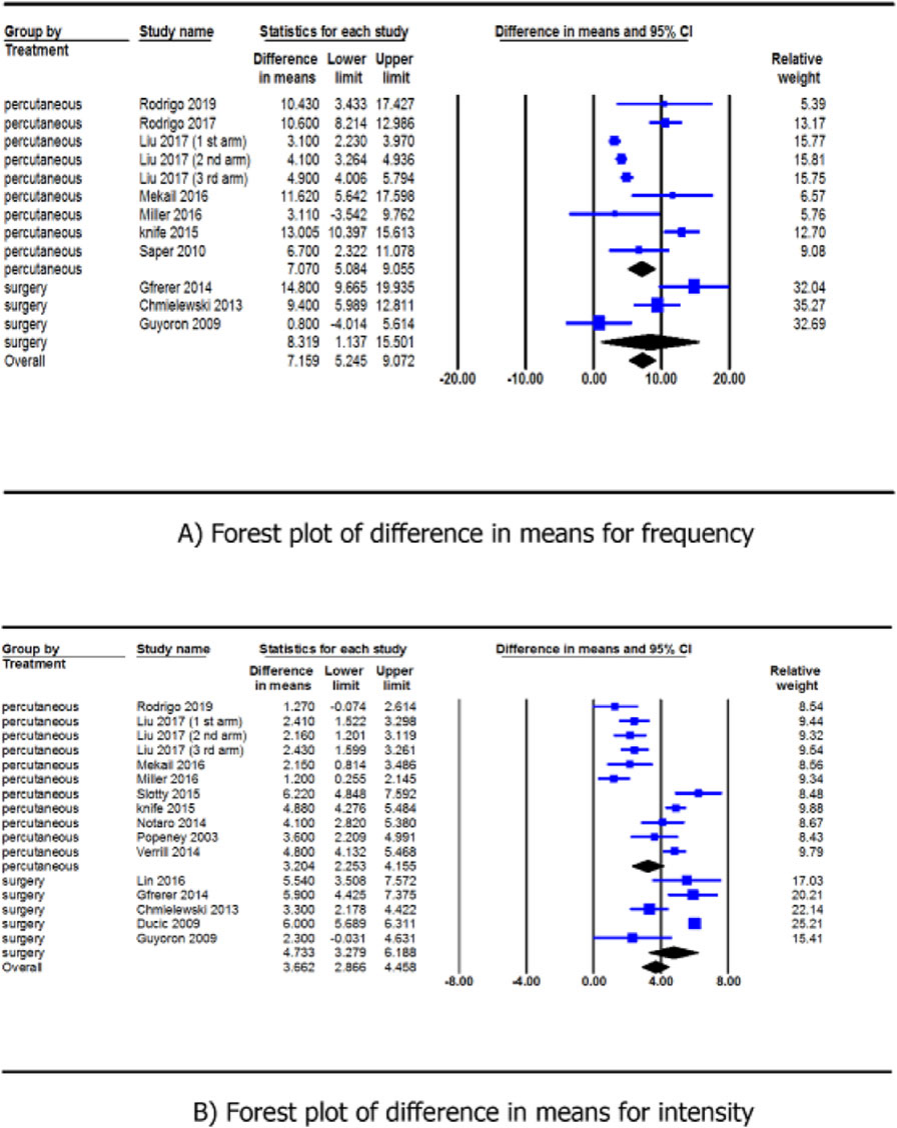
See text for description

**Fig. 2 (abstract P0487).**
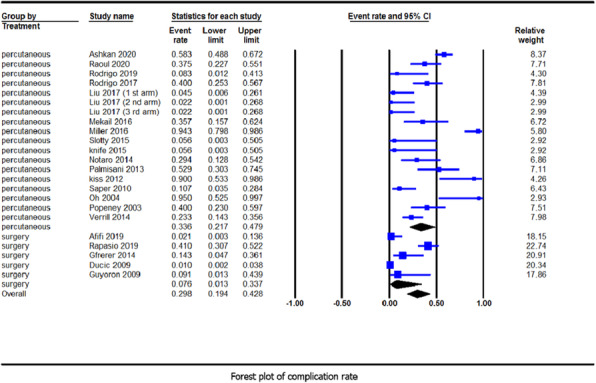
See text for description

## P0488 Prevalence of Premenstrual Syndrome and Migraine in Medical Students: comorbid disorders

### E. Melhado, S. Ozima Filho, T. Eschiapati, A. Gonçalves, A. de Matos, J. Picolo, J. Abdo, J. Christofoletti, P. Craice

#### Unifipa, Neurology, Catanduva, Brazil

##### **Correspondence:** E. Melhado

Background: Headache is a common symptom during the menstrual cycle. The primary trigger of Menstrually Related Migraine (MRM) seems to be the modifications in the estrogen levels. The present study seeks to associate headache with the menstrual cycle, specifically with Premenstrual Syndrome (PMS), relating its causes and aggravating factors. Objective: Verify if, among medical students with PMS, there is a higher prevalence of migraine headache; as well as assessing whether female students with headache have a higher prevalence of premenstrual syndrome. Method: Cross-sectional cohort study carried out on 189 women using a questionnaire. Results: Sample was by 189 young, single participants, students of higher education, users of contraceptives, similar ages of onset of PMS and headache (about 15 years old). It was observed 41% of the women presenting menstrual migraine “latu sensu”. Association was observed between headache and PMS, which is called comorbidity (close to 80% has headache and 81.5% has migraine). The risk of PMS was estimated to be 2.54 times higher in the population with headache. It was observed the link between PMS and menstrual migraine, with greatest occurrence of menstrual migraine in women with PMS. The association between HIT and the DSM-V was observed, so that the greatest number of PMS symptoms are observed among those with HIT => 50. Conclusion: Association between PMS and migraine is significant and high. These disorders are comorbid in the population studied. Neither MRM is not a risk fator for PMS, nor PMS is a risk factor for MRM.

**Fig. 1 (abstract P0488).**
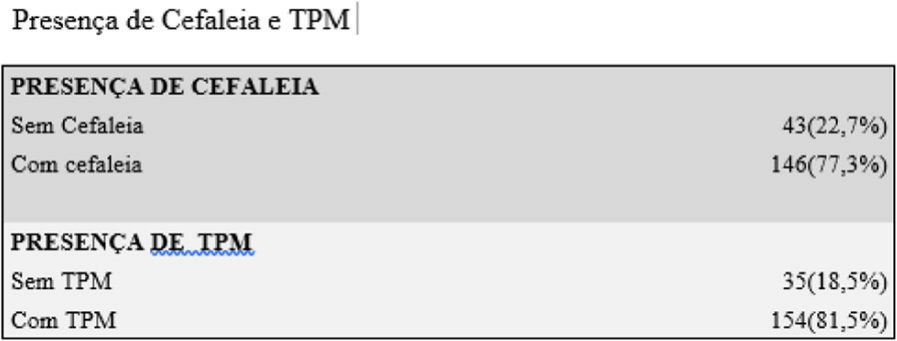
See text for description

## P0489 Double headache hemiparesis: Cerebral venous thrombosis with subarachnoid hemorrhage - A rare case report

### S. Dubey, Y. Sailaja, V. Ramakrishnan, C. Mutharasu

#### MMCH & RI, Department of Neurology, Kanchipuram, India

##### **Correspondence:** S. Dubey

Background and objective: Any young non hypertensive woman with risk of hypercoagulability presenting as worst headache of life and hemiparesis, despite the possibility of subarachnoid hemorrhage(SAH), MRI is the choice of neuroimaging to rule out cerebral venous thrombosis(CVT).

Methods: A 25 year old female presented with intolerable, disabling worst first headache of life, maximal at the onset since 2 days, 2 episodes of vomiting and associated with a staring look with impaired awareness for 5 min. followed by weakness of right upper and lower limbs, clinically suspected as an aneurysmal SAH. She was on hormonal therapy for polycystic ovary syndrome(PCOS) for few months.

Results: On neurological examination, there was right UMN facial palsy, 4/5 power in the right limbs. In view of the worst headache(SAH) and also to rule out any possibility of complications of hypercoagulable state, we opted MRI. MRI brain showed T2 FLAIR hyperintense foci in the sulcal spaces in both hemispheres. MR Venography showed no flow signals in the superior sagittal and transverse sinuses suggestive of cerebral venous thrombosis with subarachnoid hemorrhage.

Conclusions: In a non hypertensive and non traumatic case with risk of hypercoagulability presenting with first worst headache suspicious as SAH, we should maintain a high index of suspicion to rule out combination of other causes of life threatening headache like CVT. In these difficult circumstances, MRI is the choice of neuroimaging.

**Fig. 1 (abstract P0489).**
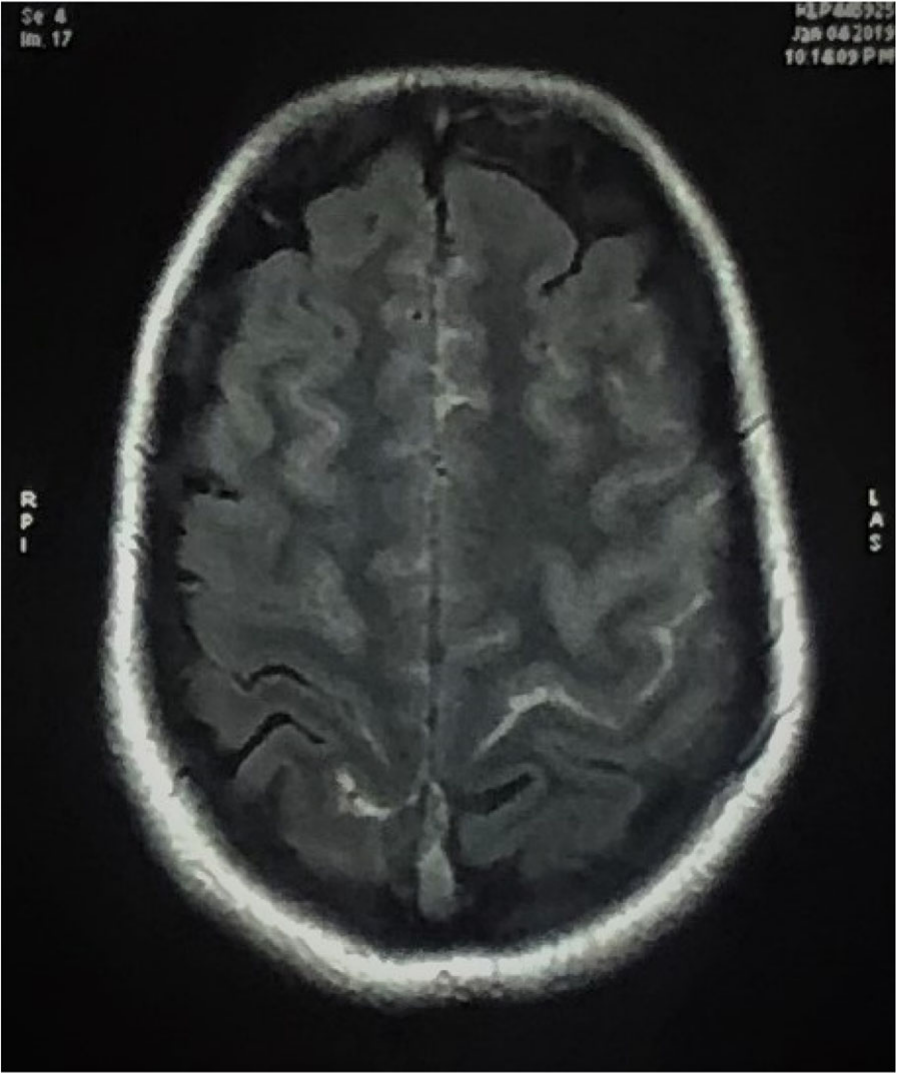
See text for description

**Fig. 2 (abstract P0489).**
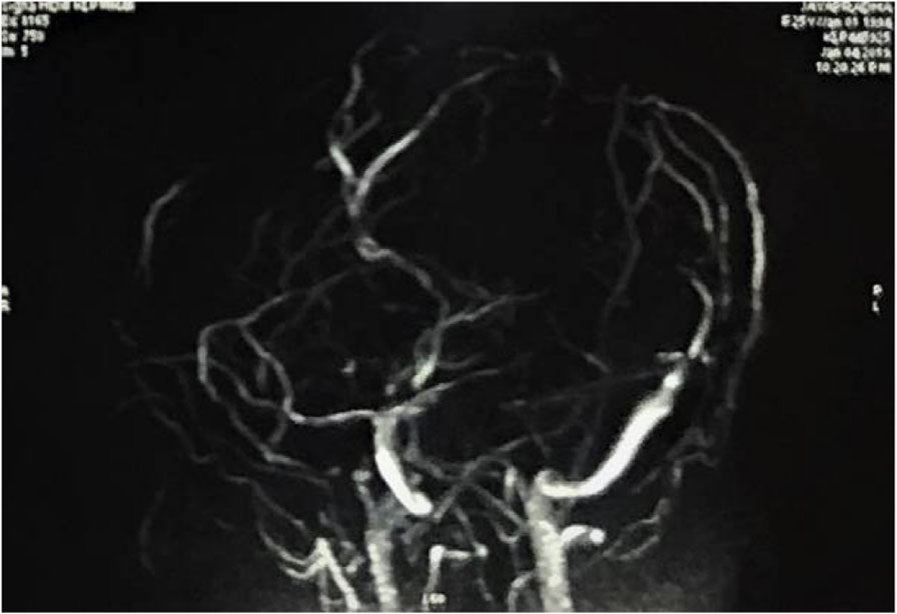
See text for description

## P0490 Development of a modern headache diary for headache management in Azerbaijan

### M. Mammadova^1^, S. Mirzayev^2^

#### ^1^Scientific Research Institute of Medical Rehabilitation, Clinical-neurophysiological laboratory, Baku, Azerbaijan; ^2^MediClub Clinic, Emergency, Baku, Azerbaijan

##### **Correspondence:** M. Mammadova

**Background and objective.** The lack of unified protocols for a headache diary creates a problem for the clinician to choose the right diary, to process data for maintaining the headache register. The purpose of our research was to develop and implement a headache diary for Azerbaijani patients in their native language. **Methods.** For patients (n = 30) with various forms of chronic headache, we used the headache diary developed by us in the Azerbaijani language in the form of a printed brochure. Patients noted diary items throughout the month. All signs were encoded with the abbreviation of a symptom or condition. **Results.** Among the 33% of patients who did not complete the diary, the reasons for noncompliance were a misunderstanding of filling items, failure to follow instructions (30%), late observation, and then forgetting symptoms (24%); the inconvenience of a printed brochure to fill out at work (46%). The identification of headache triggers in patients after using the diary was considered a positive result (15%). **Conclusion** The use of the resource in the native language was convenient for patients, but not portable. The creation of a mobile application for the developed diary in the native language will eliminate the problems that have arisen and speed up the formation of the register, and the placement of items on the visual interface will increase the interest of respondents and the sensitivity of the diary.

## P0491 Behaviour change techniques, theory and design of group self-management interventions for migraine: A systematic review

### A. M. Logan^1^, R. Hallett^2^, M. Edwards^1^, N. Greenwood^3^

#### ^1^St George’s University of London, London, United Kingdom; ^2^The Open University, Milton Keynes, United Kingdom; ^3^Faculty of Health, Social Care and Education, St George’s University of London and Kingston University, London, United Kingdom

##### **Correspondence:** A. M. Logan

**Objective**

Self-management interventions for migraine are known to reduce pain and disability compared with usual care but there is a lack of understanding about the components of those interventions.

This systematic review aimed to identify the active components of face to face migraine group self-management interventions, describing the underlying theory, design and Behaviour Change Techniques.

**Methods**

Five electronic databases were searched in January 2020 following PRISMA guidelines. Randomised controlled trials in English, of adult participants were included. Data were extracted using the Behaviour Change Taxonomy v1 for behaviour change techniques, Painter Criteria for theoretical content and recording intervention duration, timing, settings and outcomes.

**Results**

Three studies were included; one with high risk of bias, two studies with some concern. The studies were atheoretical, with heterogeneous outcomes and more than twelve years old. The results showed key behaviour change techniques used; *Instruction on how to perform a behaviour*, *Credible Source*. Two studies reported significant reductions in headache frequency and disability but trial design limited conclusions.

**Conclusions**

The review suggests self-management may be beneficial but there is a need for theoretically informed studies, identifying the active components of group self-management interventions for migraine.

## P0492 The C0–C2 axial rotation test – Reliability and correlation with the flexion-rotation test in people with cervicogenic headache and migraine

### K. Satpute^1^, K. Parekh^1^, T. Hall^2^

#### ^1^Smt. Kashibai Navale College of Physiotherapy, Musculoskeletal Physiotherapy, Pune, India; ^2^Curtin University, Perth, Australia

##### **Correspondence:** K. Satpute

**Objectives:** The C0–C2 axial rotation test (ART) is a measure of upper cervical rotation range of motion (ROM), reported to be reliable in a headache free population. The objective of this study was to determine intra- and inter-rater reliability of the C0–C2 ART and report normal values in people with a diagnosis of cervicogenic headache (CGH) or episodic migraine.

**Methods:** Two therapists independently evaluated rotation during the C0–C2 ART and flexion- rotation test (FRT) in 70 subjects (mean age 37.7 SD 11.6 years) with a diagnosis of CGH (35 subjects) or episodic migraine (35 subjects). An electrogoniometer was used to evaluate ROM. The FRT was assessed in supine with maximal neck flexion. Rotation ROM was assessed to each side. The C0–C2 ART was performed in sitting, neck in neutral, and rotation ROM assessed to each side with C2 vertebra stabilized.

**Results:** Reliability of the C0–C2 ART was moderate to high (ICC >0.70). The standard error of measurement and minimum detectable change for this test were at most 2°. In subjects with CGH mean ROM to the most restricted side was 9.3° (1.9) and 8.8° (2.1) for rater 1 and 2 respectively. In subjects with episodic migraine, mean ROM to the restricted side was 13.7° (1.6) and 13.6° (2.0) for rater 1 and 2 respectively.

**Conclusion:** The C0–C2 ART has at least moderate levels of reliability and correlates well with mobility determined by the FRT indicating the possibility of using this test when the FRT is not available.

## P0493 Cervical neuro-musculoskeletal impairments in people with cervicogenic headache: A systematic review and meta-analysis

### K. Satpute^1^, N. Bedekar^2^, T. Hall^3^

#### ^1^Smt. Kashibai Navale College of Physiotherapy, Musculoskeletal Physiotherapy, Pune, India; ^2^Sancheti College of Physiotherapy, Pune, India; ^3^Curtin University, Perth, Australia

##### **Correspondence:** K. Satpute

Objective: To identify cervical neuromusculoskeletal impairments those distinguish people with cervicogenic headache (CGH) from asymptomatic controls.

Methods: Eight databases were searched for studies which assessed subjects with CGH. Risk-of-bias and quality of evidence were assessed. Meta-analyses were performed for outcome measures of cervical neuro-musculoskeletal impairments using Review Manager 5.3.

Results: 16 studies out of 20 were rated as low risk of bias. In comparison with headache free controls the subjects with CGH presented with reduced combined cervical flexion-extension range of motion (ROM) (MD -17.17, 95% CI: -19.16, -15.13, I² = 87%), side flexion ROM (MD: -10.38, 95% CI: -11.92, -8.84, I² = 92%), and rotation ROM (MD: -14.91, 95% CI: -16.84, -12.98, I² = 96%), and upper cervical rotation ROM determined by the flexion-rotation test (MD: -14.98, 95% CI: -16.47, -13.48, I² = 30%). Similarly cervical flexor (MD: -33.70, 95% CI: -47.03, -20.37, I² = 0%) and extensor strength (MD: -60.23, 95% CI: -81.78, -38.68, I² = 0%) was reduced in subjects with CGH. In contrast, no difference was found in posture and kinaesthetic sense between symptomatic and asymptomatic people.

Conclusion: There is moderate to very low levels of evidence that subjects with CGH have restricted cervical ROM and reduced cervical flexor and extensor strength, endurance, and motor control, but lack postural abnormalities or loss of kinaesthetic sense.

## P0494 Clinical and Imaging Correlation in a Patient Series with Migrainous Infarction (MI) and Ischemic Stroke Related to Migraine (ISRM)

### L. Apostolakopoulou^1^, A. Tountopoulou^1^, S. Vassilopoulou^1^, G. Velonakis^2^, D. Mitsikostas^1^

#### ^1^Eginition Hospital, First Department of Neurology, Athens, Greece; ^2^National and Kapodistrian University of Athens, Second Department of Radiology, Athens, Greece

##### **Correspondence:** L. Apostolakopoulou

Objective: MI is a rare migraine complication. As ISRM we characterize the ischemic stroke that does not fulfill the strict MI criteria but is temporally related to a migraine attack. The objective is clinical and imaging correlation of a patient series with MI and ISRM aiming to expand current knowledge.

Methods: We describe seven patients with a history of migraine with aura, who exhibited a migraine attack with neuroimaging demonstrating ischemic infarction in a relevant area, while diagnosis was not better attributed to another ICHD-3 diagnosis. Medical history was obtained and clinical examination, complete stroke work up and magnetic resonance imaging were performed.

Results: The patients΄median age was 41 years and 71,4% were women, while 28,6% smoked and had patent foramen ovale. Aura types were visual in 28,6%, sensory and dysphasic in 57,1% and basilar in 14,3%. Ischemic lesions were located on one vascular territory, 71,4% posteriorly, 28,6% anteriorly, 57,1% being isolated, 42,9% multiple. Clinically, all patients exhibited mild neurological deficit and there was a consecutive reduction of migraine attack frequency and severity.

Conclusion: Current theories separate the mechanisms generating migraine related ischemia from thrombotic generated classic ischemia. This study does not provide data favoring one particular theory, but studying more cases of MI and ISRM will elucidate the pathophysiological basis and determine risk factors, prognosis and treatment.

**Fig. 1 (abstract P0494).**
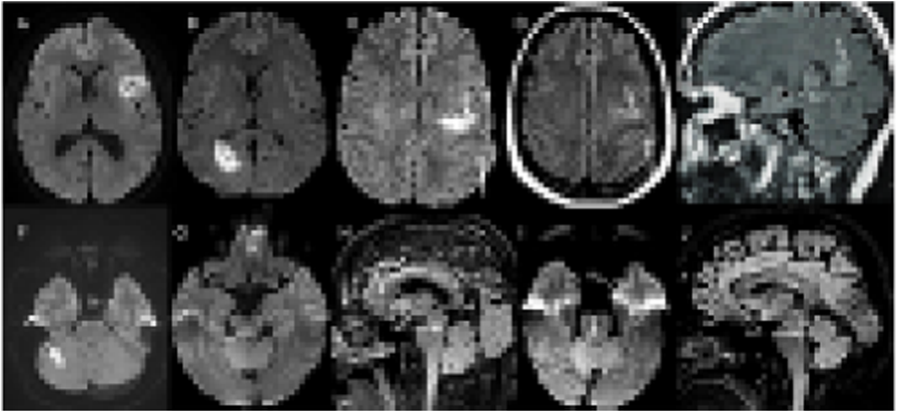
See text for description

**Fig. 2 (abstract P0494).**
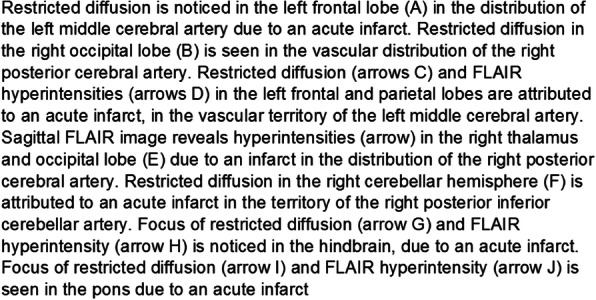
See text for description

## P0495 National Guidelines for the Treatment of Common Headaches Disorders in Pakistan

### A. Malik

#### LCMD, Medicine, Karachi, Pakistan

**Background**: Headache disorders are amid the peak 10 causes of disability. Migraine, tension-type headache, cluster headache and medication-overuse headache are essential in primary care; responsible for almost all headache-related burden. Management of these belongs largely in primary care. The prevalence of headache disorders in Pakistan is high and would be at least on par with that reported in other parts of the world, although no data are available for this country. With a population of 210 million, there would be at least 31 million patients with migraines alone (assuming a 15% prevalence).

**Methods**: To form treatment guidelines for common headache disorders, reviewed available guidelines, so as to develop own guidelines, specific to the needs of Pakistan.

**Results:** National guideline consists of: The globally IHS accepted definition and classification of common headache disorders with a simplified step wise approach to a patient with different types of primary headaches in Pakistan. Tables selecting the right drug with evidence-based references keeping in mind the cost/availability in Pakistan. Selection of medications in special populations e.g. young women, children and elderly. Use of onabotulinumtoxinA and the calcitonin gene related peptide antibodies for migraine prevention. Tables/Appendixes for easy access.

**Conclusion**: The guideline aims to provide the medical fraternity treating patients with common headache disorders with a step wise cost effective approach

**Fig. 1 (abstract P0495).**
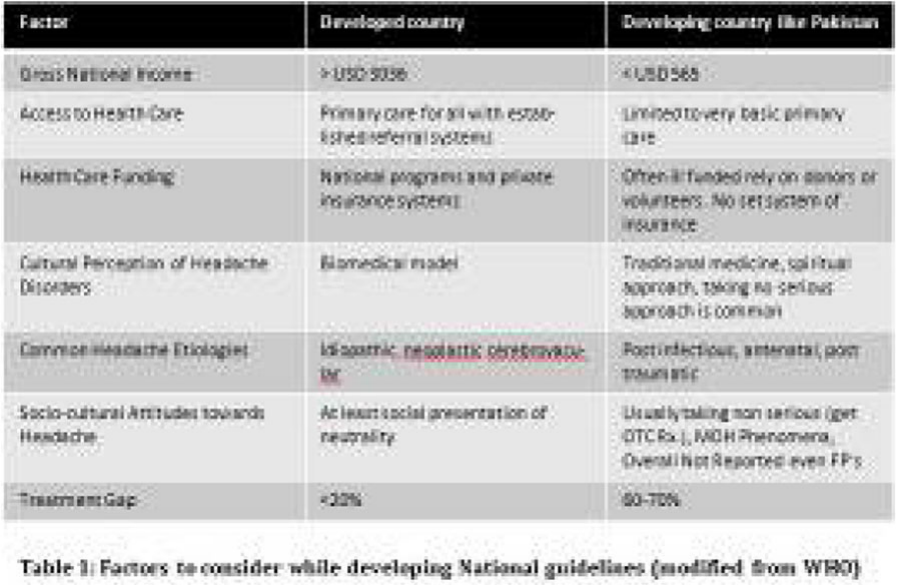
See text for description

## P0496 Severe abrupt (thunderclap) non-traumatic headache at the pediatric emergency department

### T. Eidlitz-Markus, Y. Levinsky

#### Schneider Children’s Medical Center, Headache clinci day hospitaliztion, Petach Tikva, Israel

##### **Correspondence:** T. Eidlitz-Markus

Background: Adult abrupt severe non-traumatic headache (thunderclap) is often related to serious underlying etiologies such as subarachnoid hemorrhage. However, data are sparse regarding thunderclap headache in the pediatric population.

Objective: The aim of the study was to evaluate the prevalence, characteristics and causes of thunderclap headache in the pediatric and adolescent population, aged 6-18 years, presenting to a pediatric emergency department.

Methods: The electronic database of a tertiary care pediatric emergency department was searched for children presenting with acute headache during 2016-2018. Headache severity was defined by pain scales, either a visual analogue scale or by the Faces Pain Scale–Revised.

Results: Thunderclap headache was diagnosed in 19/2290 (0.8%) of the included patients, all of them with a pain score of 10 /10. All the patients had a benign course. Primary headache was diagnosed in 15/19 (78.9%), Six patients had migraine and eight were diagnosed with primary thunderclap headache. Four of the 19 patients were diagnosed with secondary headache: three with infectious causes and one with malignant hypertension.

Conclusions: Thunderclap headache is rare among children and adolescents presenting to the emergency department. This headache is generally of a primary origin Extensive evaluation is still needed to rule out severe diagnosis problem.

## P0497 A simple method for transforming headache days to facilitate change modelling

### M. D. Villar-Martínez^1^, P. J. Goadsby^1,2^

#### ^1^King’s College, London, United Kingdom; ^2^University of California, Neurology, Los Angeles, CA, United States

##### **Correspondence:** M. D. Villar-Martínez

Headache frequency reduction is key aim any preventive treatment. Finding an appropriate statistical model to analyse the difference in monthly headache or migraine days as a marker of treatment response can be challenging due to the presence of non-positive integers, which cannot be easily handled by certain models. Our aim was to provide a simple approach to quantifying headache days for statistical analysis. Headache days are natural numbers by definition. When calculating treatment effects in a preventive study some subjects may have no effect, i.e. treatment difference zero, while others may worsen, i.e. negative treatment difference. Given headache days provides a discrete rather than continuous variable, the choice of a distribution is thus constrained. A transformation was considered that preserved the underlying metric and facilitated a suitable discrete distribution, such as the negative binomial distribution. Headache days over four weeks were transformed to a scale of 57 by adding 29 to the original number of days. These outcomes would start at number 1 (-28 days + 29) to number 57 (+28 days + 29). The value 0 would also be included as number 29 (0 days +29). Change over the treatment period is thus always a natural number. By transforming the number of days into natural numbers we eliminated non-positive integers without altering the metric in terms in outcome.

## P0498 Computerized Migraine Diagnostic Tools: A Systematic Review

### Y. Woldeamanuel, R. Cowan

#### Stanford University School of Medicine, Stanford Headache and Facial Pain Division, Stanford, CA, United States

##### **Correspondence:** Y. Woldeamanuel

Objective: To summarize and critically appraise all published studies involving computerized migraine diagnostic tools.

Methods: PubMed, Web of Science for [((computerized) AND diagnosis) AND migraine; ((automated) AND (migraine) AND diagnosis] was used to include articles in English that evaluated a computerized/automated migraine diagnostic tool. Development, sample size, sensitivity, specificity, reference diagnosis, quality of studies (QUADAS) were summarized.

Results: 38 studies (median sample size = 461 participants; median age = 42 years; 76% female) were included. Most (60%) tools were developed based on ICHD criteria, half were self-administered, and 88% were evaluated in headache centers using reference face-to-face interview-diagnosis (82%). The machine learning programs involved case-based reasoning, deep learning, classifier ensemble, ant-colony, artificial immune, white and black box combinations, hybrid fuzzy expert systems. The median diagnostic accuracy was: concordance = 75% (range 4-100%), sensitivity = 85% (33-100%), specificity = 83% (28-100%). 94% studies lacked random patient sampling. All studies avoided case-control designs. Most (74%) reference tests exhibited low risk of bias. Patient flow and timing showed low risk of bias in 81%.

Conclusions: Different computerized-automated migraine diagnostic tools are available with high accuracies. Random patient sampling and head-to-head comparison may improve their utility.

## P0499 Can an Artificial Intelligence Online Engine Diagnose Migraine as well as a Headache Specialist using a Semi-Structured Interview? A Multi-Center, Cross-Sectional Study

### R. Cowan^1^, A. Rapoport^2^, J. Blythe^3^, J. Rothrick^4^, K. Knievel^5^, A. Peretz^1^, E. Ekpo^6^, B. Sanjanwala^1^, Y. Woldeamanuel^1^

#### ^1^Stanford University School of Medicine, Stanford Headache and Facial Pain Division, Stanford, CA, United States; ^2^UCLA, Los Angeles, CA, United States; ^3^Information Sciences Institute, Los Angeles, CA, United States; ^4^George Washington University, Washington, DC, United States; ^5^Barrow Neurological institute, Phoenix, AZ, United States; ^6^UC Davis, Sacramento, CA, United States

##### **Correspondence:** Y. Woldeamanuel

OBJECTIVE: To assess the concordance in migraine diagnosis between an online, self-administered, computer-based, digital diagnostic tool (DDT) and semi-structured interview (SSI) by a headache specialist, both using ICHD-3 criteria.

METHODS: Participants completed two evaluations: phone interview conducted by headache specialists using the SSI and a web-based expert questionnaire, DDT. Participants were randomly assigned to one or the other protocol, with the second following one week after the first. The concordance in migraine/probable migraine (M/PM) diagnosis between SSI and DDT was measured using Cohen"s kappa statistics. The diagnostic accuracy of DDT was assessed using the SSI as reference standard.

RESULTS: 212 participant completed both SSI and DDT [median age = 32 years, female:male = 3:1]. Concordance in M/PM diagnosis between SSI and DDT was: κ = 0.832. DDT diagnostic accuracy: sensitivity = 90.1%, specificity = 95.8%. Positive and negative predictive values = 96.7% and 86.6%, respectively, using identified migraine prevalence of 60%.

CONCLUSIONS: The SSI and DDT have excellent concordance in diagnosing M/PM. Positive DDT helps rule in M/PM, through high specificity and positive likelihood ratio. A negative DDT helps rule out M/PM through high sensitivity and low negative likelihood ratio. DDT that mimics SST logic is a reliable and scalable tool for migraine diagnosis

## P0500 Changes in the quality of life after transsphenoidal endoscopic removal in patients with pituitary apoplexy

### E. Semina^1^, M. Kurnukhina^2^, V. Cherebillo^2^

#### ^1^First Pavlov State Medical University of St. Petersburg, General Medicine, St. Petersburg, Russian Federation; ^2^First Pavlov State Medical University of St. Petersburg, Neurosurgery, St. Petersburg, Russian Federation

##### **Correspondence:** E. Semina

**Summary**.Hemorrhage in the pituitary adenoma is the first manifestation of the disease in 80% of cases.The risk of hemorrhage in a pituitary tumor is 5,4 times higher than the risk of hemorrhage in another intracranial tumor.

**Purpose**.Assessment of changes in the quality of life of patients with pituitary apoplexy pituitary adenoma after transsphenoidal endoscopic removal.

**Materials and methods**. Our study included 200 patients with pituitary adenoma.Pituitary apoplexy was found in 2% of operated patients with recurrent pituitary macroadenomas.The subjects were aged 18-64 years.We used scale VAS,EORTC QLQ-C30.

**Results**.All patients with pituitary apoplexy complained of a pronounced diffuse headache in the preoperative period.The pain syndrome changed from 9,2±0,4 to 3±1,2 in the postoperative period.The study revealed a correlation between the pain syndrome and the indicators of various scales of the EORTC QLQ-C30.Before surgery patients with more pronounced pain syndrome more often indicated a deterioration in physical,cognitive,social,emotional functioning and general health(p<0,05).After surgery, the severity of headaches decreased in patients (before-82,1±16,4;after-17,2±8,1).After the removal of the formation,the patients,as well as before the operation, noted physical functioning with severe headaches(p<0,05).

**Conclusion**.Transsphenoidal removal in patients with pituitary apoplexy leads to an improvement in the quality of life, a decrease in the severity of pain syndrome.

## P0501 The analgesic role of manual therapy and exercise in management of tension-type headache. An update review

### S. E. Martín Pérez^1,2^, P. E. Barrera Singaña^3^, S. Pettineo^3^, R. Translateur Grynspan^3^, J. L. Alonso Pérez^1,2^, E. A. Sánchez Romero^2^

#### ^1^Universidad Europea de Canarias, Musculoskeletal Pain and Motor Control Research Group, Faculty of Health Sciences, La Orotava, Spain; ^2^Universidad Europea de Madrid, Musculoskeletal Pain and Motor Control Research Group, Faculty of Biomedical and Health Sciences, Villaviciosa de Odon, Madrid, Spain; ^3^Universidad Europea, Faculty of Biomedical and Health Sciences, Villaviciosa de Odon, Madrid, Spain

##### **Correspondence:** S. E. Martín Pérez

**Background and objective:** Tension-type-headache (TTH) is a condition characterized by a dull, non-pulsating, diffuse band-like pain in the head, scalp, or neck produced by an active myofascial trigger point (MTP). Our objective was to update the available evidence about the effectiveness of manual therapy (MT) in combination with exercise therapy (ET) to manage TTH. **Methods:** A systematic review was carried out according to PRISMA statement in database *MEDLINE* (*PubMed*), *PEDro*, *ScienceDirect,* and *Cochrane* using MeSH and free terms "*Tension-type headache*", "*musculoskeletal manipulations*", "*exercise therapy*", "*myofascial pain syndrome*" "*manual therapy*" and "*training*". Methodological quality and Risk of Bias were conducted by an independent researcher using PEDro Scale and ROB 2.0 tool. **Results:** 16 RCTs (n=1078, W:835; M:183; Mean Age: 35.7) were included with excellent interrater reliability (k=0.822). Robust evidence showed combining MT (*joint mobilization and suboccipital myofascial release*) and ET (a*erobic and strength exercise and relaxation training and postural reeducation*) decreased intensity of pain and reduced TTH episodes frequency. In addition, MT was effective in increasing pain pressure threshold (PPT), and range of motion (ROM). The combination of MT and ET improved disability and quality of life in the long term. **Conclusion:** MT showed to be effective on intensity, frequency, PPT, and ROM but its combination with ET improved disability and quality of life.

**Fig. 1 (abstract P0501).**
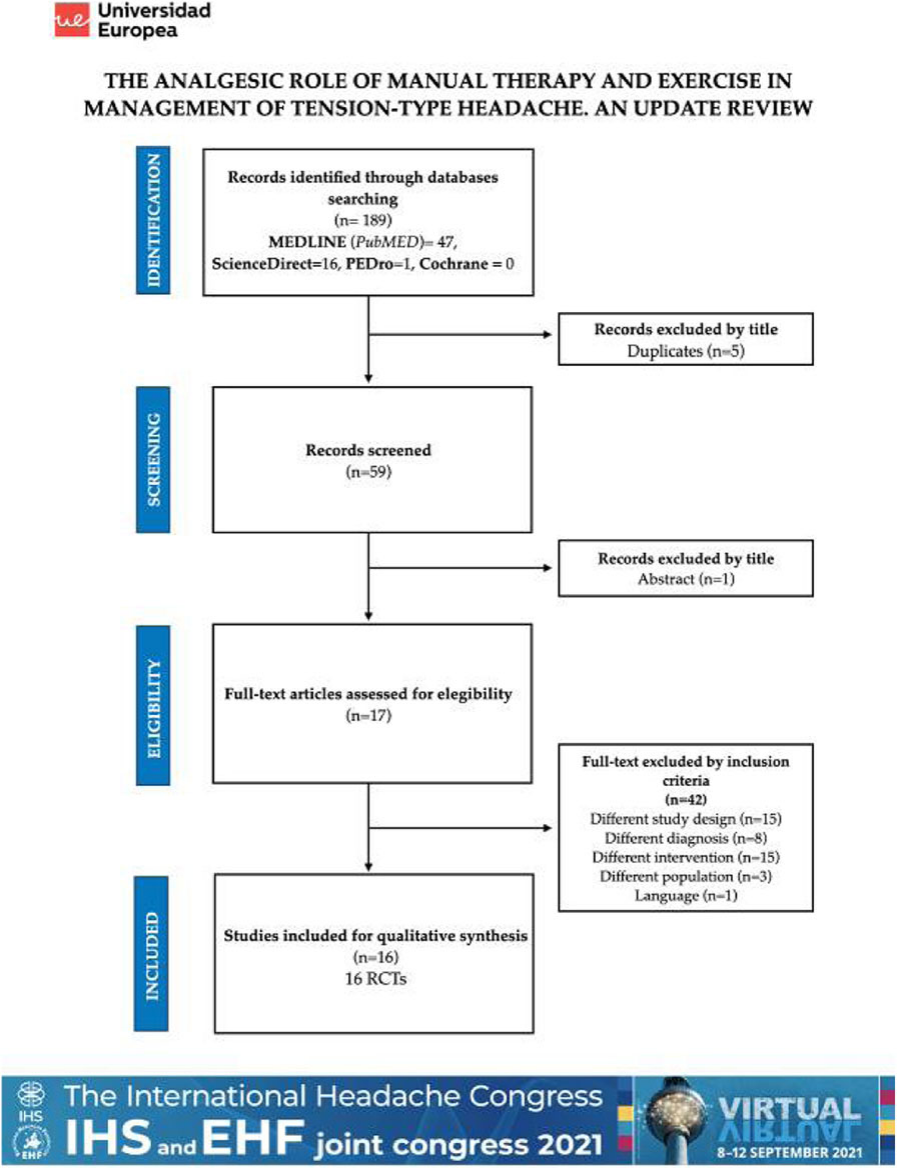
See text for description

## P0502 Genetic susceptibility loci in genome-wide association study of cluster headache

### A. V. E. Harder^1,2^, B. S. Winsvold^3,4,5^, R. Noordam^6^, L. Vijfhuizen^2^, S. Børte^3,4,7^, T. Hansen^8^, J. A. Zwart^3,4,7^, G. M. Terwindt^1^, A. van den Maagdenberg^1,2^, .. on behalf of the Cluster Headache Working Group^2^

#### ^1^Leiden University Medical Center, Department of Neurology, Leiden, Netherlands; ^2^Leiden University Medical Center, Department of Human Genetics, Leiden, Netherlands; ^3^Oslo University Hospital, Department of Research, Innovation and Education, Division of Clinical Neuroscience,, Oslo, Norway; ^4^Norwegian University of Science and Technology, K.G. Jebsen center for genetic epidemiology, Department of public health and nursing, Faculty of Medicine and health sciences, Trondheim, Norway; ^5^Oslo University Hospital, Department of Neurology, Oslo, Norway; ^6^Leiden University Medical Center, Department of Internal Medicine, Section of Gerontology and Geriatrics, Leiden, Netherlands; ^7^Oslo University Hospital, Institute of Clinical Medicine, Faculty of Medicine, Oslo, Norway; ^8^Rigshospitalet-Glostrup, Danish Headache Center, Department of Neurology, Glostrup, Denmark

##### **Correspondence:** A. V. E. Harder

**Objective:** Identifying common genetic variants that confer genetic risk for cluster headache.

**Methods:** We conducted a case-control study with cases from the Dutch Leiden University Cluster headache neuro-Analysis program (LUCA) study population (n = 840) and unselected controls from the Netherlands Epidemiology of Obesity Study (NEO) (n = 1,457). Replication was performed in a Norwegian sample of 144 cases from the Trondheim Cluster headache sample and 1,800 controls from the Nord-Trøndelag Health Survey (HUNT). Gene set and tissue enrichment analyses, blood cell-derived RNA-sequencing of genes around the risk loci and linkage disequilibrium score regression were part of the downstream analyses.

**Results:** Four independent lead SNPs (*r*^*2*^ < 0.1) were identified in relation to cluster headache with genome-wide significance (*p* < 5 × 10^-8^) and notably large effect sizes, rs11579212 (odds ratio (OR) = 1.51, 95% CI 1.33-1.72 near *RP11-815M8.1*), rs6541998 (OR = 1.53, 95% CI 1.37-1.74 near *MERTK*), rs10184573 (OR = 1.43, 95% CI 1.26-1.61 near *AC093590.1*), and rs2499799 (OR = 0.62, 95% CI 0.54-0.73 near *UFL1/FHL5*), collectively explaining 7.2% of the total variance of cluster headache. SNPs rs11579212, rs10184573 and rs976357, as proxy SNP for rs2499799 (*r*^*2*^ = 1.0), replicated in the Norwegian sample (*p* < 0.05).

**Conclusion:** This GWAS identified and replicated genetic risk loci for cluster headache with effect sizes larger than those typically seen in complex genetic disorders.

**Fig. 1 (abstract P0502).**
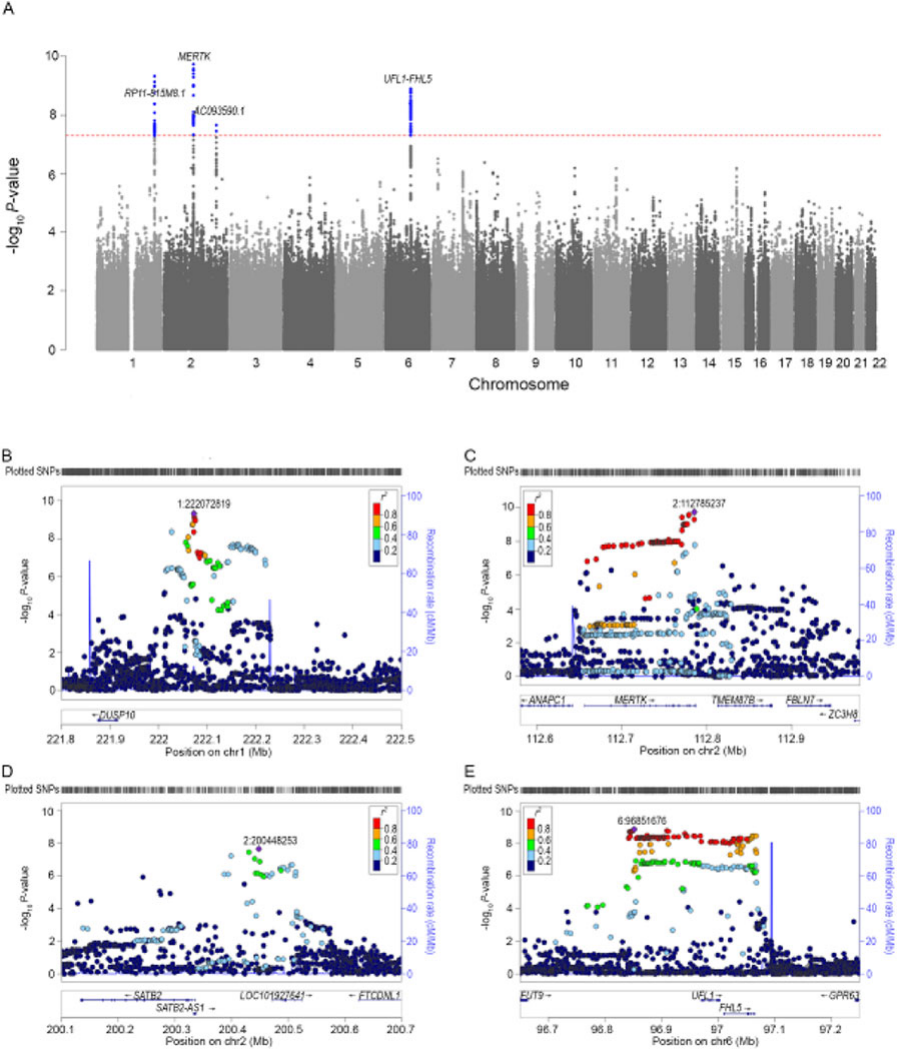
See text for description

**Footnote**

Abstract authors were asked to disclose their Conflicts of Interest. The disclosures are available via this link.

**Index**

**A**

Abbasi, M. P0487

Abdo, J. P0488

Abokalawa, F. P0421

Aguilar-Carreno, H. P0423

Ahn, J.-Y. AL067, P010, P011

Ain, N. A. A. P0479

Akçiçek, H. P0471

Al-Hashel, J. P0421

Alenezi, M. P0421

Alonso Pérez, J. L. P0501

Alroughani, R. P0421

Anand, S. P0440

Andersen, A. S. S. AL071, P0189

P0193, P025, P0429

Anderson, M. S. P0458

Andrasik, F. P0485

Apostolakopoulou, L. P0494

Arkuszewski, M. P0475, P0476

Arribas, J. P0469

Arturo Larco, J. P0487

Arzt, M. AL078

Aurjord, R. P0436

**B**

Bae, D. W. P0409

Baker, S. R. P0428

Banerjee, T. K. P0440

Bangs, M. P0408, P095

Barabanova, M. P0407, P0426

Barrera Singaña, P. E. P0501

Bartels, E. AL073

Basedau, H. AL061, AL062

AL069, P0447

Bedekar, N. P0493

Belin, A. C. AL070

Bendtsen, L. AL071, IL040, P0189

P0193, P025, P0429

Benkli, B. P0442

Berdnikova, A. P0464

Bernstein, C. P0485

Bertz, R. P0458

Beyeler, M. P0432

Bhardwaj, R. P0458

Blythe, J. P0499

Boinpally, R. P0474

Bolay, H. AL068, IL04, P02

Borbridge, L. P0474

Buchholz, D. P0423

Bulut, T. AL068

Burger, K. AL073

Burish, M. J. P0442

Butler, M. P0474

Byoungchul, C. P0439

Børte, S. P0502

**C**

Calabresi, P. P0453

Campoy, S. P0427

Carpay, J. A. P0460, P07

Carpinella, M. P0452

Carr, K. P0471

Castro Zamparella, T. P0452

Cavanagh, S. P0434, P0462

Chaudhuri, J. R. P0440

Cherebillo, V. AL064, P0128, P0194

P0392, P0425, P0500

Chiappiniello, A. P0453

Cho, S.-J. AL067, P010, P011

P0141, P0157, P0409

Cho, S. H. P0409

Choi, E. J. P0481

Choi, Y. AL067

Christensen, S. AL063

Christofoletti, J. P0488

Chu, M. K. AL067, P010, P011

P0141, P0409, P043

Chung, J. M. AL067, P010, P011

Chung, P.-W. AL067, P010, P011

Collins, J. L. P0458

Conci Magris, D. M. P0452

Consonni, M. P0406

Corbelli, I. P0453

Cordeiro, A. P0405

Cowan, R. P0498, P0499

Craice, P. P0488

Crema, S. P0434

**D**

de Coo, I. AL073, P07

de Matos, A. P0488

Dehghani Molander, L. P0483

Dettin, E. P0445

Dever, J. P0471

Diamond, M. AL077

Doesborg, P. AL073, P07

Dominguez, V. P0461

Dong, Y. AL076, P0265, P0408

Dongen, R. AL073

Dorr, F. P0434

Dubey, A. P0422, P0424

Dubey, S. P0422, P0424, P0489

**E**

Edwards, M. P0491

Eidlitz-Markus, T. P0438, P0496

Ekpo, E. P0499

Ekusheva, E. P0449

Empl, M. P0477

Ernstsen, C. AL063

Eschiapati, T. P0488

Evers, S. P0441, P0443

P0444, P092

**F**

Falkenberg, H. K. P0436

Falvo, M. Q. P0416, P0417

Farez, M. P0434

Farouk Ahmed, S. P0421

Fernández Slezak, D. P0434

Ferrari, M. AL073, P0475, P07

Filatova, E. P0464

Filipchuk, M. P0452

Finley, J.-a. P0458

Finnegan, M. AL077, P0294, P0295

P0296, P0301, P0382, P089

Frederiksen, J. L. AL071, P0429

Futter, N. P0457

**G**

Gallop, K. P0480

Gantenbein, A. AL078

Garcia Santos, R. A. P0415

Gaul, C. AL076, IL073, P0144

P0308, P0325, P0408

Gine-Cipres, E. P0465, P0466, P0467

Giniatullin, R. P0431

Giraldo, Y. P0461

Goda, H. P0437

Gokhale, S. P0440

Golshan, F. P0473

Gonçalves, A. P0488

Greenwood, N. P0491

Grignani, E. P0485

Grosu, O. P0418, P0419

Grunho, M. P0405

Guercini, G. P0453

Gülbahar, Ö. AL068

Guler Aksu, G. P0435

Guo, H. AL077, P0295, P0296

Gutiérrez, M. T. P0434, P0462

**H**

Haan, J. AL073

Hall, T. P0492, P0493

Hallett, R. P0491

Heinskou, T. B. AL071, P0189

P0193, P025, P0429

Henao, L. P0461

Henderson, L. P0451

Hensel, O. P0446

Hikita, T. P0437

Hirschberg, S. P0456

Hoffmann, J. P0457, P0468

Holland, P. R. P0455, P0456

Horta, M. P0482

Horta, W. P0482

Hougaard, A. AL066, IL061, P080

Houinato, D. P0445

Huerta, M. P0427

Hundemer, H.-P. P0443, P0444

Hussain, M. P0463

Huygen, F. AL073

**I**

Imbiakha, B. P0423

Int. Consortium for Cluster

Headache Genetics, C. AL070

Islam, M. K. P0414

Ito, K. P0437

**J**

Jansen-Olesen, I. AL063, P053, P057

Johnson, K. P0455

Johnston, A. P0471

Johnston, K. AL074, P0282

P0339, P0340

Jong-Hee, S. P0439

Jong-Ho, K. P0439

Joy, K. M. N. I. P0414

**K**

Kadymova, N. P0464

Karadaş, O. AL068, P02

Karsan, N. AL072

Kayar, O. P0435

Kennedy, G. P0478

Khalil, M. I. P0414

Khalil, M. P0463

Kim, B.-K. AL067, IL07, P010

P011, P0157, P0319, P0409

Kim, B.-S. AL067, P0484

Kim, S. AL067

Kim, S.-K. AL067, P011

Kim, Y. P0441

Klein, B. AL077

Kossi, O. P0445

Kowalczyk, N. AL074

Kudo, T. P0437

Kulkarni, R. P0440

Kundu, N. C. P0414

Kurnukhina, M. AL064, P0128, P0194

P0392, P0425, P0500

Kurt, E. AL073

**L**

Lagorio, M. P0416, P0417

Lanter-Minet, M. P0475

Larsson, H. B. W. AL066, P084

Lauria Pinter, G. P0406

Lee, D. G. P0481

Lee, J. P0410

Lee, K.-S. AL067, P011

Levinsky, Y. P0438, P0496

Lim, C. A. P0481

Lisicki, M. P0452

Liu, Y. P0487

Lo, S. H. P0480

Logan, A.-M. P0491

López Gonzalez, D. S. P0415

Löser, S. P0477

Luebke, A. P0423

**M**

Maarbjerg, S. AL071, P0189

P0193, P025, P0429

Madhani, S. P0487

Mahmud, R. P0414

Malik, A. P0495

Malm Hagen, S. P0483

Mammadova, M. P0490

Marciszewski, K. P0451

Marmura, M. J. AL077, P0323

Martín Pérez, S. E. P0501

Martinez, J. P0408

May, A. AL061, AL062

AL069, P0447

McAllister, P. AL077, P0106

P0294, P0316, P0320

McGeeney, D. P0474

McGrath, D. AL074

McVige, J. AL077, P0299, P099

Mechtler, L. AL077, P0299

P0327, P099

Mehnert, J. AL061, AL069, P0447

Meisingset, T. W. P0450

Melhado, E. P0488

Meyer, I. AL078

Meylakh, N. P0451

Miceli, R. AL077, P0294, P0295

P0296, P0301, P089

Mikleborough, M. P0473

Mirzayev, S. P0490

Moon, H.-S. AL067, P010

P011, P0157, P0409

Moreno-Ajona, D. P0457, P0468

Mulleners, W. AL073

Muñoz-Vendrell, A. P0427

Muresan, B. P0471

Mutharasu, C. P0424, P0489

Muzlaev, G. P0407, P0426

Myers Oakes, T. AL076, P0408

Mykland, M. S. P0450

**N**

Nagel, V. P0462

Naprienko, M. P0464

Nava, V. P0416, P0417

Nelson, A. M. P0441

Neverdahl, J. P. P0450

Ni Riordain, R. P0428

Nicholson, R. A. P0441

Nielsen, B. AL063

Noory, N. AL071, P0189

P0193, P025, P0429

Novick, D. P0443, P0444

Nunes Rabelo, N. P0459

**O**

O’Daly, O. P0448

Ogawa, Y. P0437

Ogunlaja, O. I. AL065

Oh, K. AL067, P010, P011

Omland, P. M. P0450

on behalf of the Cluster

Headache Working Group, . P0502

Oppermann, T. AL062

Özge, A. AL068, P02, P0435

Ozima Filho, S. P0488

Öztürk, B. AL068

**P**

Panni, T. P0443, P0444

Parekh, K. P0492

Pareto, D. P0454

Park, J.-W. AL067

Park, K.-Y. AL067, P011

Paulson, O. B. AL066

Pearlman, E. P0441

Peixoto, E. P0459

Peng, K.-P. AL062, AL069, P0447

Pereira, L. P0405, P042

Peretz, A. P0499

Petroulia, V. P0432

Pettineo, S. P0501

Piacentini, S. P0406

Pickering, T. P0456

Picolo, J. P0488

Popoff, E. AL074, P0282

P0339, P0340

Powell, L. AL074, P0282

P0339, P0340, P0480

Pucci, E. P0416, P0417

**R**

Rabbani, G. P0414

Raggi, A. P0485

Rahman, S. P0423

Ramakrishnan, V. P0424, P0489

Ramírez García Luna, A. S. P0415

Rapoport, A. P0499

Rasmussen, R. AL063

Riesenberg, R. AL076, P0408

Ritter, S. P0476

Rizzoli, P. P0411, P0412, P0485

Robertson, C. P0487

Rodríguez Rodríguez, M. S. P0415

Rothrick, J. P0499

Rovira, A. P0454

Rozwadowska, A. P0477

Ruedi, P. P0469

Ruiz Yanzi, M. A. P0434

**S**

Sailaja, Y. P0489

Sala-Padró, J. P0427

Sánchez Romero, E. A. P0501

Sand, T. P0450

Sang-Hwa, L. P0439

Sanjanwala, B. P0499

Sansone, E. P0485

Sarchielli, P. P0453

Satpute, K. P0492, P0493

Savastano, L. P0487

Schäfer, E. AL078

Schellong, M. AL069

Schraml, L. P0432

Scutelnic, A. P0432

Searl, M. P0485

Semina, E. AL064, P0500

Severt, L. AL077, P0292, P0293

P0294, P0295, P0296, P0299

P0301, P0474, P089

Shahid, A. P0487

Shapiro, R. E. P0413

Shepard, K. P0480

Silva, E. P0405

Singh, P. P0486

Singh, S. P0440

Singhota, S. P0430

Singhvi, S. P0486

Slavova, N. P0432

Smilkov, E. A. AL071, P0189

P0193, P0429

Smith, T. P0480

Sohn, J.-H. AL067, P010, P011

Song, T.-J. AL067, P011

Sonkaya, R. AL068

Spille, P. P0477

Spincemaille, G. AL073

Steiner, T. J. P0445

Stites, T. P0475, P0476

Stringfellow, J. P0458

Stroud, C. AL076, P0408

Sturm, L.-M. AL061, AL069

Sulaiman, W. A. W. P0479

Sureda-Gibert, P. P0456

**T**

Taborda, S. P0461

Tarducci, R. P0453

Taşdelen, B. AL068

Tchantchaleishvili, N. P0430

Teernstra, O. AL073

Teixidor-Panella, S. P0427

Telesca, A. P0406, P0411, P0412

Thorne, L. P0430

Thorud, H. M. S. P0436

Tountopoulou, A. P0494

Translateur Grynspan, R. P0501

Treuer, T. P0443, P0444

Trugman, J. M. AL077, P0262, P0263

P0281, P0294, P0295

P0296, P0301, P0382, P089

**U**

Uglem, M. P0450

Usai, S. P0406

**V**

Vandrovcova, J. AL070

Vassilopoulou, S. P0494

Velichko, I. P0407, P0426

Velonakis, G. P0494

Venda Nova, C. P0428

Vestergaard, M. B. AL066

Vijfhuizen, L. P0502

Vila-Pueyo, M. P0455, P0456

Villa, F. P0461

Villar-Martínez, M. D. P0457

P0468, P0497

**W**

Wei, D. Y. P0448

Wiest, R. P0432

Wilbrink, P. AL073

Wilkinson, V. P0463

Wille, F. AL073

Woldeamanuel, Y. P0498, P0499

Wu, J. P0430

Wu, Q. P0463

**Y**

Yakubova, A. P0431

Young-Suk, K. P0439

Younis, S. AL066

**Z**

Zagar, A. P0441

Zakrzewska, J. M. P0428, P0430

Zelaya, F. O. P0448

Zhang, W. P0442

Zrinzo, L. P0430

Zwet, E. AL073

Øie, L. R. P0450

